# Dual‐wield NTPases: A novel protein family mined from AlphaFold DB

**DOI:** 10.1002/pro.4934

**Published:** 2024-03-19

**Authors:** Koya Sakuma, Ryotaro Koike, Motonori Ota

**Affiliations:** ^1^ Department of Complex Systems Science Graduate School of Informatics, Nagoya University Nagoya Aichi Japan; ^2^ Institute for Glyco‐core Research, Nagoya University Nagoya Aichi Japan

**Keywords:** dark proteome, NTPase, protein discovery, structure mining

## Abstract

AlphaFold protein structure database (AlphaFold DB) archives a vast number of predicted models. We conducted systematic data mining against AlphaFold DB and discovered an uncharacterized P‐loop NTPase family. The structure of the protein family was surprisingly novel, showing an atypical topology for P‐loop NTPases, noticeable twofold symmetry, and two pairs of independent putative active sites. Our findings show that structural data mining is a powerful approach to identifying undiscovered protein families.

## 1 INTRODUCTION

Characterizing protein structures is essential for understanding the molecular basis of their function, and structures are typically solved by experimental approaches and deposited in the Protein Data Bank (PDB) (Burley et al., 2022). When the solved protein adopts a novel structure that appeared at the first time, the finding is usually reported by the researchers who determined it. However, more recently, public databases produced by state‐of‐the‐art structure prediction, such as the AlphaFold protein structure database (AlphaFold DB) and ESM metagenomic Atlas (ESM Atlas), are changing this situation (Lin et al., 2023; Varadi et al., 2024). These databases are approximately three orders of magnitude larger than the PDB and contain numerous experimentally unsolved protein structures. Structural models never seen by human beings must be hiddenly archived there since the models were generated automatically by artificial intelligences and deposited without any human curations, providing opportunities for finding novel proteins based only on the structural information in silico.

Dedicated data mining demands a clearly stated working hypothesis. While several groups have pursued intensive model classifications against AlphaFold DB (Barrio‐Hernandez et al., 2023; Bordin et al., 2023; Durairaj et al., 2023), this bird's‐eye approach could miss unique and intriguing proteins. To find these hidden gems, we defined a very specific database search question: are there monomeric proteins that contain multiple phosphate‐binding loops (P‐loops) on a single continuous β‐sheet? The P‐loop or Walker‐A motif is a local functional motif that recognizes phosphate groups and shared among P‐loop NTPases, such as ATPases, GTPases, and nucleotide kinases (NKs) (Leipe et al., 2002; Leipe et al., 2003; Saraste et al., 1990; Walker et al., 1982). In general, one P‐loop resides on a single continuous β‐sheet of a three‐layered α/β/α sandwich architecture. Our preliminary search against the PDB supported this observation because no structure has multiple P‐loops in a β‐sheet. However, the possibility that a single β‐sheet possesses multiple P‐loops should not be excluded. We hypothesized that such experimentally unobserved multiple‐P‐loop structures exist in AlphaFold DB and can be discovered via systematic data mining.

## 2 RESULTS

By computationally scanning more than 214 million entries in AlphaFold DB version 4 (Kim et al., 2023; Varadi et al., 2024), we extracted 15,977 single‐chained structures possessing multiple P‐loops. We then analyzed the hydrogen‐bond network and extracted 839 structures with multiple P‐loops on a single continuous β‐sheet (Frishman & Argos, 1995). The structures were grouped into 11 clusters based on structural similarity (Van Kempen et al., 2023). As a result, we found an uncharacterized family of P‐loop proteins, dual‐wield P‐loop NTPase (dwNTPase), as the largest cluster with 711 members. All structural models in this cluster were predicted with high confidence scores, that is, the average predicted Local Distance Difference Test was 94.27, indicating that the predictions were reliable (Figure S1) (Jumper et al., 2021).

**FIGURE 1:**
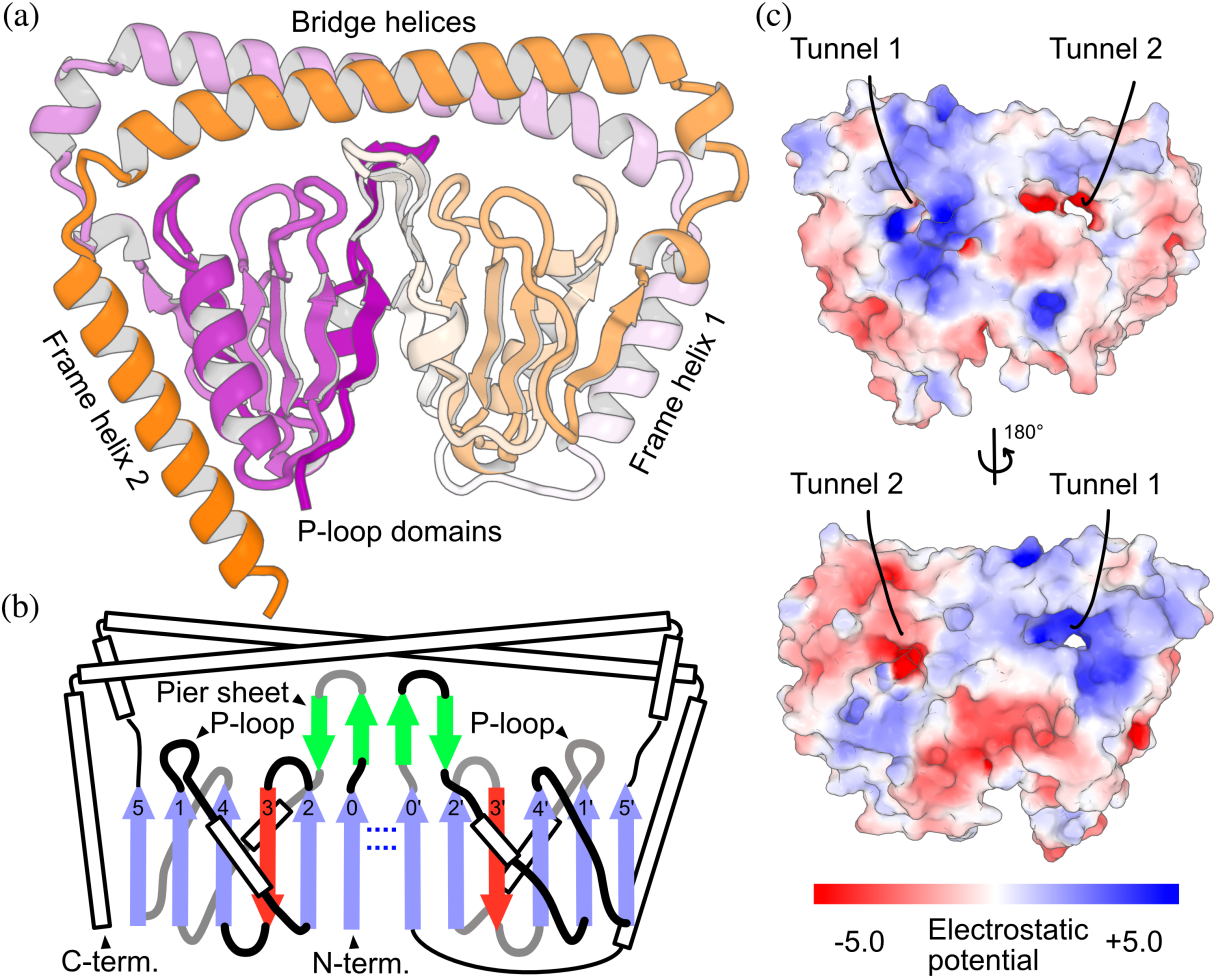
AlphaFold2 predicted model of a dual‐wield NTPase structure (AF‐A0A1Y0TWD8‐F1‐model_v4). (a) Overall dwNTPase structure colored according to a purple‐white‐orange gradient from the N‐ to C‐terminus. (b) Topology diagram of dwNTPase. Blue and red arrows represent β‐strands pointing up and down that form the large β‐sheets in the P‐loop domains. Green arrows represent the two pier sheets. White rectangles are α‐helices. Gray and black lines indicate junctions projecting behind and out of the β‐sheets, respectively. Blue dotted lines represent hydrogen bonds connecting the two halves of the large β‐sheet. (c) The location and shape of the ligand binding tunnels. Color bar is at the bottom.

The overall architecture of dwNTPases was novel and showed noticeable two‐fold symmetry. Figure 1a shows the structure of a representative dwNTPase from *Bacillus thuringiensis* (Bt. UniProt accession no. A0A1Y0TWD8). Two P‐loop domains are tightly packed and surrounded by two long bridging α‐helices and two framing α‐helices. The two bridging α‐helices cover the top side of two α/β P‐loop domains and form a coiled‐coil packing around residues 124–155 and 305–336 (Kumar & Woolfson, 2021). The C‐terminal α‐helices are packed to the N‐terminal domain forming very long‐range contacts, which means the symmetry in dwNTPase architecture does not result from tandem repeats of identical domains but involves more complicated exchange of secondary structural elements (SSEs; α‐helices and β‐strands) between them (see Section 3). Each of the domains comprises six β‐strands to form two β‐sheets. Since these two six‐stranded β‐sheets are connected by two hydrogen bonds between the C‐terminal end of strand 0 and its symmetrical counterpart, these parts form a continuous 12‐sheeted β‐sheet and reveal a previously unobserved dual‐P‐loop architecture (Figure 1b). Although the hydrogen bonds between two six‐stranded β‐sheets allowed us to identify dwNTPases structures during data mining, the interactions between them are so weak that the large β‐sheet may dissociate under realistic conformational fluctuations. Two canonical P‐loops independently form two putative ligand binding sites that penetrate through the molecule and resemble tunnels rather than pockets (Figure 1c). Two β‐hairpins from each domain form a pier‐like structure that looks like a planar “wall” between these two tunnels, but the β‐hairpins, which we call pier‐sheets, do not form a single four‐stranded β‐sheet as they have no hydrogen bonds between them. A search against the PDB clarified that no similar structures have been reported (Minami et al., 2018; Van Kempen et al., 2023). Similarly, a SwissProt subset of AlphaFold DB contained no similar structures (Minami et al., 2018; Van Kempen et al., 2023), indicating that the dwNTPase family has no reliable annotations manually verified by UniProt curators.

**FIGURE 2:**
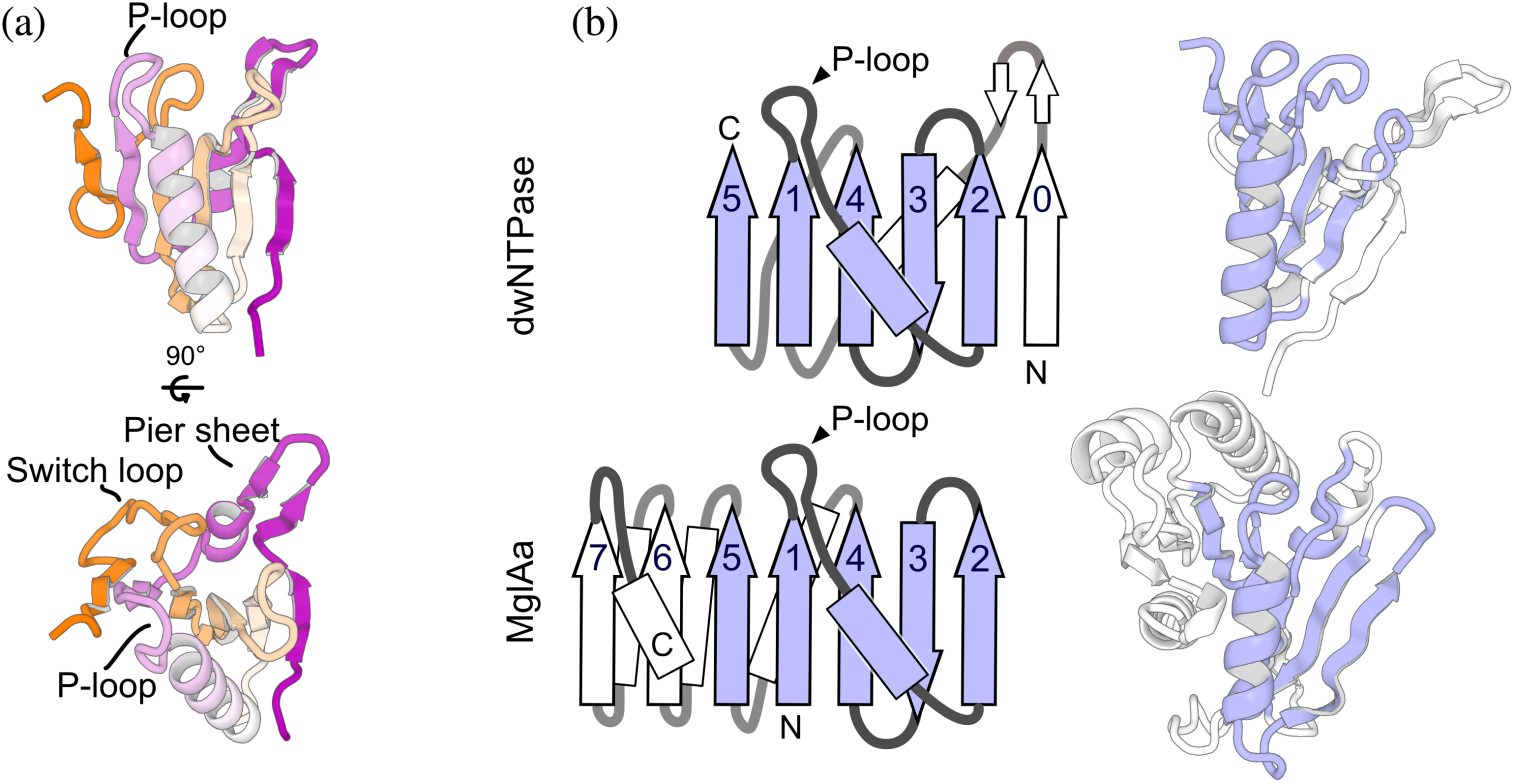
P‐loop domain. (a) Front and top views of the dwNTPase P‐loop domain colored according to a purple‐white‐orange gradient from the N‐ to C‐terminus. P‐loop, switch loop, and pier‐sheet are indicated by labels. (b) Topology diagrams and cartoon representations of dwNTPase P‐loop domains and MglAa structure. Arrows and rectangles represent β‐strands and α‐helices. Secondary structures that align between two structures are colored blue.

We found that the P‐loop domain of dwNTPases was structurally atypical for a P‐loop NTPase by searching against the PDB (Figure 2a) (Minami et al., 2018; Van Kempen et al., 2023). A crystal structure of mutual gliding‐motility protein MglAa from *Myxococcus xanthus* (PDB ID: 6h35), a bacterial small and monomeric GTPase, was the only known P‐loop NTPase that showed relevant structural similarity to the dwNTPase P‐loop domain (Galicia et al., 2019). The P‐loop domain of dwNTPase has an additional β‐strand at the N‐terminus (strand 0) compared to the MglAa structure (Figure 2b). Two strands constituting the pier sheet and a successive α‐helix are also appended. In contrast, the domain lacks two C‐terminal β‐strands (strands 6 and 7) and some other surrounding SSEs. These unique arrangements of SSEs give rise to the atypical topology that does not resemble other P‐loop NTPases (Figures S2 and S3) (Chandonia et al., 2022; Minami et al., 2018). Furthermore, the P‐loop domain has a long loop rather than a helix conserved in other P‐loop NTPases (Figure S4), which we named the switch loop (Figure 2a). These atypical features of the P‐loop domain make it difficult to assign dwNTPase to known classes of P‐loop NTPases.

Despite these novel features of dwNTPase, an iterative structure search by Foldseek against the entire AlphaFold DB revealed that 2219 similar structures were deposited, most of which originated from bacteria in various Firmicutes (Table 1 and Table S1) (Van Kempen et al., 2023; Varadi et al., 2024). Similar searches against ESM Atlas culled by 30% sequence identity found 748 similar structures (Table S2) (Lin et al., 2023). We classified dwNTPase structures into six subclasses based on the conservation of motifs and domains (Figure S5). The bona fide dwNTPase structure with two P‐loops intact (class 1) was the most abundant, suggesting functional constraints exist to conserve the two active P‐loops. A BLAST search against the nonredundant database revealed that dwNTPase had been classified as the PRK06851 family protein in the NCBI conserved domain database (McGinnis & Madden, 2004; Wang et al., 2023). Thus, we concluded that dwNTPases constitute a conserved protein family among bacteria.

**TABLE 1:** Phylogenetic classification of dwNTPases.

Domain	Phylum	Class	Count
Bacteria	Firmicutes	Bacilli	877
		Clostridia	705
		Desulfuribacillia	1
		Erysipelotrichia	5
		Negativicutes	13
		Tissierellia	32
		Unclassified Firmicutes	66
	Proteobacteria	Deltaproteobacteria	8
	Chloroflexi	Anaerolineae	1
	Tenericutes	Mollicutes	1
Archaea	Euryarchaeota	Methanomada group	1
Others			17

*Note*: We performed structural alignment of all 2219 structures against the representative dwNTPase structure. To ensure fragmented structures were excluded, 1843 structures showing TM‐scores >0.85 were selected. Entries with no phylogenetic information available in UniProt were ignored. The structures (1727 in total) were classified by their species. Others include environmental samples, metagenomes, unclassified bacteria, and Firmicutes from environmental samples.

## 3 DISCUSSION

**FIGURE 3:**
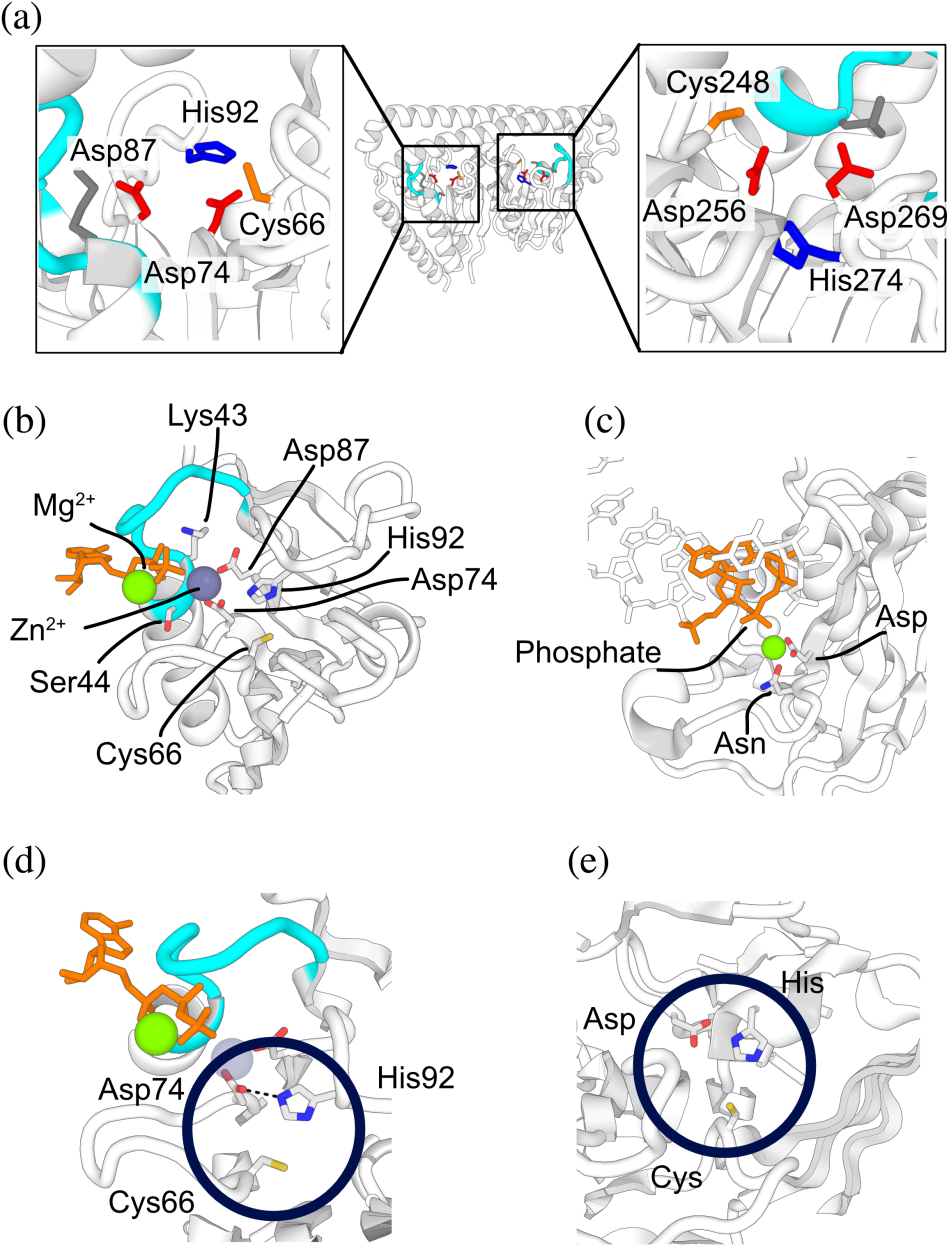
Putative functionally relevant residues. (a) Conserved residues in the putative ligand binding tunnels. His, Cys, and Asp are colored blue, orange, and red, respectively. P‐loops and their conserved residues are colored cyan and gray. (b) Coordination of metal ions by two aspartate side chains observed in MD simulations. ATP is shown in orange stick representation. Side chains of relevant residues are shown as sticks and CPK coloring. Green and gray spheres represent Mg^2+^ and Zn^2+^ ions, respectively. The P‐loop is colored cyan. (c) The active site structure of RNase H (PDB ID: 1zbl). The side chain of metal coordinating amino acid residues Asp and Asn are shown as sticks and CPK coloring, where Asn is a mutation from Asp. Mg^2+^ ions are shown as spheres. The Mg^2+^ ion coordinating with the side chain of Asp and Asn is colored green. Nucleic acid residues that contact the Mg^2+^ ion are shown in orange. (d) The catalytic triad‐like side chain configuration observed during the MD simulations. The triad‐like side chain cluster is circled. The black dotted line indicates the hydrogen bond between the side chains of His92 and Asp74, which HBPLUS detected. e, The active site structure of TEV protease (PDB ID: 1lvm). Side chains of the Cys‐His‐Asp catalytic triad are shown as sticks, CPK coloring, and circled. MD, molecular dynamics.

The molecular functions of dwNTPases were investigated by analyzing conserved residues. Although the sequence identities between both halves of dwNTPase structures are generally low (median; 23.1%), the most symmetric class of dwNTPases (class 1) possesses two clusters of conserved residues shared between both halves (Figure 3a and Figure S6). We found Cys66/Cys248 (residue numbers follow Bt. dwNTPase), Asp74/Asp256, Asp87/Asp269, and His92/His274 formed putative metal binding sites. Molecular dynamics (MD) simulations of the Bt. dwNTPase structure complexed with two ATPs, two Mg^2+^ ions, and two Zn^2+^ ions showed that the Zn^2+^ ions were stably coordinated by two aspartates and the γ‐phosphate group of ATPs (Figure 3b) (Abraham et al., 2015; Huang et al., 2017), which resembles the active site structure of metal‐dependent nucleotidyl‐transfer enzymes (Figure 3c) (Yang, 2008). The side chains of Cys66/Cys248 and His92/His274 remained unoccupied (Figure 3d), suggesting that they may have roles other than metal‐binding. As the pair of cysteine and histidine residues are reminiscent of the catalytic triad/dyad in cysteine proteases (Figure 3e), we hypothesize that dwNTPases have additional hydrolase/ligase activity (Dodson & Wlodawer, 1998).

In addition to these conserved residues, we identified other regions characteristic of dwNTPases. First, each P‐loop domain has conserved lysine residues (Lys36/Lys218) that precede the P‐loops and interact with two switch loops. Because the switch loop partially conceals the ligand binding tunnels (Figure S7a) and is highly flexible in MD simulations (Figure S7b), the conserved lysine residues may play sensor‐like roles to trigger NTPase activity, depending on the binding of other ligands to the tunnels. Additionally, the P‐loops are surrounded by several charged or polar residues that support the recognition of NTPs and Mg^2+^ ions (Figure S7c) and are not conserved in known P‐loop NTPases (Leipe et al., 2002; Leipe et al., 2003).

Two previous reports on gene knockout experiments suggest that dwNTPase (Cd630_32980 or CD3298) plays a role in accumulation of dipicolinic acid into spores of *Clostridioides difficile* (Kochan et al., 2017; Ribis et al., 2023). This is consistent with the fact that dwNTPases are distributed among various Firmicutes, especially among Bacilli and Clostridia (Table 1), which are known for spore‐formation. However, the detailed biological roles and molecular mechanisms of dwNTPases remain elusive because their structures show limited homology to NTPases with known functions. In other words, this indicates that dwNTPases are responsible for unique molecular mechanism to function. The twofold symmetry implies that the interaction partner of dwNTPases also possesses twofold symmetry, such as double‐stranded DNA, or that the cleft between two P‐loop domains recognizes ligand molecules in a similar manner to periplasmic heme‐binding proteins (Figure S8) (Mattle et al., 2010). When focusing on the regions around this cleft, one of two hydrogen bonds that connects two P‐loop domains' β‐sheets, N atom of residue 9 to O atom of residue 191, was broken in 19 final snapshots out of 20 MD trajectories. By contrast, another one, N atom of residue 191 to O atom of residue 9, was intact in 18 final snapshots. These observations reinforce our initial guess that interactions between two β‐sheets are weak under thermal fluctuations and also suggest possible functional asymmetry of two P‐loop domains. Asymmetry was also found in the amino‐acid composition of individual halves; the left half (residues 1–139 and 321–369) of the structure in Figure 1a is more positively charged than the right half (140–320), indicating that each half plays different functional roles (Figure 1c and Figure S9).

The evolutionary origin of dwNTPases is unknown. Although it is plausible that dwNTPases gained twofold symmetry via gene duplication, domain swapping, and gene fusion (Figure S10) (Hadjithomas & Moudrianakis, 2011; Toledo‐Patiño et al., 2019), the origin of the unique topology of individual P‐loop domains remains unclear. Detailed phylogenetic analysis may explain the evolution of P‐loop NTPases, including dwNTPases (Leipe et al., 2002; Leipe et al., 2003). Structural and biochemical studies are required and should provide greater insight into the biological significance of the dwNTPase family.

## 4 CONCLUSIONS

In summary, we demonstrated that structural data mining based on specific working hypothesis can discover uncharacterized protein families, for example, dwNTPase, and is a powerful approach to exploring dark proteomes (Perdigão et al., 2015; Taylor et al., 2009), the unwatched region of the protein universe, which will help and encourage the design of experimental studies.

## 5 MATERIALS AND METHODS

### 5.1 Identification of structures containing multiple P‐loop‐like fragments

AlphaFold DB (v4 UniProt) was downloaded from the Foldcomp database (Kim et al., 2023; Varadi et al., 2024). We used foldcomp version 0.0.2 installed via pip. P‐loop NTPase protein structures were extracted by converting the models into the sequences of ABEGO using a custom Python script, where A, B, E, and G, respectively, denote backbone dihedral angles (phi, psi) for α, β, left‐handed β, and left‐handed α on the Ramachandran plot (Wintjens et al., 1996). O denotes other conformations unassignable on the Ramachandran plot, typically a cis‐peptide conformation. Typical P‐loop (Walker‐A) motifs have conformations represented by EBBGAG or BBBGAG, both of which can be seen in the crystal structure of α and β subunits of bovine mitochondrial F1‐ATPase (chain A and chain D of PDB ID: 1bmf) (Abrahams et al., 1994). Because the P‐loop is a junction between a β‐strand and an α‐helix, we extended the ABEGO motifs to “BBBEBBGAGAAAAA” or “BBBBBBGAGAAAAA” and extracted all the structures containing any of them by sequence pattern matching. We then calculated the Cα root‐mean‐square deviations (RMSDs) of the matched substructures against the reference P‐loop fragment (residues 166–179 of 1bmf, chain A) using pair_fit command in PyMOL 2.5.0 and filtered out substructures with Cα RMSDs larger than 2.0 Å. We obtained 15,977 proteins containing multiple P‐loop‐like fragments and built a custom Foldcomp database for subsequent procedures using tar2db command from MMseqs2 (version 96b2009982ce686e0b78e226c75c59fd286ba450) (Kim et al., 2023; Steinegger & Söding, 2017).

### 5.2 Identification of dual‐wield NTPases

Visual inspection revealed that most structures with multiple P‐loop‐like fragments within a single chain were tandem repeats of known P‐loop NTPase domains connected by flexible linkers. Such proteins were excluded by analyzing structures using STRIDE2TOP (version 1.0) that enumerates β‐sheets in a protein structure based on the hydrogen‐bond definition given by STRIDE and reports the list of β‐strands in each of the β‐sheets. Assigning two nearest β‐strands flanking the P‐loop‐like fragment to the β‐strands in the list, we obtained 839 structures possessing two P‐loop‐like fragments on a single β‐sheet. These structures were clustered by TM‐score calculations (≧0.5) with Foldseek (version 5285cd11c335e1a0133ffd3e32f55ad6ff82f3cb) into 11 clusters (Van Kempen et al., 2023). The largest cluster contained 711 members, which corresponded to dual‐wield NTPases. For these structures, we performed all‐against‐all structure alignment using MICAN (version 2019.11.27) and defined the structure with the largest average TM‐score as the representative (AF‐A0A1Y0TWD8‐F1‐model_v4) (Minami et al., 2018).

### 5.3 Extraction of structures similar to dwNTPase from AlphaFold DB and ESM Atlas

We performed iterative structure searches using Foldseek (version 9b92c127ac27a546a0c31f19ea4f48339e790ca0) to enumerate as many structures that resemble dwNTPase as possible (Van Kempen et al., 2023). In the first stage, we performed a structure search against AlphaFold DB using all 711 structures initially mined from AlphaFold DB as queries. After removing overlapping structures, we obtained 1377 structures. Using these structures as seeds, we again performed a Foldseek search and obtained 135 new nonoverlapping structures. The third iteration of Foldseek search yielded some nonspecific hits. Therefore, we stopped this iteration, manually selected similar structures, and discarded the remaining structures. Consequently, we obtained 2219 dwNTPase structures from AlphaFold DB. When using Foldseek's internal functionality to perform iterative search with six times of iteration, enabled by the option ‐‐num‐iterations 6, we only obtained 2115 structures that constitute a strict subset of these 2219 structures. Similarly, we performed structural searches against the highquality_clust30 subset of ESM‐atlas using 711 structures found in AlphaFold DB as queries and obtained 748 structures with a TM‐score larger than 0.5 (Lin et al., 2023; Van Kempen et al., 2023; Xu & Zhang, 2010).

### 5.4 Whole structure search against the PDB and Swiss‐Prot subset of AlphaFold DB

To assess the novelty of the dwNTPase structure and gain insights into the function, we performed structural searches against PDB100 and the Swiss‐Prot subset of AlphaFold DB (version 4) using the Foldseek server in the TM‐align mode and the representative structure as the query (Burley et al., 2022; Van Kempen et al., 2023; Varadi et al., 2024). No relevant (TM‐score ≧ 0.5) hit was found among these databases. We used MICAN to perform rigorous one‐against‐all searches without pre‐filtering; however, no similar (TM‐score ≧ 0.5) structures were found among the PDB (2023‐09‐Jan) and the Swiss‐Prot subset of AlphaFold DB (version 2) (Minami et al., 2018).

### 5.5 Domain structure search against the PDB, Swiss‐Prot subset of AlphaFold DB, and SCOPe

We searched structures similar to the P‐loop domain of the representative structure (residues 1–110) against the PDB100 and the Swiss‐Prot subset of AlphaFold DB by using the Foldseek server (Van Kempen et al., 2023). No relevant hit was found. We used MICAN to perform a rigorous structure search without pre‐filtering against the PDB (2023‐09‐Jan) and the Swiss‐Prot subset of AlphaFold DB (version 2) (Minami et al., 2018). We obtained 358 and 2931 relevant hits (TM‐score ≧ 0.5) from the PDB and Swiss‐Prot. We performed clustering by MMseqs2 with sequence identity set at 35% and obtained 15 and 137 clusters (Steinegger & Söding, 2017). The alignments were checked by visual inspection of all cluster representatives. We found that some structures showed similar topology to the P‐loop domain of dwNTPase: 6h35, Q1DB04, and Q9UBK7 from the PDB and Swiss‐Prot, which are annotated as GTPase or GTP‐binding proteins (Galicia et al., 2019). The remaining hits showed RecA‐like topology and were not topologically identical to dwNTPase because the RecA‐like topology has an all‐parallel β‐sheet, whereas dwNTPases have anti‐parallel‐containing β‐sheets. Similarly, we performed structural comparisons against domain structures classified as G‐proteins (SCOP concise classification string: c.37.8), NKs (c.37.1), and RecA‐like proteins (c.37.11) in the SCOPe version 2.08 using MICAN (Chandonia et al., 2022; Minami et al., 2018). The groups of G‐proteins, NKs, and RecA‐like proteins contained 255, 212, and 118 parsed domain structures, respectively, and we selected the structures that showed the highest TM‐score in the group for visualization (Figure S2). Note that when we added residues 341–369 (an α‐helix) of the representative structure to its residues 1–110 as the p‐loop domain, we obtained no similar structure in any structural databases.

### 5.6 Calculation of sequence identities between two halves of dwNTPase structures

We selected 1903 structures with more than 340 residues from the set of dwNTPases extracted from AlphaFold DB. A structure was self‐aligned by MICAN in the rewiring mode, which ignores the sequential order of SSEs (Minami et al., 2018). The sequence identity was calculated based on the second‐best alignment by MICAN.

### 5.7 Identification of putative catalytic residues (conserved residues) and a side chain pattern search against the PDB

The potential function of dwNTPases was examined by performing a sequence search and alignment to identify conserved residues by HHblits (version 3.3.0) against UniRef30_2022_02 (Remmert et al., 2012; Suzek et al., 2015). After three iterations, 2687 sequences were extracted from the database. To exclude fragmented sequences most likely originating from partial matches to the P‐loop consensus motif, we removed aligned sequences with more than 10 gaps against the representative sequence and obtained a Multiple Sequence Alignment (MSA) with 138 sequences. From this MSA, the site‐wise entropy of the alignments was calculated to identify conserved residues, and the top 10 residues around the two tunnels were listed. We defined tunnel 1 as residues 61–100 and tunnel 2 as residues 243–282. From tunnel 1, residues 62, 66, 67, 69, 73, 74, 75, 87, 88, and 100 were identified. From tunnel 2, residues 244, 246, 247, 248, 252, 255, 256, 261, 263, and 274 were identified. According to the orientation of side chains toward the tunnel, we selected Cys66, Ser67, Asp74, and Asp87 as candidates for probable functional residues in tunnel 1. Similarly, Cys248, Asp256, and His274 were selected for tunnel 2. Considering the symmetry of the dwNTPase structure, Cys66/Cys248, Asp74/Asp256, Asp87/Asp269, and His92/His274 were considered clusters of functional residues in tunnels 1 and 2. We performed a side‐chain pattern search against the PDB using the strucmotif‐search program (version 0.18.1) to determine whether protein structures possessed similar side‐chain configurations (Bittrich et al., 2020). The set of residues Cys66, Asp74, Asp87, and His92 in the representative structure was selected as queries, and a search was performed against all structures in the PDB (2022‐28‐12), with the threshold for the structure similarity set to 1.0 Å. The side chain pattern search gave no hits and indicated that the putative catalytic residues have a novel configuration of conserved residues.

### 5.8 Docking of ATP, Mg, and Zn

We transplanted ligand structures from existing PDB structures to model the complex structures. The P‐loop region of an ATPase crystal structure (PDB ID: 6j18) was superposed to the P‐loop of the representative structure by MICAN in PyMOL, and the ATP and Mg^2+^ models were extracted (Minami et al., 2018; Schrodinger, 2015; Wang et al., 2020). Similarly, His125 from a zinc finger motif (PDB ID: 2hgh) was superposed to His92 and His274, and the coordinating Zn^2+^ ions were extracted (Lee et al., 2006). The extracted ligand molecules were merged with the representative structure.

### 5.9 MD simulations

MD simulations were performed by Gromacs version 2022.04 with the charmm36 force field (Abraham et al., 2015; Huang et al., 2017). The size of simulation boxes was determined by the molecule size with margins of 13 Å. After in vacuo energy minimization to remove steric clashes, the protein‐ligand complex was solvated by the TIP3P water model with 0.1M NaCl, and the system was neutralized by adding additional Na^+^ or Cl^−^ ions, depending on the total charge of the protein and ligands. The energy was minimized by the steepest descent and equilibrated by 100 ps NVT and NPT simulations with harmonic restraints on the nonhydrogen atoms. The temperature and pressure of the system were controlled to 300K and 1 bar by the V‐rescale thermostat and Parrinello–Rahman barostat. Electrostatic interactions were computed by the particle mesh Ewald method, and bonds involving hydrogen atoms were constrained by the LINCS algorithm. For each docked model, we performed 20 trajectories of 100 ns simulations with a 2‐fs time step.

### 5.10 Figure preparation

The images of molecular structures were created by PyMOL and Mol* viewer (Schrodinger, 2015; Sehnal et al., 2021). The surface electrostatic potential was calculated by the PyMOL APBS plugin (APBS version 1.5) (Jurrus et al., 2018). Hydrogen bonds were detected by HBPLUS (version 3.2) and visualized by PyMOL (McDonald & Thornton, 1994). Secondary structure elements were assigned by DSSP (version 2.0.4) and illustrated by ESPript (version 3.1) (Kabsch & Sander, 1983; Robert & Gouet, 2014).

## AUTHOR CONTRIBUTIONS

**Koya Sakuma:** Conceptualization; methodology; software; data curation; investigation; validation; formal analysis; supervision; visualization; project administration; writing – original draft; writing – review and editing. **Ryotaro Koike:** Funding acquisition; supervision; writing – review and editing. **Motonori Ota:** Supervision; resources; funding acquisition; writing – review and editing; writing – original draft; data curation.

## Supporting information


**Figure S1.** Model confidence of initially mined dwNTPase structures.
**Figure S2.** Structure of the dwNTPase P‐loop domain compared with other representative P‐loop NTPase protein structures.
**Figure S3.** Topology diagram of the dwNTPase P‐loop domain compared with other representative P‐loop NTPases.
**Figure S4.** Comparison of the switch loop with the helical region conserved among P‐loop NTPases.
**Figure S5.** Variations in the dwNTPase structure.
**Figure S6.** Sequence logo of dwNTPase.
**Figure S7.** Other characteristic residues and substructures found in dwNTPases.
**Figure S8.** Comparison with periplasmic heme‐binding proteins.
**Figure S9.** Distribution of the net charge in the left and right halves of the dwNTPase structure.
**Figure S10.** A possible evolutional trajectory to realize two‐fold symmetry of dwNTPase architecture.

**Table S1.** List of AlphaFold DB entries structurally related to dwNTPase family. First column stores the Uniprot accession codes of the protein, and the second column stores the organism or resource names.

**Table S2.** List of ESM metagenomic Atlas entries structurally related to dwNTPase. Note that the target database was culled by sequence similarity of 30% identity and predicted Local Distance Difference Test threshold.

## Data Availability

STRIDE2TOP program is available at: https://github.com/GeorgeChikenji/stride2top. All the scripts (python, R, and bash) used in this study are available at: https://github.com/yakomaxa/mining_dual-wield_NTPases.
