# Supplementary material for: Dual‐wield NTPases: A novel protein family mined from AlphaFold DB
Source: Protein Sci. 2024 Mar 19;33(4):e4934. doi: 10.1002/pro.4934 (PMC10949312; doi:10.1002/pro.4934)
Supplement: Supplementary file 1 — Figure S1. Model confidence of initially mined dwNTPase structures. Figure S2. Structure of the dwNTPase P‐loop domain compared with other representative P‐loop NTPase protein structures. Figure S3. Topology diagram of the dwNTPase P‐loop domain compared with other representative P‐loop NTPases. Figure S4. Comparison of the switch loop with the helical region conserved among P‐loop NTPases. Figure S5. Variations in the dwNTPase structure. Figure S6. Sequence logo of dwNTPase. Figure S7. Other characteristic residues and substructures found in dwNTPases. Figure S8. Comparison with periplasmic heme‐binding proteins. Figure S9. Distribution of the net charge in the left and right halves of the dwNTPase structure. Figure S10. A possible evolutional trajectory to realize two‐fold symmetry of dwNTPase architecture. [file PRO-33-e4934-s001.docx]

Supplementary materials for “**Dual-wield NTPases: a novel protein family mined from AlphaFold protein structure database”**


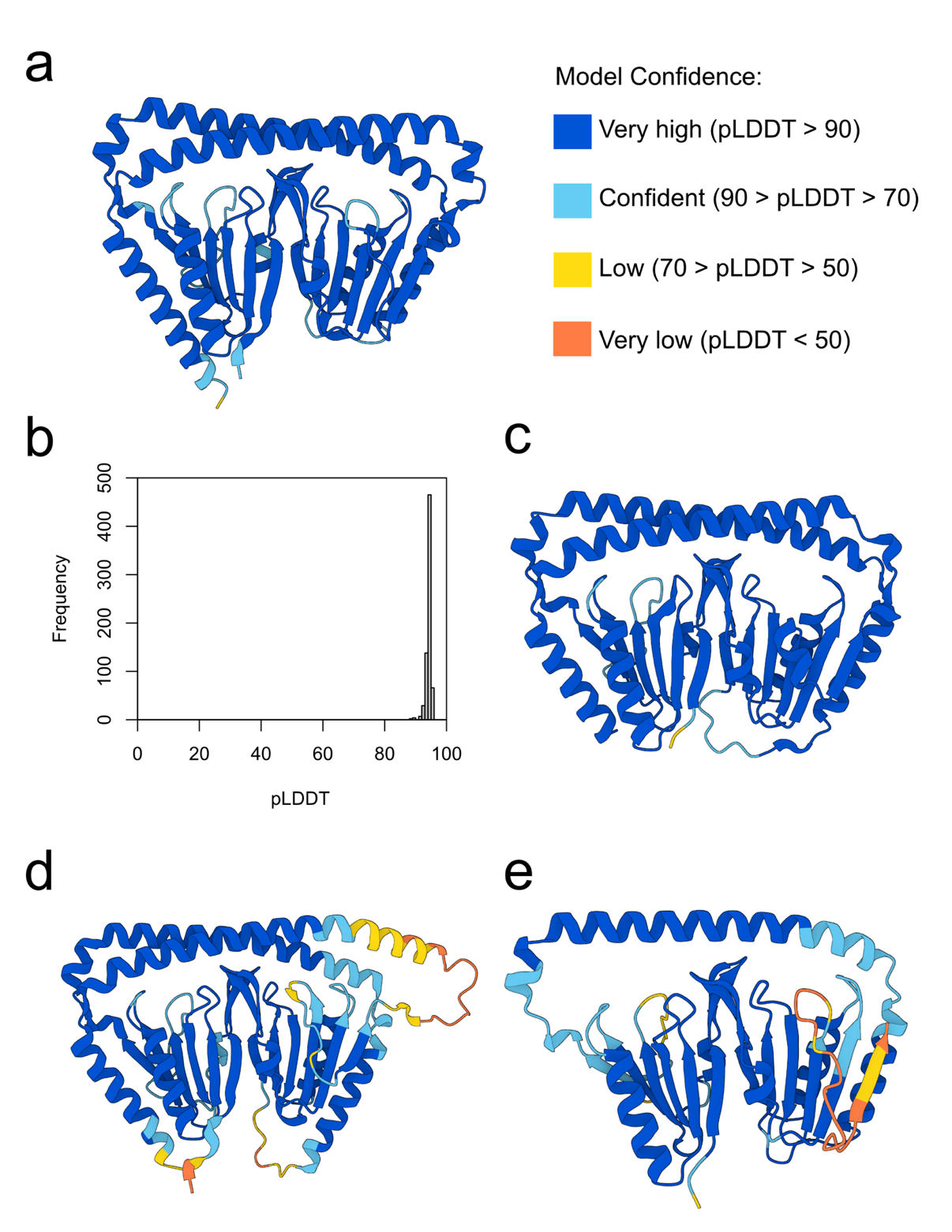


**Supplementary figure 1 | Model confidence of initially mined dwNTPase structures.** **a**, The cartoon shows the representative dwNTPase structure (UniProt accession No.: A0A1Y0TWD8). The cartoon is colored according to the residue-wise values of pLDDT. The coloring scheme is shown on the right of the structure, which is the same as displayed in AlphaFold DB. **b**, Distribution of total pLDDT values among the 711 dwNTPase structures initially mined from AlphaFold DB. **c–e**, The most confident, second worst and worst predictions among the 711 dwNTPase structures (A0A7V6TLZ4, A0A7C6J2V5 and W4RL53) gave average pLDDTs of 95.67, 88.43 and 88.01, respectively. Structures were created by Mol* viewer.


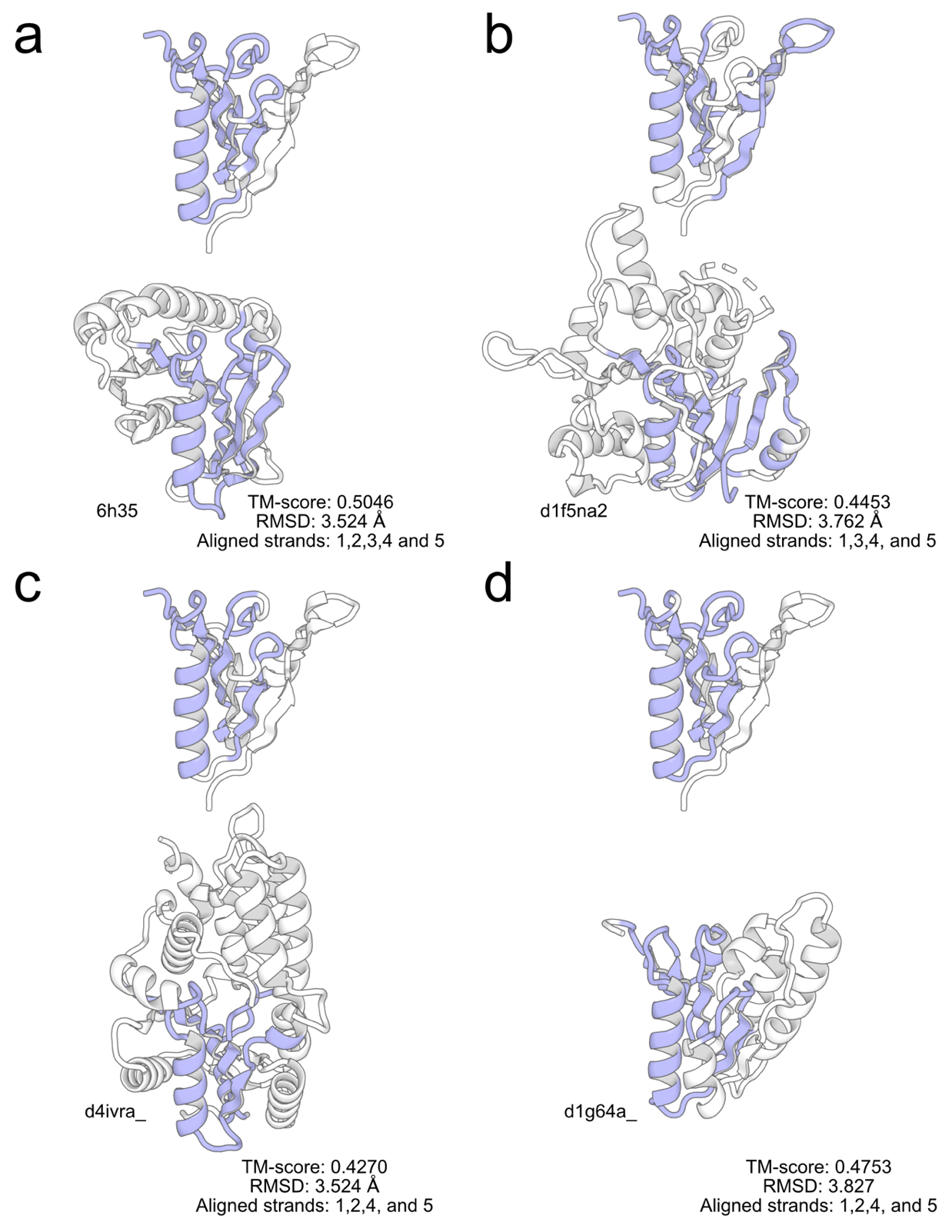


**Supplementary figure 2 | Structure of the dwNTPase P-loop domain compared with other representative P-loop NTPase protein structures.** Aligned structures of the dwNTPase P-loop domain and known NTPases. **a**, MglAa, (**b**) G-proteins, (**c**) NKs and (**d**) Rec-A like. The P-loop domain of dwNTPase and known NTPase are shown on the top and bottom of the panels, respectively. The latter three structures were selected from c.37.8, c.37.1 and c.37.11 SCCSs in SCOPe that showed the highest similarity to the dwNTPase P-loop domain. The aligned regions are colored blue. The PDB or SCOPe IDs are shown below the structures. The RMSD, TM-score and list of aligned strands in the dwNTPase P-loop domain are summarized at the bottom of the panels.


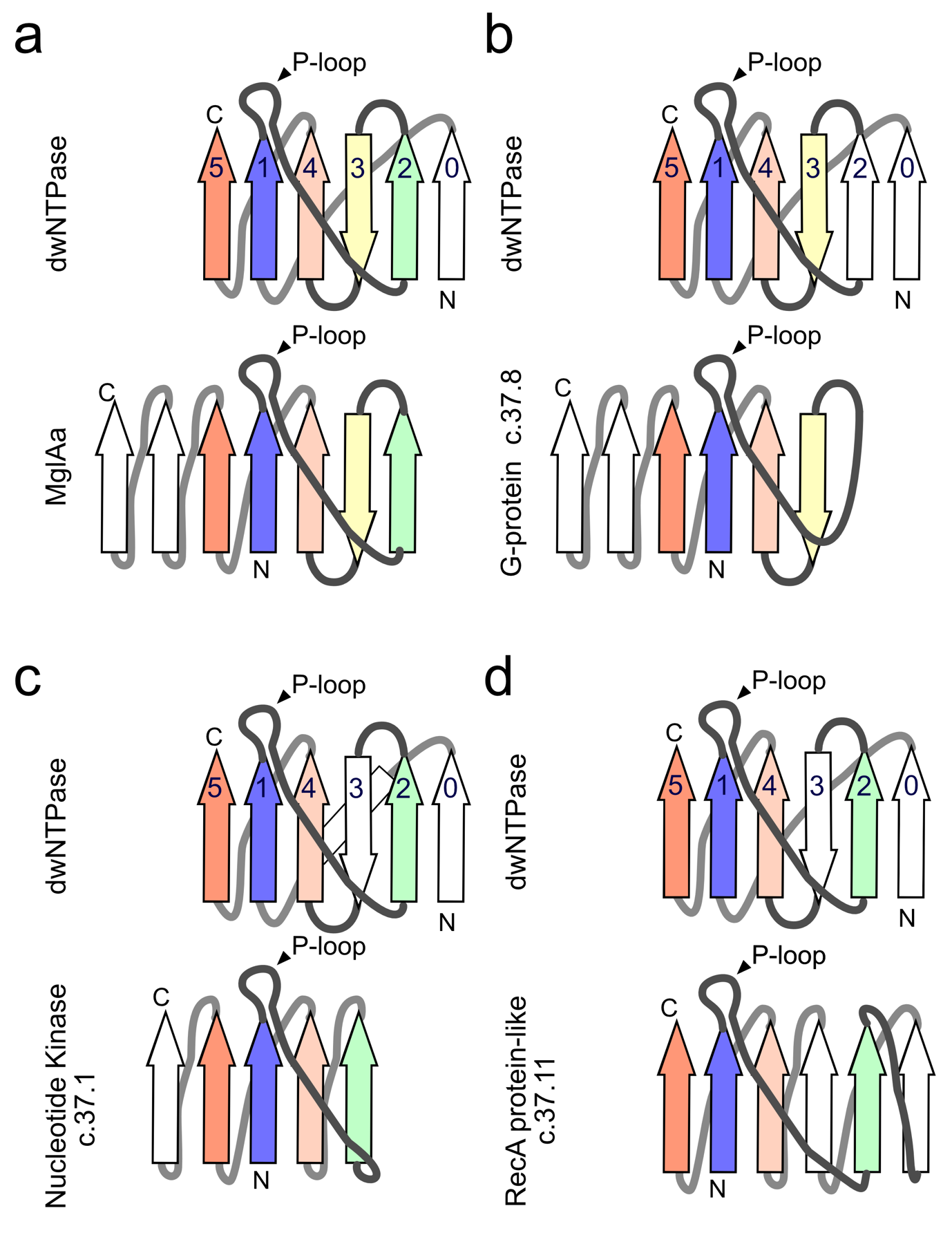


**Supplementary figure 3 | Topology diagram of the dwNTPase P-loop domain compared with other representative P-loop NTPases.** **a**, Comparisons with MglAa, (**b**) G-proteins, (**c**) NKs and (**d**) RecA-like proteins. Arrows represent strands. Grey and black lines indicate junctions projecting behind and out of the β-sheet. The pair of aligned strands are colored in the same colors. Unaligned strands are colored white. Helices are omitted for clarity.

**
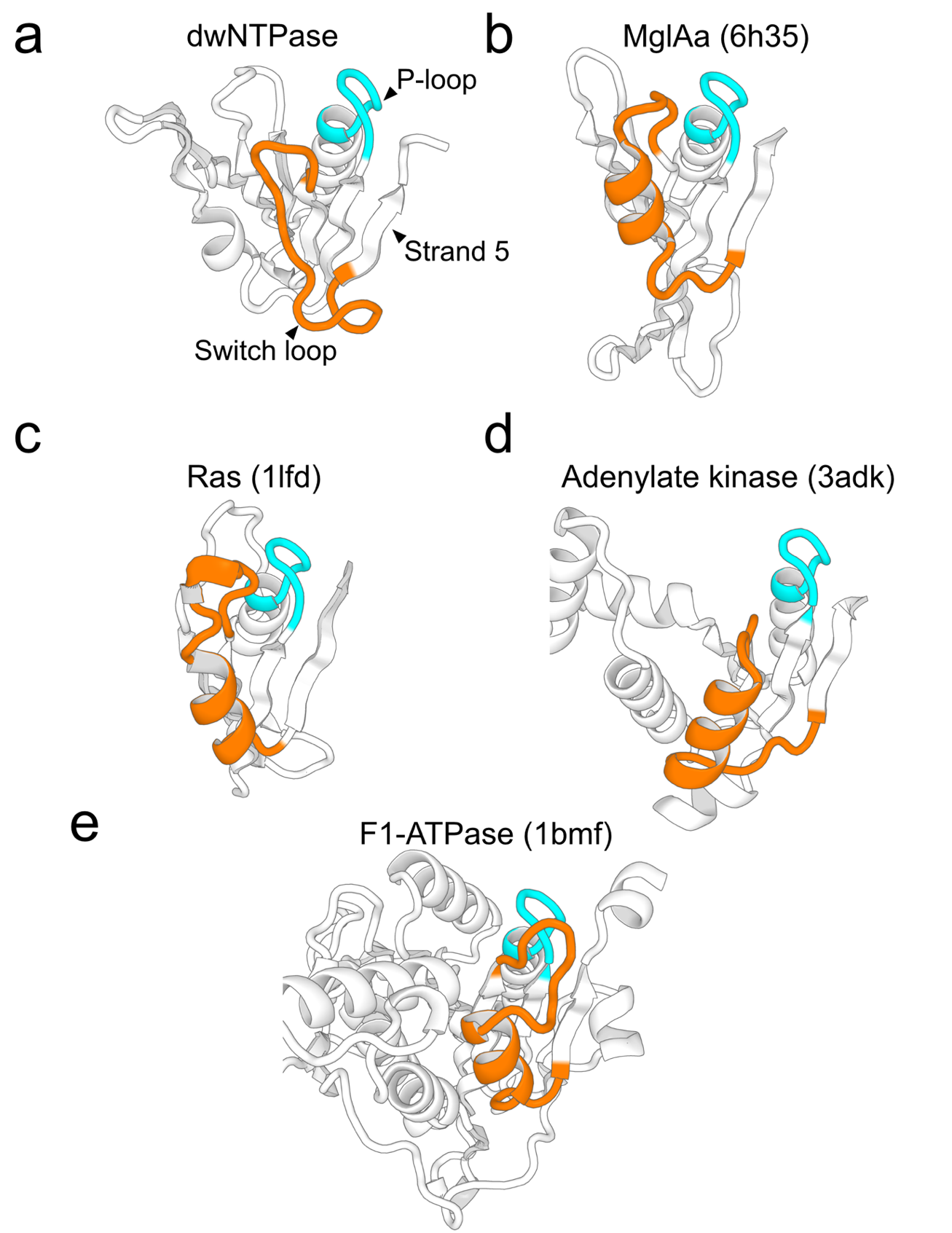
**

**Supplementary figure 4 | Comparison of the switch loop with the helical region conserved among P-loop NTPases.** Typical P-loop NTPase structures are compared to the dwNTPase P-loop domain (**a**). MglAa (**b**), Ras (**c**), adenylate-kinase (**d**) and F1-ATPase (**e**) are selected from G-proteins (SCCS: c.37.8), NKs (c.37.1) and Rec-A like protein (c.37.11) from SCOPe. The switch loop or corresponding helical regions are orange, and the P-loops are cyan. C-terminal portions of the structures that follow strand 5 are omitted for clarity. Protein names and PDB IDs are shown above the panels.

**
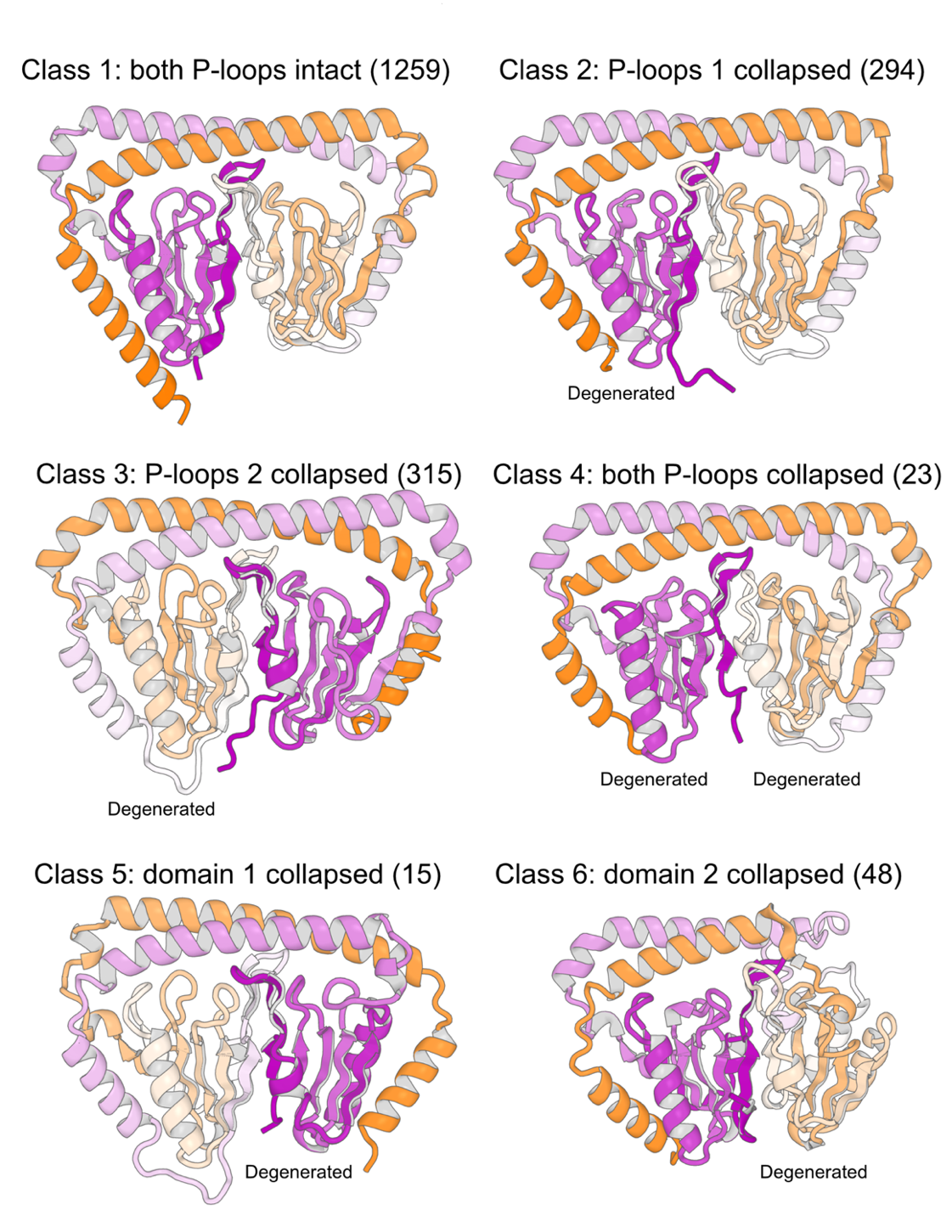
**

**Supplementary figure 5 | Variations in the dwNTPase structure.** Depending on the conservation of P-loops and domains, the dwNTPase family can be classified into six classes. The class 1 structure (e.g., Bt. dwNTPase, Uniport accession No. A0A1Y0TWD8) preserves both P-loops intact. The class 2 structure (A0A1T4Y6S3) has P-loop 1 degenerate and P-loop 2 intact. The class 3 structure (A0A1Y4J6T6) has P-loop 1 intact and P-loop 2 degenerate. The class 4 structure (A0A1I3I7D1) has both P-loops degenerate. The class 5 structure (A0A1Y4EVI7) has domain 1 degenerate and domain 2 intact. The class 6 structure (A0A101VWI5) has domain 1 intact and domain 2 degenerate. The number of structures that belong to respective classes is shown in parentheses. The structures are colored according to a purple-white-orange gradient from the N- to C-terminus. Note that the structures were rotated to show the collapsed region in front.

**
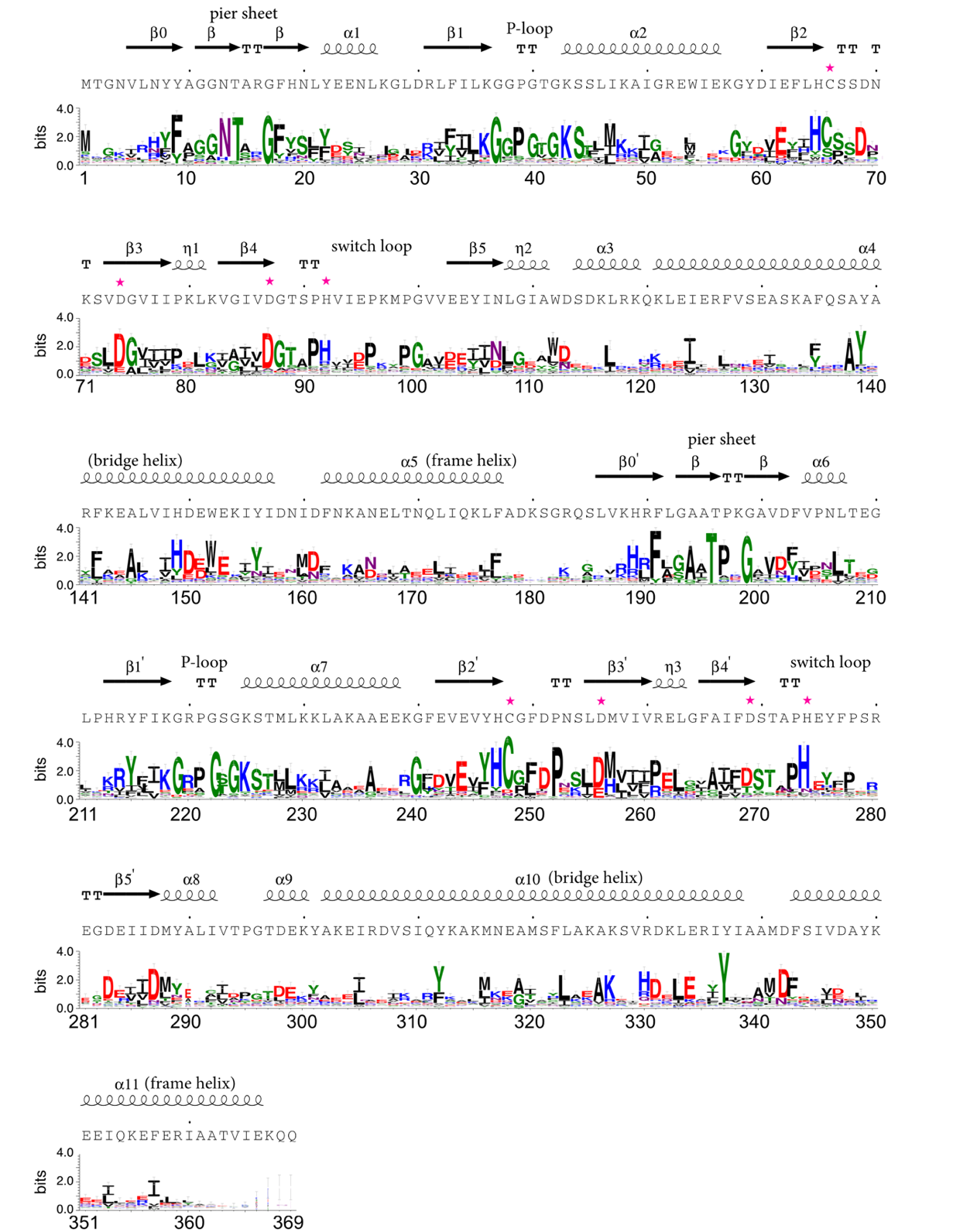
**

**Supplementary figure 6 | Sequence logo of dwNTPase.** Using HHblits, we performed a sequence search against the UniRef30 database and constructed multiple sequence alignments. Black, blue, green and red represent hydrophobic, positively charged, polar and negatively charged residues. The amino acid sequence of the representative structure (Bt. dwNTPase) and the secondary structures assigned by DSSP are shown. The vertical axis represents the bit score, and the horizontal axis represents the residue index. Putative functional residues discussed in the main text are indicated by purple stars.


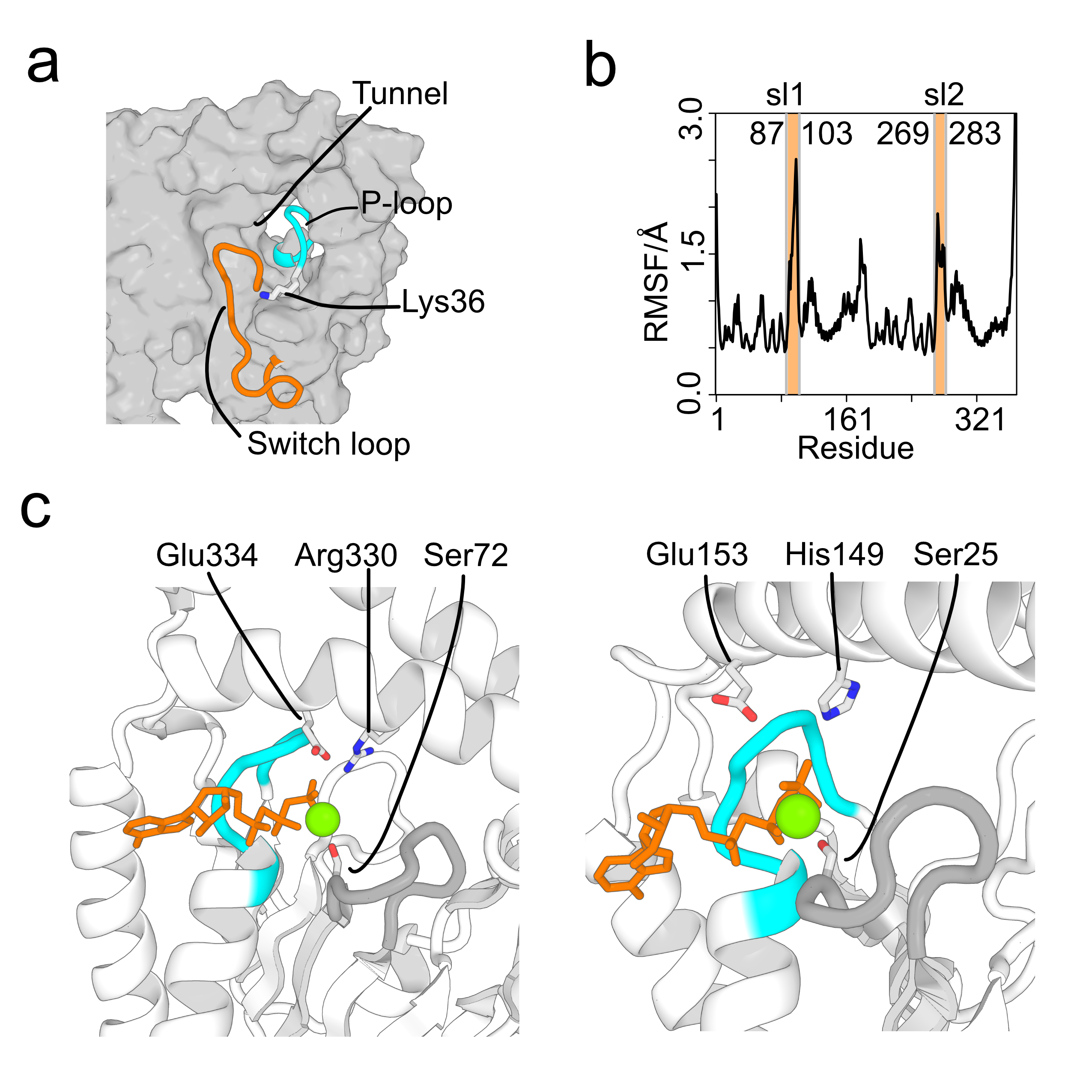


**Supplementary figure 7 | Other characteristic residues and substructures found in dwNTPases.** **a**, Location of the switch loop relative to the tunnel and the hydrogen bond formed between conserved lysine and glutamate residues. The switch loop and P-loop are shown in orange and cyan cartoon, and the rest of the structure is a grey surface. The side chain of Lys36 is shown. **b**, Root-mean-square fluctuation (RMSF) of the Cα atoms during 20 trajectories of 100 ns MD simulations. The positions of switch loops are indicated by orange bars and labels reading sl1/sl2. The numbers above the plot show the residue index of the start/end of the switch loops. **c**, Conserved residues around P-loops supporting the recognition of ATP molecules. The side chain atoms of conserved residue recognizing ATPs or Mg^2+^ ions are shown as sticks. P-loops, ATPs and Mg^2+^ ions are colored cyan, orange and green, respectively. Additional loops unobserved in other P-loop proteins are colored grey.


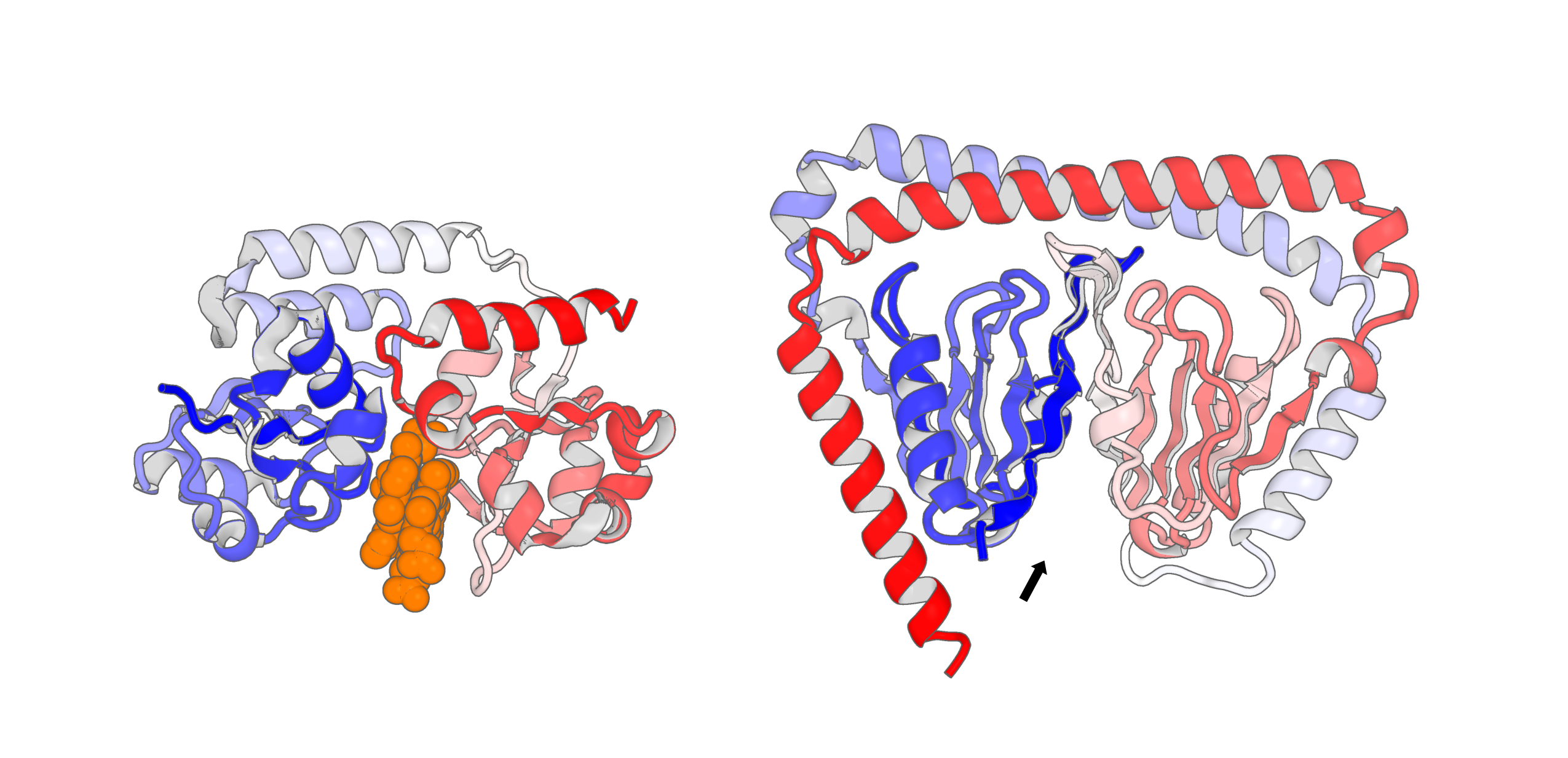


**Supplementary figure 8 | Comparison with periplasmic heme-binding proteins.** (Left) A crystal structure of periplasmic heme-binding protein HmuT (PDB ID: 3nu1) liganded with two heme molecules represented in orange CPK. (Right) The structure of dwNTPase is shown at the same scale. The cleft between two P-loop domains found in dwNTPase is indicated by an arrow. The chain is shown in cartoon representation colored according to a blue-white-red gradient from the N- to C-terminus.


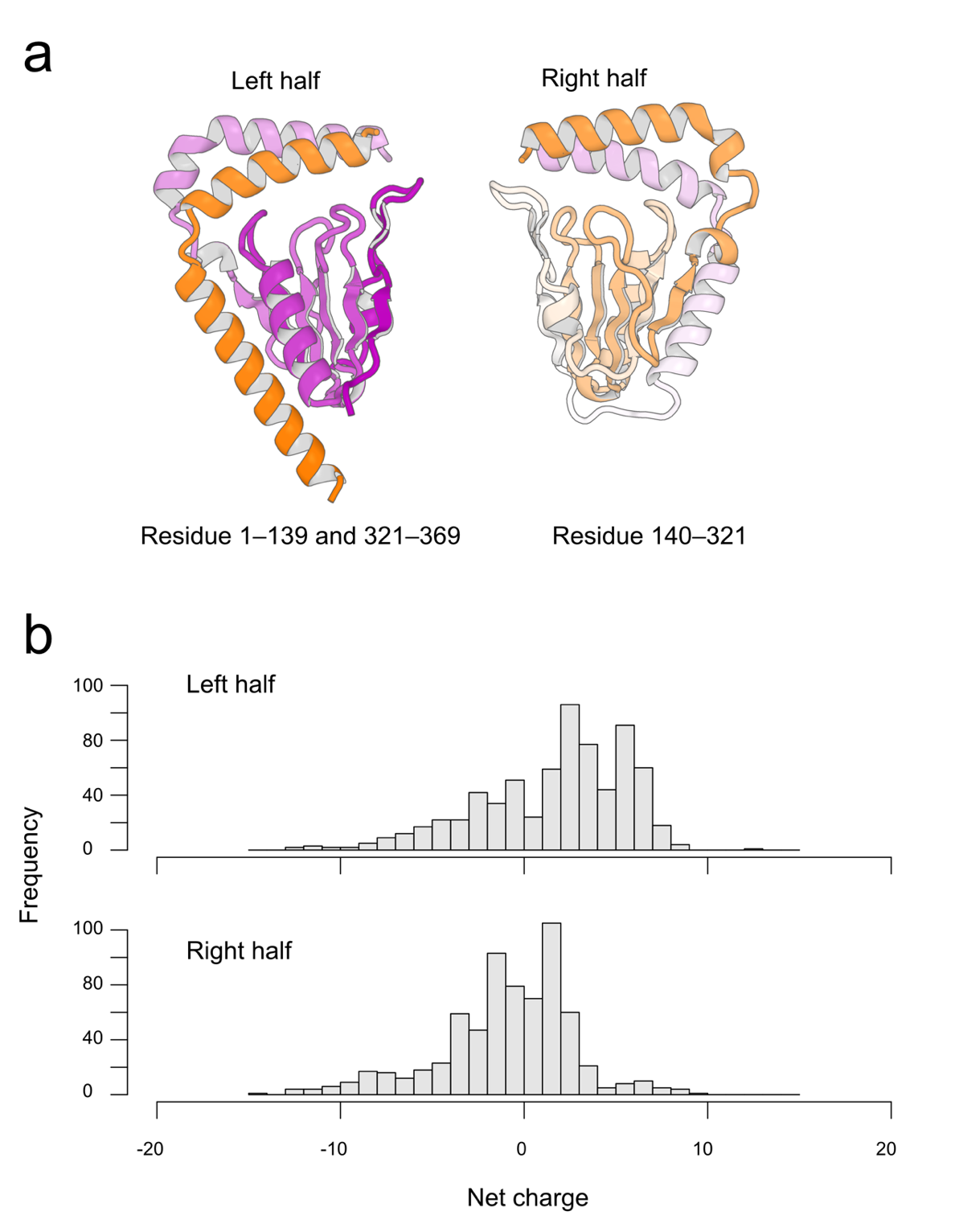


**Supplementary figure 9 | Distribution of the net charge in the left and right halves of the dwNTPase structure. a**, The representative structure was divided into left and right halves and used as a template to find corresponding halves in other structures. The structures are colored according to a purple-white-orange gradient from the N- to C-terminus. **b**, From the initially mined 711 dwNTPase structures, 707 structures with all secondary structures intact were selected. The structures were aligned to the left half of the representative structure, and the aligned regions were assigned as left halves. The remaining regions of the structures were assigned as the right halves. In calculating net charges, each Arg, His and Lys residue was counted to have a +1 charge, and each Asp and Glu residue was counted to have a –1 charge.


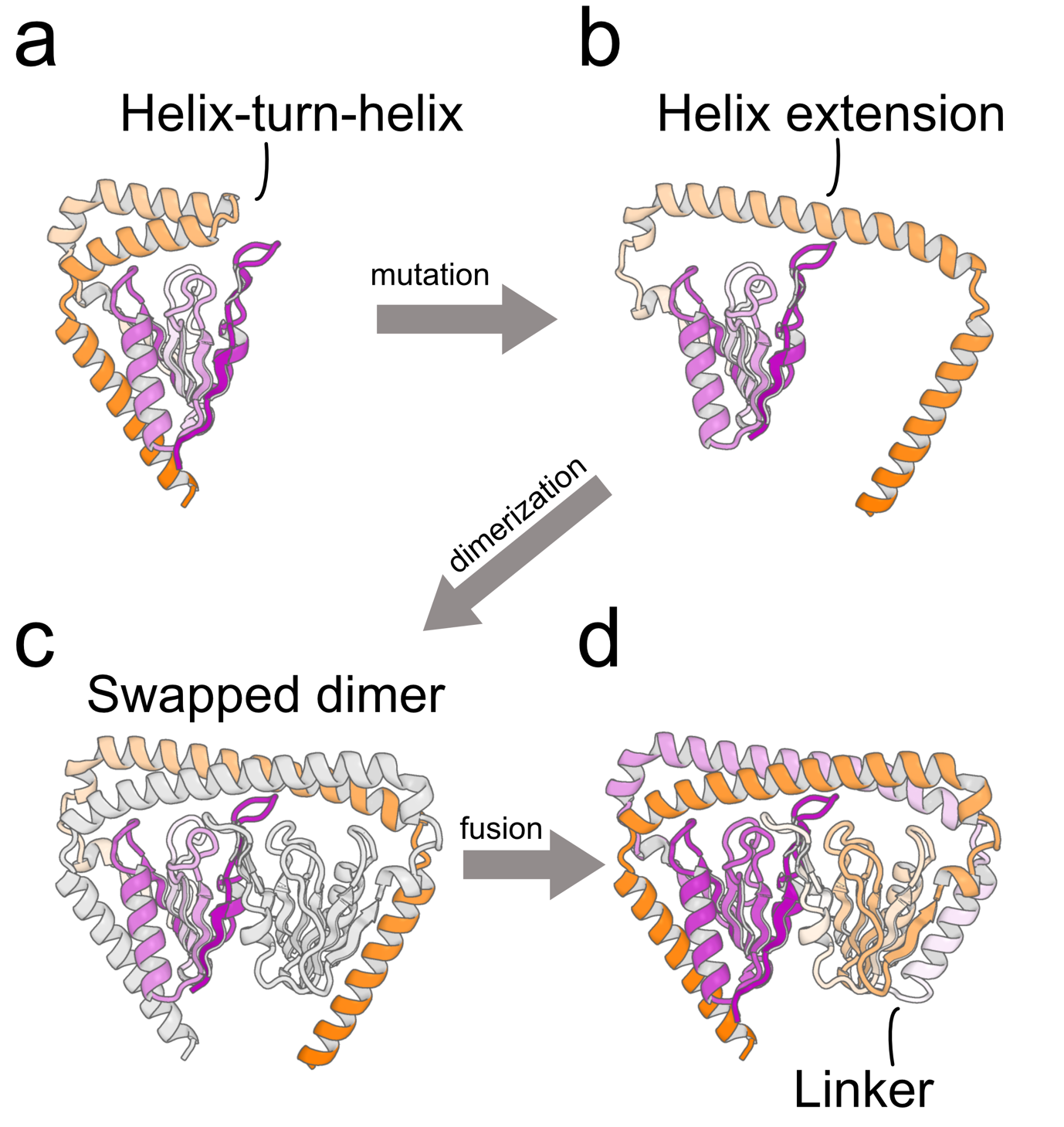


**Supplementary figure 10 | A possible evolutional trajectory to realize two-fold symmetry of dwNTPase architecture. a**, A probable ancestral protein for dwNTPase could be in a monomer form without long bridge α-helices, interrupted by a helix-turn-helix motif. The structure is colored in purple-white-orange gradient from N- to C-terminus. **b and c**, Some mutations could cause the extension of α-helix, and cause them to result in a domain-swapped dimer form. This may be coupled with duplication and divergence of the monomeric genes. One of the monomer structures is colored in purple-white-orange gradient from N- to C-terminus, and its symmetric dimer partner is colored gray. **d,** Gene fusion between the monomer genes in the domain-swapped dimer could force them into the symmetric dwNTPase architecture, where symmetric units are connected by a linker loop.
