# Supplementary material for: Dual‐wield NTPases: A novel protein family mined from AlphaFold DB
Source: Protein Sci. 2024 Mar 19;33(4):e4934. doi: 10.1002/pro.4934 (PMC10949312; doi:10.1002/pro.4934)
Supplement: Supplementary file 2 — Table S1. List of AlphaFold DB entries structurally related to dwNTPase family. First column stores the Uniprot accession codes of the protein, and the second column stores the organism or resource names. [file PRO-33-e4934-s002.docx]

**Supplementary Data Table 1:** **List of AlphaFold DB entries structurally related to dwNTPase family.** First column stores the Uniprot accession codes of the protein, and the second column stores the organism or resource names.

| A0A011VR05 | Ruminococcus albus SY3 |
| --- | --- |
| A0A023NZU8 | Bacillus bombysepticus str. Wang |
| A0A031WEG6 | Clostridioides difficile |
| A0A037ZA99 | Clostridium tetanomorphum DSM 665 |
| A0A072NEZ6 | Bacillus azotoformans MEV2011 |
| A0A072XU63 | Clostridium botulinum C/D str. BKT12695 |
| A0A072Y634 | Clostridium sp. K25 |
| A0A072YM95 | Clostridium novyi B str. NCTC 9691 |
| A0A073KAF6 | Bacillus manliponensis |
| A0A073KAV0 | Bacillus gaemokensis |
| A0A075JVS8 | Virgibacillus sp. SK37 |
| A0A075R584 | Brevibacillus laterosporus LMG 15441 |
| A0A077J9P5 | Bacillus sp. X1(2014) |
| A0A078KVF2 | [Clostridium] cellulosi |
| A0A084IXZ6 | Bacillus mycoides |
| A0A084JBV3 | Clostridium sulfidigenes |
| A0A085LC24 | Peptococcaceae bacterium SCADC1_2_3 |
| A0A089IKM2 | Paenibacillus sp. FSL H7-0737 |
| A0A089KC26 | Paenibacillus sp. FSL R7-0273 |
| A0A089KST6 | Paenibacillus sp. FSL R7-0331 |
| A0A090ITZ3 | Caldibacillus thermoamylovorans |
| A0A090YS29 | Bacillus clarus |
| A0A096B6R2 | Flavonifractor plautii 1_3_50AFAA |
| A0A096B6U9 | Flavonifractor plautii 1_3_50AFAA |
| A0A096BFL9 | Caloranaerobacter azorensis H53214 |
| A0A098AYH1 | Desulfitobacterium hafniense |
| A0A098B810 | Desulfitobacterium hafniense |
| A0A098F568 | Bacillus sp. B-jedd |
| A0A098F754 | Peribacillus simplex |
| A0A099RZW2 | Desulfosporosinus sp. HMP52 |
| A0A099S8A8 | Clostridium sp. HMP27 |
| A0A099SC38 | Clostridium sp. HMP27 |
| A0A0A0I967 | Clostridium botulinum C/D str. DC5 |
| A0A0A0IB26 | Clostridium novyi A str. 4552 |
| A0A0A0IJB9 | Clostridium haemolyticum NCTC 8350 |
| A0A0A0IUT0 | Clostridium novyi A str. 4570 |
| A0A0A1MWX8 | Oceanobacillus oncorhynchi |
| A0A0A2TJ00 | Desulfosporosinus sp. Tol-M |
| A0A0A2TS74 | Desulfosporosinus sp. Tol-M |
| A0A0A2VHB7 | Pontibacillus chungwhensis BH030062 |
| A0A0A3I689 | Lysinibacillus manganicus DSM 26584 |
| A0A0A3IIX0 | Lysinibacillus boronitolerans JCM 21713 = 10a = NBRC 103108 |
| A0A0A3IM15 | Lysinibacillus sinduriensis BLB-1 = JCM 15800 |
| A0A0A3J5T7 | Lysinibacillus massiliensis 4400831 = CIP 108448 = CCUG 49529 |
| A0A0A5GE29 | Pontibacillus marinus BH030004 = DSM 16465 |
| A0A0A7FXV3 | Clostridium baratii str. Sullivan |
| A0A0A8JGW3 | Bacillus sp. (strain OxB-1) |
| A0A0A8X6A0 | Bacillus selenatarsenatis SF-1 |
| A0A0B0HMM4 | Paenibacillus sp. P1XP2 |
| A0A0B0HQQ8 | Paenibacillus sp. P1XP2 |
| A0A0B0IK09 | Alkalihalobacillus okhensis |
| A0A0B1Y1A2 | Lysinibacillus sp. A1 |
| A0A0B3VZH0 | Terrisporobacter othiniensis |
| A0A0B4WCA3 | Clostridium botulinum Prevot_594 |
| A0A0B5X8Z0 | Bacillus thuringiensis |
| A0A0B7MFU2 | Syntrophaceticus schinkii |
| A0A0B7MIF4 | Syntrophaceticus schinkii |
| A0A0B7MJC7 | Syntrophaceticus schinkii |
| A0A0C1UFI3 | Clostridium argentinense CDC 2741 |
| A0A0C2RR63 | Jeotgalibacillus campisalis |
| A0A0C2TRM5 | Bacillus badius |
| A0A0C2UQT9 | Cohnella kolymensis |
| A0A0C2VZ26 | Jeotgalibacillus soli |
| A0A0C3HBH1 | Clostridium botulinum |
| A0A0C5BZN8 | Weizmannia coagulans |
| A0A0C7GBA6 | Paeniclostridium sordellii |
| A0A0C7N6A9 | Moorella glycerini |
| A0A0C7NLI7 | Moorella glycerini |
| A0A0C7NNC1 | Moorella glycerini |
| A0A0D0EGA2 | Caldibacillus thermoamylovorans |
| A0A0D0FPH4 | Caldibacillus thermoamylovorans |
| A0A0D0QXF9 | Bacillus sp. L_1B0_5 |
| A0A0D1BPS2 | Clostridium botulinum B2 450 |
| A0A0D1R4D3 | Bacillus thuringiensis Sbt003 |
| A0A0D3VI41 | Paenibacillus sp. IHBB 10380 |
| A0A0D7WTV6 | Paenibacillus terrae |
| A0A0D8I5Y8 | Clostridium aceticum |
| A0A0D8J352 | Ruthenibacterium lactatiformans |
| A0A0E1L182 | Clostridium botulinum CDC_1436 |
| A0A0E1MKX4 | Bacillus cereus |
| A0A0E3JRD0 | Clostridium scatologenes |
| A0A0E3W385 | Syntrophomonas zehnderi OL-4 |
| A0A0E4GWT5 | Syntrophomonas zehnderi OL-4 |
| A0A0F2JK31 | Desulfosporosinus sp. I2 |
| A0A0F2NB27 | Peptococcaceae bacterium BRH_c4a |
| A0A0F2PJY4 | Peptococcaceae bacterium BRH_c8a |
| A0A0F2PRR1 | Peptococcaceae bacterium BRH_c4b |
| A0A0F2Q015 | Clostridiaceae bacterium BRH_c20a |
| A0A0F2S8L7 | Peptococcaceae bacterium BRH_c23 |
| A0A0F2SMQ4 | Peptococcaceae bacterium BRH_c23 |
| A0A0F3FS45 | Clostridium baratii |
| A0A0F5I727 | Quasibacillus thermotolerans |
| A0A0F5RKY0 | Bacillus sp. UMTAT18 |
| A0A0F6G0J2 | Bacillus thuringiensis subsp. kurstaki |
| A0A0F6Y0I8 | Brevibacillus laterosporus |
| A0A0F7RJU5 | Bacillus anthracis |
| A0A0G8C3G0 | Bacillus wiedmannii |
| A0A0G8E5A0 | Bacillus cereus |
| A0A0G8F8F2 | Bacillus cereus |
| A0A0G9LEG5 | Clostridium sp. C8 |
| A0A0H2YUK9 | Clostridium perfringens (strain ATCC 13124 / DSM 756 / JCM 1290 / NCIMB 6125 / NCTC 8237 / Type A) |
| A0A0H3NFU8 | Clostridioides difficile (strain CD196) |
| A0A0H5SV22 | Herbinix hemicellulosilytica |
| A0A0J1DLS7 | Peptococcaceae bacterium 1109 |
| A0A0J1FUB6 | Desulfosporosinus acididurans |
| A0A0J1HRD9 | Bacillus anthracis |
| A0A0J1IJI5 | Desulfosporosinus acididurans |
| A0A0J5GZ52 | Bacillus sp. LL01 |
| A0A0J5VYY5 | Cytobacillus firmus |
| A0A0J6L130 | Bacillus sp. LK2 |
| A0A0J6Z9S3 | Bacillus cereus |
| A0A0J7DES4 | Bacillus cereus |
| A0A0J7GWC3 | Bacillus cereus |
| A0A0J8D4K4 | Clostridium cylindrosporum DSM 605 |
| A0A0K0GAZ8 | Bacilli bacterium VT-13-104 |
| A0A0K0Q6V2 | Bacillus thuringiensis |
| A0A0K8J594 | Herbinix luporum |
| A0A0K9F4F4 | Lysinibacillus xylanilyticus |
| A0A0K9GZ84 | Peribacillus loiseleuriae |
| A0A0K9H9Y9 | Bacillus sp. FJAT-27231 |
| A0A0K9MEL7 | Bacillus sp. FJAT-27238 |
| A0A0K9YXI0 | Brevibacillus reuszeri |
| A0A0L0WD40 | Gottschalkia purinilytica |
| A0A0L6JUT0 | Pseudobacteroides cellulosolvens ATCC 35603 = DSM 2933 |
| A0A0L6VZ10 | Thermincola ferriacetica |
| A0A0L6ZAH9 | Clostridium homopropionicum DSM 5847 |
| A0A0L7NS99 | Clostridium botulinum |
| A0A0L9YAX5 | Clostridium botulinum |
| A0A0M0G936 | Sporosarcina globispora |
| A0A0M0GPI9 | Bacillus marisflavi |
| A0A0M0LLZ8 | Viridibacillus arvi |
| A0A0M0WCR2 | Bacillus sp. FJAT-21945 |
| A0A0M0WVJ1 | Lysinibacillus sp. FJAT-14745 |
| A0A0M1IYQ9 | Clostridium sp. L74 |
| A0A0M1N1C9 | Paenibacillus solani |
| A0A0M1NSP8 | Bacillus sp. FJAT-22058 |
| A0A0M1URX7 | Paeniclostridium sordellii |
| A0A0M2PCR3 | Bacillus sp. SA1-12 |
| A0A0M2SZL0 | Mesobacillus campisalis |
| A0A0M2U5Z7 | Clostridiales bacterium PH28_bin88 |
| A0A0M2VWH4 | Paenibacillus sp. DMB20 |
| A0A0M3DJ91 | Paraclostridium benzoelyticum |
| A0A0M4G2T7 | Bacillus sp. FJAT-18017 |
| A0A0M4GHP4 | Bacillus sp. FJAT-22090 |
| A0A0M8PRN4 | Lysinibacillus sp. FJAT-14222 |
| A0A0M9GSA4 | Bacillus sp. CHD6a |
| A0A0M9WYR3 | Lysinibacillus contaminans |
| A0A0N0CX44 | Lysinibacillus macroides |
| A0A0N0M8S6 | Oceanobacillus caeni |
| A0A0N1HD10 | Clostridioides difficile |
| A0A0P6VY21 | Rossellomorea vietnamensis |
| A0A0P7JZU5 | Lysinibacillus sp. ZYM-1 |
| A0A0P8VTG0 | Caloranaerobacter sp. TR13 |
| A0A0Q0R9D7 | Bacillus thuringiensis |
| A0A0Q3QUY6 | Cytobacillus solani |
| A0A0Q3S4L7 | Psychrobacillus sp. FJAT-21963 |
| A0A0Q3T811 | Brevibacillus choshinensis |
| A0A0Q3W0U6 | Bacillus sp. FJAT-25509 |
| A0A0Q3WSD7 | Bacillus shackletonii |
| A0A0Q6KXT2 | Bacillus sp. Leaf406 |
| A0A0Q9VYZ9 | Bacillus sp. Soil768D1 |
| A0A0Q9Y4M1 | Virgibacillus soli |
| A0A0R3K1I6 | Caloramator mitchellensis |
| A0A0S2W210 | Intestinimonas butyriciproducens |
| A0A0S6U621 | Clostridium botulinum B str. Osaka05 |
| A0A0S6U9Q5 | Moorella thermoacetica Y72 |
| A0A0S6UH05 | Moorella thermoacetica Y72 |
| A0A0U1KNY7 | Paraliobacillus sp. PM-2 |
| A0A0U1L3G4 | Sporomusa ovata |
| A0A0U1NX13 | Neobacillus massiliamazoniensis |
| A0A0U2UAW9 | Paenibacillus naphthalenovorans |
| A0A0U2UQH7 | Paenibacillus sp. 32O-W |
| A0A0U3WB32 | Lentibacillus amyloliquefaciens |
| A0A0U9H622 | Oceanobacillus picturae |
| A0A0V8HJY0 | Bacillus enclensis |
| A0A0W1AXZ5 | Paenibacillus etheri |
| A0A0W1JHD8 | Desulfitobacterium hafniense |
| A0A0W1JPB7 | Desulfitobacterium hafniense |
| A0A0W7TVM7 | Ruthenibacterium lactatiformans |
| A0A0W7YLD2 | Lysinibacillus sp. F5 |
| A0A0W8E4K5 | hydrocarbon metagenome |
| A0A0W8E642 | hydrocarbon metagenome |
| A0A0X8D2G5 | Aneurinibacillus sp. XH2 |
| A0A0X8G2A8 | Turicibacter sp. H121 |
| A0A101F7Q9 | Thermoanaerobacterales bacterium 50_218 |
| A0A101FQZ0 | Clostridia bacterium 62_21 |
| A0A101FR57 | Clostridia bacterium 62_21 |
| A0A101GVR0 | Desulfotomaculum sp. 46_80 |
| A0A101HT37 | Pelotomaculum thermopropionicum |
| A0A101V9B3 | Desulfitibacter sp. BRH_c19 |
| A0A101VTK5 | Gracilibacter sp. BRH_c7a |
| A0A101VWI5 | Gracilibacter sp. BRH_c7a |
| A0A101WDA9 | Desulfosporosinus sp. BRH_c37 |
| A0A101Y1Q0 | Paenibacillus sp. DMB5 |
| A0A109G0D1 | Bacillus mycoides |
| A0A117KXD0 | Clostridia bacterium 41_269 |
| A0A117S2R7 | Desulfitibacter sp. BRH_c19 |
| A0A120GQ95 | Peribacillus simplex |
| A0A125YDJ8 | Clostridioides difficile ATCC 9689 = DSM 1296 |
| A0A127DDQ3 | Peribacillus simplex |
| A0A127EJE1 | Clostridium perfringens |
| A0A127W314 | Sporosarcina psychrophila |
| A0A133KH74 | Weizmannia coagulans |
| A0A133MV32 | Clostridium perfringens |
| A0A135L4N3 | Tepidibacillus decaturensis |
| A0A135WG74 | Sporosarcina sp. HYO08 |
| A0A136BHT8 | Bacillus cereus |
| A0A140L102 | Fervidicola ferrireducens |
| A0A140LB51 | Thermotalea metallivorans |
| A0A143HCD7 | Rummeliibacillus stabekisii |
| A0A143ZUA6 | Eubacteriaceae bacterium CHKCI005 |
| A0A150BFB0 | Bacillus cereus |
| A0A150BLI9 | Bacillus cereus |
| A0A150D114 | Bacillus cereus |
| A0A150E2M5 | Bacillus cereus |
| A0A150EXW3 | Bacillus cereus |
| A0A150FRV8 | [Clostridium] paradoxum JW-YL-7 = DSM 7308 |
| A0A150JV47 | Weizmannia coagulans |
| A0A150JY23 | Weizmannia coagulans |
| A0A150KAW3 | Weizmannia coagulans |
| A0A150L6D1 | Bacillus sporothermodurans |
| A0A151AP40 | Clostridium colicanis DSM 13634 |
| A0A151AXY0 | Moorella mulderi DSM 14980 |
| A0A151AZJ8 | Moorella mulderi DSM 14980 |
| A0A151B040 | Clostridium tepidiprofundi DSM 19306 |
| A0A151UTC6 | Bacillus cereus |
| A0A154BVE5 | Anaerosporomusa subterranea |
| A0A158RNF5 | Bacillus cereus (strain 03BB102) |
| A0A160H875 | Bacillus cereus |
| A0A160IK67 | Fictibacillus phosphorivorans |
| A0A160MHH3 | Bacillus oceanisediminis 2691 |
| A0A161QQ05 | Bacillus cereus |
| A0A161RGG3 | Bhargavaea cecembensis |
| A0A161XFK1 | Clostridium magnum DSM 2767 |
| A0A162M8B1 | Thermovenabulum gondwanense |
| A0A162U197 | Clostridium magnum DSM 2767 |
| A0A163QB21 | Fictibacillus phosphorivorans |
| A0A164MFU9 | Bacillus cereus |
| A0A165J5J0 | Bacillus marisflavi |
| A0A167ASF5 | Paenibacillus crassostreae |
| A0A168DKC2 | Paenibacillus macquariensis |
| A0A168KZD2 | Paenibacillus antarcticus |
| A0A169ZHB1 | Paenibacillus glacialis |
| A0A173QZX0 | Turicibacter sanguinis |
| A0A173RAA6 | Faecalibacterium prausnitzii |
| A0A173WID7 | Turicibacter sanguinis |
| A0A173WRV5 | Clostridium ventriculi |
| A0A173Z3K4 | Clostridium disporicum |
| A0A174BH00 | Faecalibacterium prausnitzii |
| A0A174FC78 | Clostridium paraputrificum |
| A0A174GRL5 | Clostridium disporicum |
| A0A174MRJ7 | Anaerotruncus colihominis |
| A0A174NG55 | Anaerotruncus colihominis |
| A0A174SS44 | Clostridium baratii |
| A0A174VYZ3 | Flavonifractor plautii |
| A0A174WPP5 | Flavonifractor plautii |
| A0A175LR66 | Clostridium botulinum B2 433 |
| A0A177KLS5 | Domibacillus aminovorans |
| A0A177KZN5 | Domibacillus aminovorans |
| A0A177XSF6 | Brevibacillus sp. SKDU10 |
| A0A177ZJ27 | Lederbergia galactosidilyticus |
| A0A179T4F9 | Metabacillus litoralis |
| A0A193CMG2 | Bacillus thuringiensis serovar coreanensis |
| A0A1A5X1Q3 | Brevibacillus sp. WF146 |
| A0A1A5YUR3 | Paenibacillus oryzae |
| A0A1B1KZS3 | Bacillus thuringiensis |
| A0A1B1YDN0 | Thermoclostridium stercorarium subsp. thermolacticum DSM 2910 |
| A0A1B1YKT7 | Thermoclostridium stercorarium subsp. leptospartum DSM 9219 |
| A0A1B1YZX8 | Fictibacillus arsenicus |
| A0A1B2E1A4 | Paenibacillus ihbetae |
| A0A1B3XVP1 | Peribacillus muralis |
| A0A1B7LBR9 | Desulfotomaculum copahuensis |
| A0A1B7LND2 | Candidatus Arthromitus sp. SFB-turkey |
| A0A1B8WEU6 | Bacillus sp. FJAT-27264 |
| A0A1B8WIC8 | Bacillus sp. FJAT-26390 |
| A0A1B9AER0 | Bacillus sp. FJAT-27225 |
| A0A1B9AYB3 | Bacillus wudalianchiensis |
| A0A1C0AAB8 | Orenia metallireducens |
| A0A1C2XR73 | Dehalobacter sp. TeCB1 |
| A0A1C2Y2N9 | Dehalobacter sp. TeCB1 |
| A0A1C3F700 | Desulfosporosinus sp. BG |
| A0A1C3T2Z1 | Bacillus mycoides |
| A0A1C3ZGP9 | Bacillus cereus |
| A0A1C3ZK95 | Bacillus mycoides |
| A0A1C3ZL92 | Bacillus wiedmannii |
| A0A1C3ZNB1 | Bacillus thuringiensis |
| A0A1C5L3M5 | uncultured Ruminococcus sp |
| A0A1C5LVQ8 | uncultured Faecalibacterium sp |
| A0A1C5PDC5 | uncultured Oscillibacter sp |
| A0A1C5PER6 | uncultured Clostridium sp |
| A0A1C5PYK2 | uncultured Faecalibacterium sp |
| A0A1C5SL51 | uncultured Eubacterium sp |
| A0A1C5URW7 | uncultured Clostridium sp |
| A0A1C5W3J4 | uncultured Ruminococcus sp |
| A0A1C5WEL9 | uncultured Clostridium sp |
| A0A1C6AI66 | uncultured Flavonifractor sp |
| A0A1C6AIX5 | uncultured Flavonifractor sp |
| A0A1C6BAZ6 | uncultured Flavonifractor sp |
| A0A1C6BC28 | uncultured Flavonifractor sp |
| A0A1C6BD87 | uncultured Ruminococcus sp |
| A0A1C6BJ78 | uncultured Clostridium sp |
| A0A1C6BPE7 | uncultured Flavonifractor sp |
| A0A1C6C6A7 | uncultured Ruminococcus sp |
| A0A1C6C7X7 | uncultured Clostridium sp |
| A0A1C6C8D7 | uncultured Ruminococcus sp |
| A0A1C6CCN1 | uncultured Clostridium sp |
| A0A1C6D0U7 | uncultured Ruminococcus sp |
| A0A1C6F277 | uncultured Clostridium sp |
| A0A1C6FK51 | uncultured Clostridium sp |
| A0A1C6FLL0 | uncultured Anaerotruncus sp |
| A0A1C6FLY0 | uncultured Clostridium sp |
| A0A1C6H0S5 | uncultured Flavonifractor sp |
| A0A1C6HFU5 | uncultured Flavonifractor sp |
| A0A1C6HG51 | uncultured Flavonifractor sp |
| A0A1C6I316 | uncultured Ruminococcus sp |
| A0A1C6IIH8 | uncultured Oscillibacter sp |
| A0A1C6JLW8 | uncultured Eubacterium sp |
| A0A1C6W9W4 | Bacillus wiedmannii |
| A0A1C7FJT2 | Flavonifractor plautii |
| A0A1C9BMK6 | Bacillus thuringiensis Bt18247 |
| A0A1D3N109 | Bacillus mycoides |
| A0A1D3QAF9 | Bacillus cereus |
| A0A1D7XAR7 | Moorella thermoacetica |
| A0A1D7XC76 | Moorella thermoacetica |
| A0A1D7XN80 | Clostridium taeniosporum |
| A0A1D8GCD5 | Geosporobacter ferrireducens |
| A0A1D8GCE3 | Geosporobacter ferrireducens |
| A0A1D8JJK0 | Sporosarcina ureilytica |
| A0A1D9FQP5 | Clostridium formicaceticum |
| A0A1E3BXQ2 | Clostridium sp. Bc-iso-3 |
| A0A1E4LH44 | Clostridium sp. SCN 57-10 |
| A0A1E4R2F3 | Lysinibacillus fusiformis |
| A0A1E5G5R6 | Desulfuribacillus alkaliarsenatis |
| A0A1E5K3E5 | Oceanobacillus sp. E9 |
| A0A1E8BDF3 | Bacillus mycoides |
| A0A1E8BV13 | Bacillus mycoides |
| A0A1E8F1G9 | Clostridium acetireducens DSM 10703 |
| A0A1F8U479 | Clostridiales bacterium GWB2_37_7 |
| A0A1F8UM10 | Clostridiales bacterium GWD2_32_59 |
| A0A1G4EEX6 | Bacillus mycoides |
| A0A1G5C0U1 | Alkaliphilus peptidifermentans DSM 18978 |
| A0A1G5HY52 | Desulfoluna spongiiphila |
| A0A1G6DDP0 | Ruminococcaceae bacterium FB2012 |
| A0A1G6KYH8 | Pelagirhabdus alkalitolerans |
| A0A1G7DNX2 | Bhargavaea beijingensis |
| A0A1G7MCD1 | Sporolituus thermophilus DSM 23256 |
| A0A1G7TAP9 | Fontibacillus panacisegetis |
| A0A1G7USJ3 | Desulfosporosinus hippei DSM 8344 |
| A0A1G7Y1D2 | Aneurinibacillus thermoaerophilus |
| A0A1G8GQA3 | Alteribacillus persepolensis |
| A0A1G8MRA4 | Alteribacillus bidgolensis |
| A0A1G8RPF8 | Natribacillus halophilus |
| A0A1G9AD17 | Paenibacillus typhae |
| A0A1G9BBN1 | Natronincola ferrireducens |
| A0A1G9FM54 | Clostridium cochlearium |
| A0A1G9RMI9 | Romboutsia lituseburensis DSM 797 |
| A0A1G9WXA1 | Tenuibacillus multivorans |
| A0A1G9XUL1 | Bacillus sp. OK048 |
| A0A1H0A0L6 | Psychrobacillus sp. OK028 |
| A0A1H0AAX9 | Acetanaerobacterium elongatum |
| A0A1H0Q2G8 | Clostridium gasigenes |
| A0A1H0TBK8 | Litchfieldia salsus |
| A0A1H0ZBE8 | Virgibacillus salinus |
| A0A1H2T661 | Tepidimicrobium xylanilyticum |
| A0A1H3QW75 | Bacillus sp. 166amftsu |
| A0A1H3RSI3 | Proteiniborus ethanoligenes |
| A0A1H3V0B1 | Evansella caseinilytica |
| A0A1H3XM84 | Thalassobacillus cyri |
| A0A1H5TB50 | Caloramator fervidus |
| A0A1H6B9T0 | Bacillus sp. ok061 |
| A0A1H6TDH0 | Bhargavaea ginsengi |
| A0A1H7MK05 | Ruminococcus albus |
| A0A1H7N6W8 | Paenibacillus sp. cl141a |
| A0A1H7YNT9 | Hydrogenoanaerobacterium saccharovorans |
| A0A1H7YQC1 | Hydrogenoanaerobacterium saccharovorans |
| A0A1H8BB33 | Candidatus Frackibacter sp. WG12 |
| A0A1H8GM21 | Mesobacillus persicus |
| A0A1H9E2K8 | Piscibacillus halophilus |
| A0A1H9HI04 | Lysinibacillus fusiformis |
| A0A1H9TG16 | Salipaludibacillus aurantiacus |
| A0A1H9TZI7 | Psychrobacillus sp. OK032 |
| A0A1I0BNV3 | Oceanobacillus limi |
| A0A1I0CFN1 | Anaerobranca gottschalkii DSM 13577 |
| A0A1I0CQ85 | Salinibacillus kushneri |
| A0A1I0DKG8 | [Clostridium] polysaccharolyticum |
| A0A1I0EAX8 | Natronincola peptidivorans |
| A0A1I0WGY8 | Lentibacillus halodurans |
| A0A1I0ZY24 | Clostridium frigidicarnis |
| A0A1I1H869 | Bacillus sp. OV322 |
| A0A1I1PB18 | Clostridium uliginosum |
| A0A1I1PZW9 | Ruminococcus albus |
| A0A1I1Q201 | Bacillus sp. 491mf |
| A0A1I1UFT1 | Bacillus sp. OV194 |
| A0A1I1WPY2 | Lentibacillus persicus |
| A0A1I2CR33 | Alteribacillus iranensis |
| A0A1I2EJD4 | Bacillus sp. OV194 |
| A0A1I2N521 | Desulfotomaculum arcticum DSM 17038 |
| A0A1I3I7D1 | Ruminococcaceae bacterium D5 |
| A0A1I3L7E6 | Brevibacillus centrosporus |
| A0A1I3S7X9 | Terrisporobacter glycolicus |
| A0A1I4LC20 | Bacillus sp. 5mfcol3.1 |
| A0A1I4PFL3 | Paenibacillus sp. 1_12 |
| A0A1I5HXL5 | Anaerocolumna aminovalerica |
| A0A1I5ML01 | Oscillibacter sp. PC13 |
| A0A1I5XIS3 | Psychrobacillus psychrotolerans |
| A0A1I5Y3L6 | Caldicoprobacter faecalis |
| A0A1I6C6U5 | Bacillus sp. cl95 |
| A0A1I6DPK0 | Desulfallas geothermicus DSM 3669 |
| A0A1I6SB48 | Halolactibacillus miurensis |
| A0A1I6W0X2 | Bacillus sp. 103mf |
| A0A1I7JA14 | Clostridium sp. DSM 8431 |
| A0A1J1D1X8 | Clostridium sporogenes |
| A0A1J5NNT6 | Moorella thermoacetica |
| A0A1J5NQY3 | Moorella thermoacetica |
| A0A1J6WUG5 | Bacillus aquimaris |
| A0A1J9UTK0 | Bacillus albus |
| A0A1J9VXU3 | Bacillus paramycoides |
| A0A1J9WCS4 | Bacillus anthracis |
| A0A1J9XDM1 | Bacillus cereus |
| A0A1K1NBX6 | Ruminococcus sp. YE71 |
| A0A1K1NXA5 | Paenibacillus sp. UNCCL117 |
| A0A1L3MR54 | Bacillus weihaiensis |
| A0A1L3NEL7 | Clostridium sporogenes |
| A0A1M2UH20 | Bacillus cereus |
| A0A1M2ZNC5 | Clostridiales bacterium 43-6 |
| A0A1M4M7T2 | Proteiniborus sp. DW1 |
| A0A1M4NBF5 | Clostridium sp. N3C |
| A0A1M4S8A9 | Tissierella praeacuta DSM 18095 |
| A0A1M4VAW7 | Caloramator proteoclasticus DSM 10124 |
| A0A1M4XQW6 | Clostridium fallax |
| A0A1M4ZT88 | Desulfotomaculum putei DSM 12395 |
| A0A1M5BA76 | Caloramator proteoclasticus DSM 10124 |
| A0A1M5BNT5 | Desulfofundulus australicus DSM 11792 |
| A0A1M5CIN4 | Caldanaerobius fijiensis DSM 17918 |
| A0A1M5FEF6 | Ornithinibacillus halophilus |
| A0A1M5KLM9 | Thermosyntropha lipolytica DSM 11003 |
| A0A1M5R855 | Desulfosporosinus lacus DSM 15449 |
| A0A1M5RDV9 | Asaccharospora irregularis DSM 2635 |
| A0A1M5RTW9 | Thermosyntropha lipolytica DSM 11003 |
| A0A1M5T741 | Tepidibacter thalassicus DSM 15285 |
| A0A1M5UUA6 | Sporanaerobacter acetigenes DSM 13106 |
| A0A1M5VU38 | Caloranaerobacter azorensis DSM 13643 |
| A0A1M5W4Z4 | Desulfosporosinus lacus DSM 15449 |
| A0A1M5XVW7 | Clostridium collagenovorans DSM 3089 |
| A0A1M6E8G4 | Clostridium intestinale DSM 6191 |
| A0A1M6EWW6 | Lutispora thermophila DSM 19022 |
| A0A1M6JJI5 | Clostridium amylolyticum |
| A0A1M6JJZ0 | Desulfofundulus thermosubterraneus DSM 16057 |
| A0A1M6KEF1 | Thermoclostridium caenicola |
| A0A1M6LSB9 | Caminicella sporogenes DSM 14501 |
| A0A1M6M5J8 | Paramaledivibacter caminithermalis DSM 15212 |
| A0A1M6MFZ1 | Tepidibacter formicigenes DSM 15518 |
| A0A1M6MLV0 | Anaerobranca californiensis DSM 14826 |
| A0A1M6N6A3 | Geosporobacter subterraneus DSM 17957 |
| A0A1M6Q1U2 | Geosporobacter subterraneus DSM 17957 |
| A0A1M6QUV4 | Clostridium cavendishii DSM 21758 |
| A0A1M6RTD8 | Hathewaya proteolytica DSM 3090 |
| A0A1M6UIB8 | Desulfotomaculum aeronauticum DSM 10349 |
| A0A1M7HWB5 | Anaerosporobacter mobilis DSM 15930 |
| A0A1M7LCH2 | Caldanaerovirga acetigignens |
| A0A1M7TBT0 | Desulfitobacterium chlororespirans DSM 11544 |
| A0A1M7UMW6 | Desulfitobacterium chlororespirans DSM 11544 |
| A0A1N7ADS6 | Peribacillus simplex |
| A0A1N7CX82 | Paenibacillus macquariensis |
| A0A1N7E780 | Bacillus cereus |
| A0A1Q5P5M7 | Domibacillus mangrovi |
| A0A1Q6KNQ7 | Clostridiales bacterium 52_15 |
| A0A1Q6QCJ8 | Firmicutes bacterium CAG:129_59_24 |
| A0A1Q6RC60 | Firmicutes bacterium CAG:24053_14 |
| A0A1Q6RFB9 | Oscillibacter sp. 57_20 |
| A0A1Q6RFH2 | Oscillibacter sp. 57_20 |
| A0A1Q6SIP3 | Firmicutes bacterium CAG:272_52_7 |
| A0A1Q6TI18 | Ruminococcus sp. 37_24 |
| A0A1Q8Q1G0 | Domibacillus antri |
| A0A1Q8R323 | Desulfosporosinus sp. OL |
| A0A1Q8UX18 | Alkalihalobacillus pseudofirmus |
| A0A1Q9I3V8 | Bacillus cereus |
| A0A1Q9PPU2 | Alkalihalobacillus pseudofirmus |
| A0A1Q9Q3V9 | Bacillus sp. MRMR6 |
| A0A1R0WYB3 | Paenibacillus odorifer |
| A0A1R0XT94 | Paenibacillus odorifer |
| A0A1R0Y423 | Paenibacillus odorifer |
| A0A1R0YXC6 | Paenibacillus odorifer |
| A0A1R0ZAD2 | Paenibacillus odorifer |
| A0A1R1A975 | Paenibacillus lautus |
| A0A1R1APE4 | Paenibacillus sp. FSL A5-0031 |
| A0A1R1D0W9 | Paenibacillus sp. FSL H8-0548 |
| A0A1R1EPP2 | Paenibacillus rhizosphaerae |
| A0A1R1ETF9 | Paenibacillus sp. FSL R5-0490 |
| A0A1R1GPK8 | Paenibacillus sp. FSL R7-0337 |
| A0A1S1FML9 | Bacillus sp. HMSC76G11 |
| A0A1S1YGZ1 | Cytobacillus oceanisediminis |
| A0A1S2F9E4 | Paenibacillus sp. LC231 |
| A0A1S2LJR3 | Anaerobacillus arseniciselenatis |
| A0A1S2LMJ2 | Anaerobacillus alkalilacustris |
| A0A1S2M2M6 | Anaerobacillus alkalidiazotrophicus |
| A0A1S2R4N1 | Bacillus sp. MUM 13 |
| A0A1S2REB7 | Bacillus sp. MUM 116 |
| A0A1S6IZB5 | Desulfotomaculum ferrireducens |
| A0A1S8G6G6 | Bacillus mycoides |
| A0A1S9CD67 | Epulopiscium sp. AS2M-Bin001 |
| A0A1S9CJQ8 | Epulopiscium sp. Nele67-Bin005 |
| A0A1S9I655 | Clostridium tepidum |
| A0A1S9TRK3 | Bacillus cereus |
| A0A1S9V6Y2 | Bacillus cereus |
| A0A1S9XRV9 | Bacillus mycoides |
| A0A1T2P9U8 | Bacillus cereus |
| A0A1T2PLB9 | Bacillus cereus |
| A0A1T2T2H4 | Bacillus cereus |
| A0A1T2X1Y8 | Paenibacillus selenitireducens |
| A0A1T3V387 | Bacillus anthracis |
| A0A1T4KQ78 | Garciella nitratireducens DSM 15102 |
| A0A1T4M1C6 | Carboxydocella sporoproducens DSM 16521 |
| A0A1T4N5I1 | Selenihalanaerobacter shriftii |
| A0A1T4W911 | Clostridium sp. USBA 49 |
| A0A1T4XEZ7 | Caloramator quimbayensis |
| A0A1T4Y6S3 | Sporosarcina newyorkensis |
| A0A1T4ZVG8 | Lysinibacillus sp. AC-3 |
| A0A1T5KRS1 | Maledivibacter halophilus |
| A0A1T5LV26 | Maledivibacter halophilus |
| A0A1U6JH99 | Bacillus sp. V-88 |
| A0A1U7M820 | Tissierella creatinophila DSM 6911 |
| A0A1U7MMM7 | Sporomusa sphaeroides DSM 2875 |
| A0A1U7PJX6 | Edaphobacillus lindanitolerans |
| A0A1U9K7A3 | Novibacillus thermophilus |
| A0A1V1HY46 | Romboutsia ilealis |
| A0A1V2A6A5 | Domibacillus epiphyticus |
| A0A1V2HDL6 | Bacillus cereus |
| A0A1V2M959 | Epulopiscium sp. SCG-C07WGA-EpuloA2 |
| A0A1V2SKR6 | Bacillus sp. VT-16-64 |
| A0A1V2YCU8 | Epulopiscium sp. Nuni2H_MBin003 |
| A0A1V2YJR5 | Epulopiscium sp. Nuni2H_MBin003 |
| A0A1V2YJY4 | Epulopiscium sp. Nuni2H_MBin001 |
| A0A1V3G4H6 | Fictibacillus arsenicus |
| A0A1V4I8L2 | [Clostridium] thermoalcaliphilum |
| A0A1V4SRX6 | Ruminiclostridium hungatei |
| A0A1V4SU42 | Clostridium thermobutyricum DSM 4928 |
| A0A1V4VLK6 | Pelotomaculum sp. PtaB.Bin117 |
| A0A1V4VUH2 | Pelotomaculum sp. PtaB.Bin104 |
| A0A1V5BQQ0 | Pelotomaculum sp. PtaU1.Bin065 |
| A0A1V5BTT9 | Pelotomaculum sp. PtaU1.Bin065 |
| A0A1V5KFX2 | Firmicutes bacterium ADurb.Bin467 |
| A0A1V5L119 | Firmicutes bacterium ADurb.Bin456 |
| A0A1V5MLC7 | Firmicutes bacterium ADurb.Bin419 |
| A0A1V5S7A8 | Firmicutes bacterium ADurb.Bin300 |
| A0A1V5TZH5 | Firmicutes bacterium ADurb.Bin248 |
| A0A1V5XFX1 | Firmicutes bacterium ADurb.Bin193 |
| A0A1V5YBX9 | Firmicutes bacterium ADurb.Bin182 |
| A0A1V9IQ78 | Clostridium sporogenes |
| A0A1V9W066 | Bacillus sp. CDB3 |
| A0A1W1UHE8 | Desulfonispora thiosulfatigenes DSM 11270 |
| A0A1W1UHI0 | Desulfonispora thiosulfatigenes DSM 11270 |
| A0A1W1W2L7 | Thermanaeromonas toyohensis ToBE |
| A0A1W1W793 | Sulfobacillus thermosulfidooxidans (strain DSM 9293 / VKM B-1269 / AT-1) |
| A0A1W1Z1E7 | Papillibacter cinnamivorans DSM 12816 |
| A0A1W2BSZ8 | Sporomusa malonica |
| A0A1W2GW40 | Bacillus sp. JKS001846 |
| A0A1W6A2X6 | Bacillus mycoides |
| A0A1X3MIN9 | Bacillus toyonensis |
| A0A1X7G627 | Paenibacillus uliginis N3/975 |
| A0A1X9MAY4 | Alkalihalobacillus krulwichiae |
| A0A1Y0CSK5 | Bacillus horikoshii |
| A0A1Y0TWD8 | Bacillus thuringiensis |
| A0A1Y2T509 | Symbiobacterium thermophilum |
| A0A1Y3ML41 | Bacillus pseudomycoides |
| A0A1Y3RJ62 | Flavonifractor sp. An91 |
| A0A1Y3RN37 | Flavonifractor sp. An9 |
| A0A1Y3RU35 | Gemmiger sp. An87 |
| A0A1Y3SNW4 | Flavonifractor sp. An82 |
| A0A1Y3SVF6 | Pseudoflavonifractor sp. An85 |
| A0A1Y3TAG8 | Faecalibacterium sp. An77 |
| A0A1Y3WVM8 | Faecalibacterium sp. An58 |
| A0A1Y3XHS1 | Flavonifractor sp. An52 |
| A0A1Y3XKU1 | Gemmiger sp. An50 |
| A0A1Y3YMJ5 | Pseudoflavonifractor sp. An44 |
| A0A1Y3ZTH2 | Flavonifractor sp. An4 |
| A0A1Y4C6G8 | Flavonifractor sp. An306 |
| A0A1Y4EVI7 | Anaeromassilibacillus sp. An250 |
| A0A1Y4FPP0 | Flavonifractor plautii |
| A0A1Y4HEB1 | Anaerofilum sp. An201 |
| A0A1Y4HZM4 | Anaeromassilibacillus sp. An200 |
| A0A1Y4ISQ3 | Gemmiger sp. An194 |
| A0A1Y4J6T6 | Faecalibacterium sp. An192 |
| A0A1Y4KBK2 | Pseudoflavonifractor sp. An187 |
| A0A1Y4LQJ8 | Pseudoflavonifractor sp. An184 |
| A0A1Y4M3H2 | Pseudoflavonifractor sp. An176 |
| A0A1Y4RWX2 | Flavonifractor sp. An135 |
| A0A1Y4T4A1 | Faecalibacterium sp. An121 |
| A0A1Y4TCI6 | Faecalibacterium sp. An122 |
| A0A1Y4TNC5 | Gemmiger sp. An120 |
| A0A1Y4UF12 | Flavonifractor sp. An112 |
| A0A1Y4WLT8 | Flavonifractor sp. An10 |
| A0A1Y4WM03 | Flavonifractor sp. An100 |
| A0A1Y4X3Z3 | Brevibacillus brevis |
| A0A1Y5K2Z6 | Paenibacillus sp. MY03 |
| A0A1Y5Z8A0 | Bacillus mobilis |
| A0A1Y5ZW25 | Bacillus cereus |
| A0A1Y6E7Z6 | Bacillus sp. OV166 |
| A0A1Z5HR28 | Calderihabitans maritimus |
| A0A1Z5HRM8 | Calderihabitans maritimus |
| A0A212J6A5 | uncultured Eubacteriales bacterium |
| A0A212LQJ6 | uncultured Sporomusa sp |
| A0A220MBR1 | Brevibacillus formosus |
| A0A220U462 | Virgibacillus phasianinus |
| A0A221BRN2 | Bacillus cereus |
| A0A221MAN7 | Virgibacillus necropolis |
| A0A223EHG3 | Peribacillus simplex NBRC 15720 = DSM 1321 |
| A0A223KWL1 | Bacillus cohnii |
| A0A226BW94 | Natranaerobius trueperi |
| A0A226QXI9 | Bacillus sp. M13(2017) |
| A0A229MA73 | Bacillus sp. KbaL1 |
| A0A231I5E7 | Bacillus thuringiensis |
| A0A231REP0 | Cohnella sp. CIP 111063 |
| A0A231W5H4 | Bacillus sp. OG2 |
| A0A235FBB7 | Fictibacillus aquaticus |
| A0A239HN74 | Bacillus sp. OK838 |
| A0A239JX23 | Anaerovirgula multivorans |
| A0A242WES8 | Bacillus thuringiensis serovar mexicanensis |
| A0A242WFM2 | Bacillus thuringiensis serovar cameroun |
| A0A242XPL1 | Bacillus thuringiensis serovar guiyangiensis |
| A0A242Y852 | Bacillus thuringiensis serovar novosibirsk |
| A0A242Z661 | Bacillus wiedmannii |
| A0A242ZR82 | Bacillus thuringiensis serovar kim |
| A0A243AJR2 | Bacillus thuringiensis serovar navarrensis |
| A0A243AYX9 | Bacillus thuringiensis serovar poloniensis |
| A0A243BHM2 | Bacillus thuringiensis serovar pingluonsis |
| A0A243C540 | Bacillus thuringiensis serovar yosoo |
| A0A243D230 | Bacillus thuringiensis serovar vazensis |
| A0A243D8H4 | Bacillus thuringiensis serovar subtoxicus |
| A0A243DZM3 | Bacillus thuringiensis subsp. darmstadiensis |
| A0A243E7Y0 | Bacillus thuringiensis serovar toumanoffi |
| A0A243F0A6 | Bacillus thuringiensis subsp. kumamotoensis |
| A0A243GLN5 | Bacillus thuringiensis subsp. finitimus |
| A0A243IW53 | Bacillus thuringiensis subsp. konkukian |
| A0A243JCV4 | Bacillus thuringiensis serovar pirenaica |
| A0A243KSL6 | Bacillus thuringiensis subsp. higo |
| A0A243KST8 | Bacillus thuringiensis serovar argentinensis |
| A0A243L1P4 | Bacillus thuringiensis serovar iberica |
| A0A243LH44 | Bacillus thuringiensis subsp. jegathesan |
| A0A243MNK8 | Bacillus thuringiensis serovar zhaodongensis |
| A0A243NFL2 | Bacillus thuringiensis subsp. medellin |
| A0A246PPY5 | Bacillus sp. K2I17 |
| A0A249X6P6 | Bacillus cereus |
| A0A259TBR9 | Paenibacillus sp. XY044 |
| A0A259UC19 | Sporomusa acidovorans DSM 3132 |
| A0A259UJE8 | Sporomusa silvacetica DSM 10669 |
| A0A261QKA1 | Bacillaceae bacterium SAS-127 |
| A0A263BSZ4 | Lottiidibacillus patelloidae |
| A0A263BVX5 | Lottiidibacillus patelloidae |
| A0A264DDD2 | Paenibacillus sp. VTT E-133280 |
| A0A264EBE1 | Paenibacillus odorifer |
| A0A265NAG4 | Virgibacillus indicus |
| A0A265QAQ8 | Tissierella sp. P1 |
| A0A267MHD8 | Anaeromicrobium sediminis |
| A0A267MHP5 | Anaeromicrobium sediminis |
| A0A267MJL2 | Anaeromicrobium sediminis |
| A0A267TPE6 | Caldibacillus hisashii |
| A0A268E5V2 | Bacillus sp. 7586-K |
| A0A268IBL0 | Bacillus sp. 7504-2 |
| A0A268IVB9 | Bacillus sp. 7894-2 |
| A0A268KAD0 | Bacillus sp. 7884-1 |
| A0A270AKB4 | Peribacillus simplex |
| A0A271M5K9 | Bacillaceae bacterium SAOS 7 |
| A0A285CIQ7 | Bacillus oleivorans |
| A0A285GKD8 | Orenia metallireducens |
| A0A285STC0 | Ureibacillus xyleni |
| A0A285UCY4 | Ureibacillus acetophenoni |
| A0A291BG03 | Brevibacillus brevis X23 |
| A0A291JGS9 | Staphylococcus nepalensis |
| A0A291TDQ9 | Faecalibacterium prausnitzii |
| A0A2A2IH90 | Virgibacillus profundi |
| A0A2A2PDV7 | Bacillus toyonensis |
| A0A2A4BH91 | Peribacillus simplex |
| A0A2A5LA92 | Paenibacillus lautus |
| A0A2A6Z975 | Faecalibacterium prausnitzii |
| A0A2A6ZHL0 | Faecalibacterium prausnitzii |
| A0A2A6ZWI1 | Faecalibacterium prausnitzii |
| A0A2A7AE65 | Faecalibacterium prausnitzii |
| A0A2A7AQI1 | Faecalibacterium prausnitzii |
| A0A2A7AWL6 | Faecalibacterium prausnitzii |
| A0A2A7B591 | Faecalibacterium prausnitzii |
| A0A2A7BEP3 | Faecalibacterium prausnitzii |
| A0A2A7BV03 | Bacillus wiedmannii |
| A0A2A7DEM5 | Bacillus anthracis |
| A0A2A7EDJ2 | Bacillus sp. AFS094611 |
| A0A2A7HAI2 | Bacillus sp. AFS098217 |
| A0A2A7HTZ5 | Bacillus cereus |
| A0A2A7IH20 | Bacillus sp. AFS096315 |
| A0A2A7II50 | Bacillus cereus |
| A0A2A7WZZ4 | Bacillus sp. AFS002410 |
| A0A2A7X994 | Bacillus wiedmannii |
| A0A2A7ZLW7 | Bacillus cereus |
| A0A2A8AWH7 | Bacillus wiedmannii |
| A0A2A8E333 | Bacillus cereus |
| A0A2A8FQB8 | Bacillus sp. AFS026049 |
| A0A2A8G170 | Bacillus wiedmannii |
| A0A2A8HLR4 | Bacillus toyonensis |
| A0A2A8ILY2 | Bacillus sp. AFS006103 |
| A0A2A8ISM0 | Bacillus cereus |
| A0A2A8MS79 | Bacillus sp. AFS001701 |
| A0A2A8NJR9 | Bacillus thuringiensis |
| A0A2A8PSJ6 | Bacillus cereus |
| A0A2A8S2B7 | Bacillus cereus |
| A0A2A8SBK7 | Bacillus sp. AFS018417 |
| A0A2A8TRY3 | Bacillus sp. AFS017274 |
| A0A2A8UBR5 | Bacillus cereus |
| A0A2A8UTJ5 | Bacillus sp. AFS015896 |
| A0A2A8UZM5 | Bacillus sp. AFS015802 |
| A0A2A9A748 | Bacillus cereus |
| A0A2A9BY70 | Bacillus sp. es.034 |
| A0A2A9RE12 | Bacillus sp. AFS088145 |
| A0A2A9XA34 | Bacillus cereus |
| A0A2B0C3G2 | Bacillus cereus |
| A0A2B0L4X9 | Bacillus cereus |
| A0A2B0M9N4 | Bacillus cereus |
| A0A2B0WYF9 | Bacillus cereus |
| A0A2B0Y802 | Bacillus anthracis |
| A0A2B1DDL6 | Bacillus cereus |
| A0A2B1DVW1 | Bacillus cereus |
| A0A2B1KQ32 | Bacillus cereus |
| A0A2B1RFM2 | Bacillus cereus |
| A0A2B1VEH4 | Bacillus cereus |
| A0A2B2C870 | Bacillus sp. AFS073361 |
| A0A2B2GL24 | Bacillus cereus |
| A0A2B2LTU8 | Bacillus cereus |
| A0A2B3EJV2 | Bacillus thuringiensis |
| A0A2B3LAL9 | Bacillus thuringiensis |
| A0A2B3UBJ6 | Bacillus cereus |
| A0A2B4FGT8 | Bacillus sp. AFS059628 |
| A0A2B4LEL1 | Bacillus cereus |
| A0A2B4P2J7 | Bacillus sp. AFS075960 |
| A0A2B4XUJ2 | Bacillus mycoides |
| A0A2B5IUJ3 | Bacillus wiedmannii |
| A0A2B5J402 | Bacillus wiedmannii |
| A0A2B5KD50 | Bacillus wiedmannii |
| A0A2B5XRJ4 | Bacillus wiedmannii |
| A0A2B6CB44 | Bacillus anthracis |
| A0A2B6NIU4 | Bacillus toyonensis |
| A0A2B6QHE4 | Bacillus toyonensis |
| A0A2B6R361 | Bacillus pseudomycoides |
| A0A2B8G019 | Bacillus cereus |
| A0A2B8ITP8 | Bacillus anthracis |
| A0A2B8LWR8 | Bacillus cereus |
| A0A2B8U1N7 | Bacillus sp. AFS055030 |
| A0A2B9AHC7 | Bacillus sp. AFS053548 |
| A0A2B9BAK0 | Bacillus cereus |
| A0A2B9DKF8 | Bacillus cereus |
| A0A2B9ELR3 | Bacillus cereus |
| A0A2B9MXX8 | Bacillus cereus |
| A0A2B9PD52 | Bacillus thuringiensis |
| A0A2B9PKS3 | Bacillus cereus |
| A0A2B9UAJ3 | Bacillus cereus |
| A0A2B9XDW2 | Bacillus thuringiensis |
| A0A2C0CGH1 | Bacillus cereus |
| A0A2C0ZJI7 | Bacillus sp. AFS041924 |
| A0A2C1AHU8 | Bacillus cereus |
| A0A2C1D606 | Bacillus cereus |
| A0A2C1L0U2 | Bacillus sp. AFS040349 |
| A0A2C1YG79 | Bacillus cereus |
| A0A2C1YWT2 | Bacillus sp. AFS037270 |
| A0A2C2ACV3 | Bacillus cereus |
| A0A2C2C3G3 | Bacillus cereus |
| A0A2C2UWY0 | Bacillus cereus |
| A0A2C2VD57 | Bacillus sp. AFS031507 |
| A0A2C3G960 | Bacillus anthracis |
| A0A2C4Q0S2 | Bacillus wiedmannii |
| A0A2C4QUS1 | Bacillus toyonensis |
| A0A2C9YWB3 | Bacillus thuringiensis subsp. kyushuensis |
| A0A2D1SZN4 | Solibacillus sp. R5-41 |
| A0A2G2M9A1 | Alkaliphilus sp |
| A0A2G3PYK1 | Lachnospiraceae bacterium |
| A0A2G5W133 | Sporosarcina sp. P10 |
| A0A2G5WL10 | Sporosarcina sp. P13 |
| A0A2G5WS38 | Sporosarcina sp. P16a |
| A0A2G5X4D5 | Sporosarcina sp. P16b |
| A0A2G5XCZ7 | Sporosarcina sp. P17b |
| A0A2G5XJ69 | Sporosarcina sp. P19 |
| A0A2G5ZBA1 | Sporosarcina sp. P29 |
| A0A2G6AQU1 | Sporosarcina sp. P34 |
| A0A2G6B9H5 | Sporosarcina sp. P3 |
| A0A2G6QCC1 | Bacillus fungorum |
| A0A2G7HGT3 | Clostridium combesii |
| A0A2H3M8S4 | Bacillus pseudomycoides |
| A0A2H3QV93 | Bacillus sp. AFS012607 |
| A0A2I0R7A2 | Bacillus cereus Rock4-18 |
| A0A2I0V659 | Lysinibacillus fusiformis |
| A0A2I4NHD5 | Clostridium botulinum |
| A0A2J4JRY0 | Faecalibacterium prausnitzii |
| A0A2J9DL65 | Bacillus thuringiensis |
| A0A2K2FMQ3 | Pseudoclostridium thermosuccinogenes |
| A0A2K8TKH8 | Bacillus cereus |
| A0A2K8ZED8 | Bacillus sp. HBCD-sjtu |
| A0A2K9E3N6 | Acetivibrio saccincola |
| A0A2K9MUU7 | Clostridium sporogenes |
| A0A2K9P4M5 | Monoglobus pectinilyticus |
| A0A2L1GLY3 | Desulfobulbus oralis |
| A0A2L2XDB8 | Desulfocucumis palustris |
| A0A2M9ML56 | Paenibacillus sp. GM2FR |
| A0A2M9P0K1 | Bacillus sp. mrc49 |
| A0A2M9P1F7 | Bacillus sp. mrc49 |
| A0A2M9P1G9 | Bacillus sp. mrc49 |
| A0A2M9Q2R8 | Lysinibacillus xylanilyticus |
| A0A2M9X824 | Bacillus cereus |
| A0A2N0F8U6 | Viridibacillus sp. OK051 |
| A0A2N0Y8R8 | Bacillus sp. BA3 |
| A0A2N1JVA8 | Bacillus sp. SN10 |
| A0A2N2AZS9 | Firmicutes bacterium HGW-Firmicutes-7 |
| A0A2N2BZ01 | Firmicutes bacterium HGW-Firmicutes-21 |
| A0A2N2CV14 | Firmicutes bacterium HGW-Firmicutes-16 |
| A0A2N2D3E3 | Firmicutes bacterium HGW-Firmicutes-15 |
| A0A2N2D566 | Firmicutes bacterium HGW-Firmicutes-15 |
| A0A2N2DCM7 | Firmicutes bacterium HGW-Firmicutes-14 |
| A0A2N2DLY1 | Firmicutes bacterium HGW-Firmicutes-13 |
| A0A2N2E3B8 | Firmicutes bacterium HGW-Firmicutes-12 |
| A0A2N2EG20 | Firmicutes bacterium HGW-Firmicutes-1 |
| A0A2N3LJI1 | Bacillus camelliae |
| A0A2N3NQM5 | Bacillus sp. BI3 |
| A0A2N5FJF8 | Bacillus sp. UMB0893 |
| A0A2N5FY00 | Bacillus sp. UMB0728 |
| A0A2N5GE75 | Bacillus sp. V3-13 |
| A0A2N5GH83 | Bacillus canaveralius |
| A0A2N5GZV2 | Bacillus sp. T33-2 |
| A0A2N5HEA0 | Neobacillus cucumis |
| A0A2N5I912 | Bacillus sp. M6-12 |
| A0A2N5M5S1 | Peribacillus deserti |
| A0A2N5MNK4 | Bacillus sp. V5-8f |
| A0A2N6RLW4 | Bacillus sp. UMB0899 |
| A0A2P1THR6 | Clostridium botulinum |
| A0A2P1WPY9 | Oceanobacillus iheyensis |
| A0A2P2BMP6 | Romboutsia hominis |
| A0A2P7UJU8 | Brevibacillus fortis |
| A0A2R4N1Y7 | Carboxydocella thermautotrophica |
| A0A2R5EV86 | Paenibacillus agaridevorans |
| A0A2S0JGS4 | Lysinibacillus sp. B2A1 |
| A0A2S0K5C2 | Lysinibacillus sphaericus |
| A0A2S1A6H4 | Bacillus cytotoxicus |
| A0A2S3QGA7 | Sulfobacillus sp. hq2 |
| A0A2S5D5A1 | Lysinibacillus sphaericus |
| A0A2S5G9Y4 | Jeotgalibacillus proteolyticus |
| A0A2S5I3X3 | Brevibacillus laterosporus |
| A0A2S6G021 | Clostridium algidicarnis DSM 15099 |
| A0A2S8RDK9 | Acetivibrio saccincola |
| A0A2S8ULQ8 | Bacillus sp. MYb209 |
| A0A2S8VTN1 | Bacillus sp. MYb78 |
| A0A2S9H9I8 | Bacillus cereus |
| A0A2S9HTJ4 | Bacillus sp. MYb56 |
| A0A2S9Y3T3 | Bacillus sp. M21 |
| A0A2T0ATZ0 | Clostridium thermopalmarium DSM 5974 |
| A0A2T0AVH7 | Moorella humiferrea |
| A0A2T0B2S3 | Clostridium liquoris |
| A0A2T0BND5 | Clostridium luticellarii |
| A0A2T0DZ65 | Bacillus toyonensis |
| A0A2T0EY66 | Bacillus thuringiensis |
| A0A2T2WSB6 | Sulfobacillus thermosulfidooxidans |
| A0A2T2X9B7 | Sulfobacillus benefaciens |
| A0A2T2XCW4 | Sulfobacillus benefaciens |
| A0A2T4SAZ4 | Staphylococcus nepalensis |
| A0A2T5UND8 | Bacillus sp. OV752 |
| A0A2T6EGR9 | Bacillus sporothermodurans |
| A0A2T6JSF6 | Paenisporosarcina sp. OV554 |
| A0A2T7YM27 | Bacillus thuringiensis |
| A0A2U1CD65 | Intestinimonas butyriciproducens |
| A0A2U1K0A2 | Pueribacillus theae |
| A0A2U3L9L7 | Candidatus Desulfosporosinus infrequens |
| A0A2U8DXJ4 | Clostridium drakei |
| A0A2U8ECB0 | Caldibacillus thermoamylovorans |
| A0A2V2CFN9 | Clostridia bacterium |
| A0A2V2D263 | Clostridiales bacterium |
| A0A2V2DZA7 | Clostridiales bacterium |
| A0A2V2E2X6 | Clostridiales bacterium |
| A0A2V2EVA5 | Clostridiales bacterium |
| A0A2V2FAV6 | Oscillospiraceae bacterium |
| A0A2V2FZ12 | Clostridiales Family XIII bacterium |
| A0A2V2G642 | Clostridiales bacterium |
| A0A2V2GP49 | Oscillospiraceae bacterium |
| A0A2V3A2Z5 | Cytobacillus oceanisediminis |
| A0A2V3W5G7 | Pseudogracilibacillus auburnensis |
| A0A2W0H6T2 | Bacillus lacisalsi |
| A0A2W1NRL8 | Paenibacillus xerothermodurans |
| A0A2W4K387 | Firmicutes bacterium |
| A0A2W4KCH9 | Firmicutes bacterium |
| A0A2W4KN14 | Firmicutes bacterium |
| A0A2W4MLX1 | Caldicoprobacter oshimai |
| A0A2W4NA88 | Firmicutes bacterium |
| A0A2W4QJI6 | Firmicutes bacterium |
| A0A2W6N1B4 | Clostridium perfringens |
| A0A2W7N3B1 | Psychrobacillus insolitus |
| A0A2X0YUK1 | Lysinibacillus capsici |
| A0A2X2WD83 | Clostridium cochlearium |
| A0A2X2WFW3 | Clostridium perfringens |
| A0A2X2Y040 | Clostridium perfringens |
| A0A2X4ZEY1 | Lederbergia lentus |
| A0A2Z4MEI0 | Brevibacillus brevis |
| A0A2Z4W7Y4 | Clostridiaceae bacterium 14S0207 |
| A0A316LC94 | Clostridiales bacterium |
| A0A316N0M8 | Oscillospiraceae bacterium |
| A0A316PHE9 | Oscillospiraceae bacterium |
| A0A316Q1U9 | Clostridiales bacterium |
| A0A316Q8Q5 | Clostridiales bacterium |
| A0A316QIS8 | Clostridiales bacterium |
| A0A316QL15 | Clostridiales bacterium |
| A0A316RHL2 | Oscillospiraceae bacterium |
| A0A316RWG6 | Oscillospiraceae bacterium |
| A0A316T704 | Massilioclostridium sp |
| A0A317KT67 | Gracilibacillus dipsosauri |
| A0A317TQ04 | Clostridium perfringens |
| A0A318THP6 | Ureibacillus chungkukjangi |
| A0A323TDH3 | Salipaludibacillus keqinensis |
| A0A327S649 | Bacillus sp. YR335 |
| A0A328KTD5 | Bacillus sp. SRB_8 |
| A0A328LFT4 | Bacillus sp. SRB_331 |
| A0A328TZK2 | Paenibacillus montanisoli |
| A0A328UM82 | Hydrogeniiclostidium mannosilyticum |
| A0A328WBS4 | Paenibacillus lautus |
| A0A329L399 | Paenibacillus sp. YN15 |
| A0A329MK52 | Paenibacillus contaminans |
| A0A329TG41 | Faecalibacterium prausnitzii |
| A0A329TU74 | Faecalibacterium prausnitzii |
| A0A329TYW0 | Faecalibacterium prausnitzii |
| A0A329U8K4 | Faecalibacterium prausnitzii |
| A0A329U9F6 | Faecalibacterium prausnitzii |
| A0A329UL90 | Faecalibacterium prausnitzii |
| A0A329UXQ3 | Faecalibacterium prausnitzii |
| A0A336QS83 | Clostridium perfringens |
| A0A343J9Y3 | Clostridium isatidis |
| A0A345BZ74 | Salicibibacter kimchii |
| A0A345P196 | Sporosarcina sp. PTS2304 |
| A0A345PJ59 | Oceanobacillus zhaokaii |
| A0A345X2R3 | Bacillus sp. COPE52 |
| A0A347V4E7 | Bacillus thuringiensis LM1212 |
| A0A348AFU3 | Methylomusa anaerophila |
| A0A348P5U0 | Oscillospiraceae bacterium |
| A0A348Z7V0 | Clostridium sp. |
| A0A349DBV8 | Oscillospiraceae bacterium |
| A0A349HQI6 | Clostridiales bacterium |
| A0A349PV89 | Oscillibacter sp |
| A0A349Q832 | Clostridiales bacterium |
| A0A349U4N8 | Desulfotomaculum sp |
| A0A349YM96 | Lachnospiraceae bacterium |
| A0A350BFC8 | Firmicutes bacterium |
| A0A350NP44 | Firmicutes bacterium |
| A0A350WZJ4 | Firmicutes bacterium |
| A0A351ED52 | Oscillospiraceae bacterium |
| A0A351F9J4 | Oscillospiraceae bacterium |
| A0A351J1F0 | Clostridiales bacterium |
| A0A351J6K4 | Firmicutes bacterium |
| A0A351KH25 | Clostridiaceae bacterium |
| A0A351QW08 | Clostridium sp. |
| A0A352CXV3 | Ruminococcus sp |
| A0A352IKU0 | Clostridiales bacterium |
| A0A352NFS8 | Pelotomaculum sp |
| A0A352RLV5 | Oscillibacter sp |
| A0A352SVM3 | Clostridiales bacterium |
| A0A352UNA3 | Clostridiales bacterium |
| A0A353EWN2 | Clostridiales bacterium |
| A0A353HAX4 | Clostridiales bacterium |
| A0A353K333 | Clostridiaceae bacterium |
| A0A353M1U8 | Firmicutes bacterium |
| A0A353MQ25 | Firmicutes bacterium |
| A0A353QJM9 | Firmicutes bacterium |
| A0A353T297 | Clostridiales bacterium |
| A0A354FE88 | Peptococcaceae bacterium |
| A0A354FFD0 | Peptococcaceae bacterium |
| A0A354HTE7 | Firmicutes bacterium |
| A0A354KV82 | Terrisporobacter glycolicus |
| A0A354MXH6 | Clostridiales bacterium |
| A0A354YWL8 | Syntrophomonas wolfei |
| A0A354YZH7 | Syntrophomonas wolfei |
| A0A355BIC4 | Firmicutes bacterium |
| A0A355D522 | Clostridium sp. |
| A0A355FSA5 | Firmicutes bacterium |
| A0A355GQV5 | Firmicutes bacterium |
| A0A355KU56 | Oscillospiraceae bacterium |
| A0A355S2K7 | Clostridiaceae bacterium |
| A0A355SCQ2 | Clostridiaceae bacterium |
| A0A356B4R0 | Clostridiales bacterium |
| A0A356BUG1 | Firmicutes bacterium |
| A0A356CZ28 | Ruminococcus sp |
| A0A356GG05 | Clostridiales bacterium |
| A0A356P5S4 | Desulfosporosinus sp |
| A0A356PU05 | Oscillospiraceae bacterium |
| A0A356U822 | Syntrophomonas sp |
| A0A356UC60 | Syntrophomonas sp |
| A0A356UH50 | Desulfotomaculum sp |
| A0A356XG36 | Clostridiales bacterium |
| A0A356Z544 | Syntrophomonas sp |
| A0A356ZD95 | Syntrophomonas sp |
| A0A357AMG0 | Ruminiclostridium sp |
| A0A357AXI3 | Clostridiales bacterium |
| A0A357CYZ1 | Clostridiales bacterium |
| A0A357D4I5 | Firmicutes bacterium |
| A0A357M3W6 | Paenibacillus sp. |
| A0A357R5N9 | Firmicutes bacterium |
| A0A357T4X7 | Firmicutes bacterium |
| A0A357TE93 | Peptococcaceae bacterium |
| A0A357WTF0 | Oscillospiraceae bacterium |
| A0A357WU76 | Oscillospiraceae bacterium |
| A0A358LJQ9 | Oscillospiraceae bacterium |
| A0A358M392 | Clostridiales bacterium |
| A0A358PZD7 | Desulfosporosinus sp |
| A0A358Q0A3 | Desulfosporosinus sp |
| A0A358QZN1 | Desulfotomaculum sp |
| A0A358RK06 | Clostridiales bacterium |
| A0A358TZ47 | Desulfosporosinus sp |
| A0A358U2Q8 | Desulfosporosinus sp |
| A0A359B8Z7 | Desulfotomaculum sp |
| A0A359CPP1 | Clostridiaceae bacterium |
| A0A366F230 | Bacillus aquimaris |
| A0A366G4Y6 | Bacillus sp. DB-2 |
| A0A366ICG1 | Alkalibaculum bacchi |
| A0A366JYS9 | Cytobacillus firmus |
| A0A366XV32 | Bacillus taeanensis |
| A0A368WKU2 | Bacillus sp. NFR08 |
| A0A369BER6 | Fontibacillus phaseoli |
| A0A369CT49 | Bacillus sp. AG102 |
| A0A370GHS8 | Falsibacillus pallidus |
| A0A371IVE6 | Romboutsia maritimum |
| A0A371J1F8 | Romboutsia weinsteinii |
| A0A371P7N4 | Paenibacillus paeoniae |
| A0A371SGS9 | Bacillus sp. HNG |
| A0A372LAQ3 | Bacillus glennii |
| A0A372LNH2 | Bacillus saganii |
| A0A372V9C9 | Subdoligranulum sp. AM16-9 |
| A0A372VBR2 | Subdoligranulum sp. AM16-9 |
| A0A372X1Q7 | Subdoligranulum sp. AM23-21AC |
| A0A372X241 | Subdoligranulum sp. AM23-21AC |
| A0A373LBB0 | Ruminococcus sp. AF37-20 |
| A0A373MU10 | Ruminococcus sp. AF34-12 |
| A0A373NBN2 | Faecalibacterium sp. OF04-11AC |
| A0A373Q021 | Ruminococcus sp. AM54-1NS |
| A0A373SIT7 | Ruminococcus sp. AF25-19 |
| A0A373UCK2 | Ruminococcus sp. AF21-11 |
| A0A373V6B2 | Ruminococcus sp. AF19-15 |
| A0A373VX01 | Ruminococcus sp. AF18-29 |
| A0A373WES9 | Ruminococcus sp. AF17-6 |
| A0A373XXP2 | Ruminococcus sp. AF16-50 |
| A0A374B7G4 | Ruminococcus sp. AM47-2BH |
| A0A374BTQ5 | Ruminococcus sp. AM43-6 |
| A0A374EVK4 | Ruminococcus sp. AM31-15AC |
| A0A374G9L4 | Ruminococcus sp. AM28-13 |
| A0A374HKU9 | Ruminococcus sp. TF12-2 |
| A0A380BE16 | Sporosarcina pasteurii |
| A0A380Y8S7 | Cytobacillus firmus |
| A0A381J0P5 | Clostridium perfringens |
| A0A381J564 | Clostridium putrefaciens |
| A0A385NVZ8 | Bacillus sp. Y1 |
| A0A385T4M6 | Brevibacillus laterosporus |
| A0A385TWI6 | Paenibacillus lautus |
| A0A385YSV1 | Paenisporosarcina sp. K2R23-3 |
| A0A386PGB6 | Clostridium septicum |
| A0A386XPA5 | Ethanoligenens harbinense |
| A0A386YMD8 | Clostridium novyi |
| A0A386ZX63 | Bacillus thuringiensis |
| A0A396LHN8 | Faecalibacterium sp. OF03-6AC |
| A0A396SLF4 | Lysinibacillus yapensis |
| A0A398BBJ0 | Mesobacillus zeae |
| A0A398BI76 | Peribacillus asahii |
| A0A3A0SB19 | Staphylococcus nepalensis |
| A0A3A1QVE5 | Bacillus salacetis |
| A0A3A1UV74 | Paenibacillus nanensis |
| A0A3A4U4F7 | Firmicutes bacterium |
| A0A3A5IIS4 | Bacillus sp. PK3_68 |
| A0A3A6CP95 | Faecalibacterium sp. AF27-11BH |
| A0A3A6EDQ9 | Faecalibacterium sp. AM43-5AT |
| A0A3A6EEW4 | Subdoligranulum sp. AF14-43 |
| A0A3A6EVM9 | Subdoligranulum sp. AF14-43 |
| A0A3A6JQ26 | Faecalibacterium sp. AF10-46 |
| A0A3A6JX76 | Subdoligranulum sp. OF01-18 |
| A0A3A6JXB8 | Subdoligranulum sp. OF01-18 |
| A0A3A6MN37 | Candidatus Desulforudis sp |
| A0A3A6N4I8 | Ammonifex sp |
| A0A3A6N9D5 | Dethiobacter sp |
| A0A3A6PL26 | Paenibacillus pinisoli |
| A0A3A8YVX8 | bacterium 1xD42-67 |
| A0A3A8YX15 | bacterium 1xD42-67 |
| A0A3A9FU49 | bacterium 1XD42-8 |
| A0A3A9J3Q2 | Anaerotruncus sp. 1XD22-93 |
| A0A3A9JB24 | Anaerotruncus sp. 1XD22-93 |
| A0A3A9JV21 | Thermoanaerobacteraceae bacterium SP2 |
| A0A3B0CBH9 | Paenibacillus ginsengarvi |
| A0A3B8HTT2 | Syntrophomonas sp |
| A0A3B8HZH0 | Syntrophomonas sp |
| A0A3B8I0G7 | Syntrophomonas sp |
| A0A3B8JBJ8 | Ruminiclostridium sp |
| A0A3B8K2L7 | Firmicutes bacterium |
| A0A3B8K5S8 | Firmicutes bacterium |
| A0A3B8NMQ4 | Peptococcaceae bacterium |
| A0A3B8NQ33 | Peptococcaceae bacterium |
| A0A3B8SB28 | Lachnospiraceae bacterium |
| A0A3B9M453 | Peptococcaceae bacterium |
| A0A3B9PWM8 | Clostridiales bacterium UBA9856 |
| A0A3B9QBZ8 | Clostridiales bacterium UBA9857 |
| A0A3B9SPM9 | Peptococcaceae bacterium |
| A0A3B9SQ47 | Peptococcaceae bacterium |
| A0A3B9SSG2 | Desulfotomaculum sp |
| A0A3B9SZX9 | Ruminococcus sp |
| A0A3B9UZB9 | Clostridium sp. |
| A0A3C0D3A0 | Faecalibacterium sp |
| A0A3C0H8A7 | Firmicutes bacterium |
| A0A3C0IKT5 | Ruminococcus sp |
| A0A3C0J364 | Firmicutes bacterium |
| A0A3C0NNV8 | Clostridiales bacterium |
| A0A3C0SRU3 | Clostridium sp. |
| A0A3C0WAL9 | Oscillospiraceae bacterium |
| A0A3C0X2F4 | Oscillospiraceae bacterium |
| A0A3C1I6C7 | Ornithinibacillus sp |
| A0A3C1K354 | Clostridiales bacterium |
| A0A3C1LXN5 | Oscillospiraceae bacterium |
| A0A3C1QLW9 | Firmicutes bacterium |
| A0A3C1QM67 | Firmicutes bacterium |
| A0A3C2DWK9 | Oscillospiraceae bacterium |
| A0A3C2DX09 | Oscillospiraceae bacterium |
| A0A3C2EN61 | Clostridiales bacterium |
| A0A3D0E9Y1 | Bacillus sp. |
| A0A3D0M0Q5 | Oscillospiraceae bacterium |
| A0A3D0M2D9 | Oscillospiraceae bacterium |
| A0A3D0XR26 | Oscillospiraceae bacterium |
| A0A3D0YB63 | Clostridiales bacterium |
| A0A3D0Z0V5 | Clostridiales bacterium |
| A0A3D0Z4R0 | Oscillospiraceae bacterium |
| A0A3D1FEY1 | Firmicutes bacterium |
| A0A3D1HXL8 | Oscillospiraceae bacterium |
| A0A3D1HYL3 | Oscillospiraceae bacterium |
| A0A3D1HZW6 | Oscillospiraceae bacterium |
| A0A3D1JTZ2 | Clostridiales bacterium |
| A0A3D1LB76 | Clostridiales bacterium |
| A0A3D1Q831 | Syntrophomonas sp |
| A0A3D1QFN2 | Syntrophomonas sp |
| A0A3D1SZ61 | Firmicutes bacterium |
| A0A3D1VMT5 | Clostridiales bacterium |
| A0A3D1X695 | Oscillibacter sp |
| A0A3D1XC91 | Clostridiales bacterium |
| A0A3D2A8W4 | Oscillospiraceae bacterium |
| A0A3D2CLU3 | Clostridiales bacterium |
| A0A3D2FZK3 | Clostridiales bacterium |
| A0A3D2N3Y6 | Ruminococcus sp |
| A0A3D2Q295 | Desulfotomaculum sp |
| A0A3D2QHE0 | Oscillospiraceae bacterium |
| A0A3D2XCI8 | Lachnoclostridium phytofermentans |
| A0A3D3AG35 | Clostridiaceae bacterium |
| A0A3D3Y2G2 | Oscillospiraceae bacterium |
| A0A3D3Y3K9 | Oscillospiraceae bacterium |
| A0A3D4D8Z5 | Oscillibacter sp |
| A0A3D4EET5 | Clostridiales bacterium |
| A0A3D4FIA8 | Clostridium sp. |
| A0A3D4JLU0 | Ruminococcus sp |
| A0A3D4LJ30 | Clostridiales bacterium |
| A0A3D4MVC5 | Clostridiales bacterium |
| A0A3D4T6A9 | Oscillospiraceae bacterium |
| A0A3D4W0A2 | Faecalibacterium sp |
| A0A3D5LNG6 | Oscillospiraceae bacterium |
| A0A3D5MNI4 | Clostridiales bacterium |
| A0A3D5MVP4 | Clostridium sp. |
| A0A3D5TL14 | Oscillospiraceae bacterium |
| A0A3D5U1F3 | Bacillus sp. |
| A0A3D5UD81 | Clostridiales bacterium |
| A0A3D5VJD4 | Firmicutes bacterium |
| A0A3D5WU24 | Clostridiales bacterium |
| A0A3D6BH55 | Clostridiales bacterium |
| A0A3D8GTM4 | Bacillus piezotolerans |
| A0A3D8PHT3 | Oceanobacillus chungangensis |
| A0A3D8PLJ7 | Oceanobacillus arenosus |
| A0A3D8YTM7 | Sporosarcina sp. BI001-red |
| A0A3D9GUW2 | Paenibacillus sp. VMFN-D1 |
| A0A3D9I4L6 | Cohnella phaseoli |
| A0A3D9UYD7 | Bacillus mycoides |
| A0A3E0KJF7 | Firmicutes bacterium |
| A0A3E0R8V1 | Brevibacillus sp |
| A0A3E2B443 | Evtepia gabavorous |
| A0A3E2JQL9 | Bacillus sp. V59.32b |
| A0A3E2T3K5 | Harryflintia acetispora |
| A0A3E2TCZ2 | Faecalibacterium prausnitzii |
| A0A3E2TTR2 | Faecalibacterium prausnitzii |
| A0A3E2U976 | Faecalibacterium prausnitzii |
| A0A3E2V306 | Faecalibacterium prausnitzii |
| A0A3E2XB04 | Faecalibacterium prausnitzii |
| A0A3F2ZV25 | Clostridium botulinum (strain 657 / Type Ba4) |
| A0A3F3JUB5 | Faecalibacterium prausnitzii |
| A0A3F3S3X6 | Tissierella praeacuta |
| A0A3G1KTZ2 | Candidatus Formimonas warabiya |
| A0A3G2R5F0 | Biomaibacter acetigenes |
| A0A3G3K1F8 | Cohnella candidum |
| A0A3G5UIN2 | Bacillus sp. FDAARGOS_527 |
| A0A3L7JTA0 | Falsibacillus albus |
| A0A3M7TUW7 | Bacillus sp. KQ-3 |
| A0A3M8ALJ3 | Brevibacillus agri |
| A0A3M8B1I5 | Brevibacillus gelatini |
| A0A3M8C1D3 | Brevibacillus invocatus |
| A0A3M8C567 | Brevibacillus panacihumi |
| A0A3M8D8Z4 | Brevibacillus fluminis |
| A0A3M8D921 | Brevibacillus nitrificans |
| A0A3M8H7F6 | Lysinibacillus halotolerans |
| A0A3N1XR06 | Mobilisporobacter senegalensis |
| A0A3N5AWX6 | Thermodesulfitimonas autotrophica |
| A0A3N5B993 | Aquisalibacillus elongatus |
| A0A3N5ZQX6 | Rummeliibacillus sp. TYF005 |
| A0A3N9UI22 | Lysinibacillus composti |
| A0A3P1B841 | Bacillus pacificus |
| A0A3P1ZI32 | Desulfovibrio sp. OH1186_COT-070 |
| A0A3P5WVG9 | Filibacter tadaridae |
| A0A3Q9B4M5 | Halocella sp. SP3-1 |
| A0A3Q9HNW8 | Anoxybacter fermentans |
| A0A3Q9HUB1 | Anoxybacter fermentans |
| A0A3Q9I8I0 | Paenibacillus lutimineralis |
| A0A3Q9QW48 | Neobacillus mesonae |
| A0A3Q9SE28 | [Brevibacterium] frigoritolerans |
| A0A3R6P0K6 | Faecalibacterium sp. AF28-13AC |
| A0A3R9D996 | Bacillus sp. |
| A0A3R9EA12 | Mesobacillus subterraneus |
| A0A3S0HP48 | Lysinibacillus telephonicus |
| A0A3S0WAW7 | Peribacillus cavernae |
| A0A3S0YIS1 | Clostridium perfringens |
| A0A3S1D9W1 | Paenibacillus zeisoli |
| A0A3S1DQS3 | Paenibacillus anaericanus |
| A0A3S1EPZ1 | Bacillus sp. VKPM B-3276 |
| A0A3S4S661 | Bacillus freudenreichii |
| A0A3S6Z3G0 | Sulfobacillus thermotolerans |
| A0A3S8RQT2 | Paenibacillus lentus |
| A0A3S9T4D7 | Bacillus thuringiensis |
| A0A3T0KYT2 | Peribacillus asahii |
| A0A3T1D5T5 | Cohnella abietis |
| A0A401UQV4 | Clostridium tagluense |
| A0A402GM91 | Bacillus cereus |
| A0A410MUG7 | Lysinibacillus sphaericus |
| A0A410PLB1 | Clostridium sp. JN-9 |
| A0A410PVX8 | Aminipila sp. JN-18 |
| A0A412AXQ3 | [Clostridium] leptum |
| A0A413D9G4 | Faecalibacterium prausnitzii |
| A0A413G7T5 | Anaerotruncus sp. AF02-27 |
| A0A415E3I5 | Emergencia timonensis |
| A0A416R2V9 | Pseudoflavonifractor sp. AF19-9AC |
| A0A416Z6U4 | Ruminococcaceae bacterium AF10-16 |
| A0A417HNF5 | Ruminococcaceae bacterium AM28-23LB |
| A0A417N7R6 | Ruminococcaceae bacterium TF06-43 |
| A0A417UNK9 | Faecalibacterium sp. OM04-11BH |
| A0A417YD98 | Oceanobacillus profundus |
| A0A417YT94 | Neobacillus notoginsengisoli |
| A0A418IN22 | Staphylococcus xylosus |
| A0A418SZG8 | Paenibacillus sp. 1011MAR3C5 |
| A0A419G0M4 | Peptococcaceae bacterium |
| A0A419SQN1 | Ammoniphilus oxalaticus |
| A0A419T4I6 | Thermohalobacter berrensis |
| A0A419T4J7 | Thermohalobacter berrensis |
| A0A420H172 | Bacillus toyonensis |
| A0A424YCT7 | Candidatus Syntrophonatronum acetioxidans |
| A0A426H481 | Peribacillus simplex |
| A0A428J5Z4 | Bacillus sp. HMF5848 |
| A0A428M8Y9 | Herbinix hemicellulosilytica |
| A0A428MVW9 | Bacillus salarius |
| A0A429X2H0 | Bacillus terrae |
| A0A429Y6F5 | Bacillus acidinfaciens |
| A0A432LCA4 | Lysinibacillus antri |
| A0A437SAY1 | Bacillus thuringiensis |
| A0A443T7P7 | Bacillus mycoides |
| A0A446IE11 | Paeniclostridium sordellii 8483 |
| A0A450CL81 | Clostridioides difficile |
| A0A480BIT0 | Paenibacillus naphthalenovorans |
| A0A494WRZ0 | Desulfofundulus salinum |
| A0A494XL46 | Cohnella endophytica |
| A0A494YYE2 | Lysinibacillus endophyticus |
| A0A494Z6S2 | Oceanobacillus bengalensis |
| A0A495A7J9 | Oceanobacillus halophilus |
| A0A498CPN2 | Anaerotruncus sp. 22A2-44 |
| A0A498DJM8 | Oceanobacillus piezotolerans |
| A0A4D7AN98 | Dysosmobacter welbionis |
| A0A4E7PMH5 | Bacillus thuringiensis subsp. israelensis |
| A0A4P6B805 | Moorella sp. E306M |
| A0A4P6BAA4 | Moorella sp. E306M |
| A0A4P6HJ06 | Desulfovibrio carbinolicus |
| A0A4P6UTD4 | Ureibacillus thermophilus |
| A0A4P7A078 | Paenisporosarcina antarctica |
| A0A4P7GT46 | Thermaerobacter sp. FW80 |
| A0A4P8SCE3 | Lysinibacillus sp. SGAir0095 |
| A0A4P9F4I9 | Bacillus paranthracis |
| A0A4Q0I7B0 | Acetivibrio mesophilus |
| A0A4Q0V0X7 | Clostridium tetani |
| A0A4Q0VDB4 | Clostridium tetani |
| A0A4Q0VNV3 | Anaerobacillus alkaliphilus |
| A0A4Q1SZX3 | Ammoniphilus sp. CFH 90114 |
| A0A4Q7QAB4 | Fictibacillus sp. BK138 |
| A0A4Q9DRH6 | Paenibacillus thalictri |
| A0A4Q9YBD1 | Bacillus mycoides |
| A0A4Q9Z630 | Bacillus mycoides |
| A0A4R1B4W5 | Cytobacillus praedii |
| A0A4R1MSC2 | Natranaerovirga hydrolytica |
| A0A4R1QUX6 | Fournierella massiliensis |
| A0A4R2BDS4 | Mesobacillus foraminis |
| A0A4R2L4A5 | Marinisporobacter balticus |
| A0A4R2LZB3 | Flavonifractor plautii DSM 6740 |
| A0A4R2M644 | Flavonifractor plautii DSM 6740 |
| A0A4R2NZI0 | Scopulibacillus darangshiensis |
| A0A4R2P5G1 | Scopulibacillus darangshiensis |
| A0A4R2RTW7 | Heliophilum fasciatum |
| A0A4R2TW42 | Serpentinicella alkaliphila |
| A0A4R2XET2 | Bacillus sp. OK085 |
| A0A4R3KDC4 | Tepidibacillus fermentans |
| A0A4R3KYR5 | Keratinibaculum paraultunense |
| A0A4R3MJI7 | Natranaerovirga pectinivora |
| A0A4R3NC51 | Melghiribacillus thermohalophilus |
| A0A4R4BE05 | Bacillus thuringiensis |
| A0A4R4D1D2 | Dehalobacter sp. 12DCB1 |
| A0A4R4D2N7 | Dehalobacter sp. 12DCB1 |
| A0A4R4EDT6 | Paenibacillus sp. 18JY21-1 |
| A0A4R5KQ08 | Paenibacillus piri |
| A0A4R5VX74 | Bacillus salipaludis |
| A0A4R5XQ65 | Jeotgalibacillus sp. S-D1 |
| A0A4R5ZXS8 | Rhodococcus qingshengii |
| A0A4R6AIJ4 | [Brevibacterium] frigoritolerans |
| A0A4R7KU89 | Fonticella tunisiensis |
| A0A4R7UA66 | Lysinibacillus sp. YR326 |
| A0A4R8GK73 | Cytobacillus oceanisediminis |
| A0A4R8H065 | Orenia marismortui |
| A0A4S2DDX9 | Clostridium sartagoforme |
| A0A4S4BXB4 | Bacillus sp. DSL-17 |
| A0A4T2AA21 | Marinifilum sp. JC120 |
| A0A4T9WFH9 | Tissierella creatinini |
| A0A4U0F9B4 | Cohnella pontilimi |
| A0A4U1D3C9 | Bacillus kyonggiensis |
| A0A4U2MJ25 | Peribacillus simplex |
| A0A4U2NZ50 | Bacillus cereus |
| A0A4U2Q3W0 | Paenibacillus terrae |
| A0A4U2X7T3 | Bacillus mycoides |
| A0A4U2Y852 | Brevibacillus antibioticus |
| A0A4U2Z3T0 | Lysinibacillus mangiferihumi |
| A0A4U3BHM8 | Bacillus cereus |
| A0A4U8YN28 | Desulfoluna butyratoxydans |
| A0A4U9R496 | Hathewaya histolytica |
| A0A4V1S4J2 | Desulfotomaculum aquiferis |
| A0A4V2KLV9 | Lysinibacillus sp. OL1 |
| A0A4V2ZP85 | Zhaonella formicivorans |
| A0A4V3WZT5 | Bacillus sp. HUB-I-004 |
| A0A4V6ENS0 | Ruminiclostridium herbifermentans |
| A0A4V6RSY5 | Bacillus timonensis |
| A0A4Y3P861 | Brevibacillus parabrevis |
| A0A4Y6F4U6 | Bacillus tropicus |
| A0A4Y7QTW7 | Bacillus sp. BH2 |
| A0A4Y7REQ7 | Pelotomaculum schinkii |
| A0A4Y7RRP9 | Pelotomaculum propionicicum |
| A0A4Y7S1W0 | Pelotomaculum sp. FP |
| A0A4Y8IFE1 | Filobacillus milosensis |
| A0A4Y8UDC8 | [Brevibacterium] frigoritolerans |
| A0A4Y9AER3 | Lentibacillus salicampi |
| A0A4Z0QMV9 | Desulfosporosinus sp. Sb-LF |
| A0A4Z0QX40 | Desulfosporosinus sp. Sb-LF |
| A0A4Z0R2S4 | Desulfosporosinus fructosivorans |
| A0A4Z0Y062 | Caproiciproducens galactitolivorans |
| A0A501UKT3 | Clostridium perfringens |
| A0A502HIG4 | Brevibacillus laterosporus |
| A0A506Q1V4 | Bacillus sp. |
| A0A511BYY0 | Rummeliibacillus stabekisii |
| A0A511UWU4 | Cerasibacillus quisquiliarum |
| A0A511VZV5 | Alkalibacillus haloalkaliphilus |
| A0A511X1E2 | Halolactibacillus alkaliphilus |
| A0A511ZA79 | Sporosarcina luteola |
| A0A511ZD33 | Oceanobacillus sojae |
| A0A513RP23 | Bacillus sp. S3 |
| A0A514LJ53 | Salicibibacter halophilus |
| A0A516QLF1 | Bacillus sp. BD59S |
| A0A517DUM4 | Sporomusa termitida |
| A0A517I9J6 | Brevibacillus brevis |
| A0A518VA86 | Brevibacillus laterosporus |
| A0A521J7V3 | Gottschalkiaceae bacterium |
| A0A521JSA7 | Anaerolineaceae bacterium |
| A0A540V5M7 | Ureibacillus terrenus |
| A0A542ARC9 | Clostridium sp. KNHs216 |
| A0A542GVQ9 | Microbacterium sp. SLBN-1 |
| A0A542SIH3 | Brevibacillus sp. AG162 |
| A0A544SU94 | Psychrobacillus soli |
| A0A544TC41 | Psychrobacillus lasiicapitis |
| A0A544TTL3 | Psychrobacillus vulpis |
| A0A544UF81 | Lysinibacillus sp. SDF0037 |
| A0A544UY67 | Lysinibacillus sp. SDF0063 |
| A0A549YED7 | Lentibacillus cibarius |
| A0A553KS21 | Brevibacillus sp. LEMMJ03 |
| A0A556C5L6 | Bacillus sp. HY001 |
| A0A556PH08 | Allobacillus sp. SKP4-8 |
| A0A556PUB0 | Allobacillus sp. SKP2-8 |
| A0A559J9F4 | Cohnella sp. G13 |
| A0A561CY49 | [Brevibacterium] frigoritolerans |
| A0A561D8A3 | Neobacillus bataviensis |
| A0A561PHU5 | Paenibacillus sp. 597 |
| A0A562GSH5 | Sporomusa sp. KB1 |
| A0A562HD75 | Desulfitobacterium sp. LBE |
| A0A562HLS9 | Desulfitobacterium sp. LBE |
| A0A562JFS2 | Cytobacillus oceanisediminis |
| A0A562JKH7 | Sedimentibacter saalensis |
| A0A562QS94 | Alkalihalobacillus nanhaiisediminis |
| A0A564TJ22 | Faecalibacterium prausnitzii |
| A0A564UBG7 | Faecalibacterium prausnitzii |
| A0A5A9E5S4 | Bacillus sp. CH30_1T |
| A0A5B0CPV8 | Sporosarcina sp. ANT_H38 |
| A0A5B0WWK9 | Paenibacillus sp. B2(2019) |
| A0A5B7TJG4 | Caloramator sp. E03 |
| A0A5B8PCI2 | Bacillus cereus |
| A0A5B8ZE44 | Bacillus dafuensis |
| A0A5B9YJF4 | Cellulosilyticum sp. WCF-2 |
| A0A5C0SF84 | Crassaminicella sp. SY095 |
| A0A5C1F943 | Bacillus sp. JAS24-2 |
| A0A5C1FPL0 | Bacillus mycoides |
| A0A5C4T074 | Paenibacillus hemerocallicola |
| A0A5C4ZHZ6 | Bacillus pacificus |
| A0A5C5A368 | Bacillus tropicus |
| A0A5C5AKT7 | Bacillus sp. CD3-5 |
| A0A5C6W520 | Metabacillus litoralis |
| A0A5D0CN84 | Paenibacillus faecis |
| A0A5D4KDH0 | Rossellomorea vietnamensis |
| A0A5D4KXV8 | Bacillus megaterium |
| A0A5D4M9V1 | Rossellomorea vietnamensis |
| A0A5D4NX00 | Rossellomorea vietnamensis |
| A0A5D4R9H1 | Bacillus infantis |
| A0A5D4S2R5 | Bacillus marisflavi |
| A0A5D4SPQ7 | Bacillus infantis |
| A0A5D4T384 | Bacillus horikoshii |
| A0A5D4T3R3 | Bacillus horikoshii |
| A0A5D4TUM1 | Bacillus aquimaris |
| A0A5D4TVK0 | Bacillus aquimaris |
| A0A5E9IIN0 | Bacillus thuringiensis F14-1 |
| A0A5J5H1Q3 | Bacillus endozanthoxylicus |
| A0A5J6PNV1 | Psychrobacillus sp. AK 1817 |
| A0A5J6SJ47 | Psychrobacillus glaciei |
| A0A5K5IH84 | Bacillus sp. FDAARGOS_235 |
| A0A5M8T9G6 | Bacillus cereus |
| A0A5M9GRJ1 | Bacillus paranthracis |
| A0A5M9NFY2 | Clostridium sp. HV4-5-A1G |
| A0A5P0YGD3 | Fictibacillus phosphorivorans |
| A0A5P3XFA4 | Paraclostridium bifermentans |
| A0A5P9HUS3 | Bacillus sp. THAF10 |
| A0A5P9XX08 | Bacillus cereus |
| A0A5Q2N8S1 | Lysinibacillus pakistanensis |
| A0A5Q2NA92 | Heliorestis convoluta |
| A0A5Q2TFM6 | Gracilibacillus sp. SCU50 |
| A0A5R9FDP0 | Alkalihalobacillus caeni |
| A0A5R9GE95 | Paenibacillus antri |
| A0A5R9MKS4 | Ruminococcus sp. KGMB03662 |
| A0A5S4ZU51 | Desulfallas thermosapovorans DSM 6562 |
| A0A5S5B0G2 | Thermosediminibacter litoriperuensis |
| A0A5S5CLE8 | Paenibacillus methanolicus |
| A0A644SYL7 | bioreactor metagenome |
| A0A644TWX1 | bioreactor metagenome |
| A0A644VJA4 | bioreactor metagenome |
| A0A644W404 | bioreactor metagenome |
| A0A644WWD5 | bioreactor metagenome |
| A0A644YQ45 | bioreactor metagenome |
| A0A645A740 | bioreactor metagenome |
| A0A645AAP8 | bioreactor metagenome |
| A0A645ABM7 | bioreactor metagenome |
| A0A645BWR7 | bioreactor metagenome |
| A0A653T5I2 | Bacillus sp. 349Y |
| A0A653VYQ2 | Bacillus mycoides |
| A0A657P3X8 | Bacillus cereus |
| A0A658J9E0 | Butyricicoccus sp. 1XD8-22 |
| A0A679H561 | Bacillus wiedmannii |
| A0A6A2T2F5 | Bacillus sp. B3-WWTP-C-10-D-3 |
| A0A6A4VE12 | Amphibalanus amphitrite |
| A0A6A8DII4 | Aquibacillus halophilus |
| A0A6A8KEA7 | Faecalibacterium prausnitzii |
| A0A6A8SI29 | Turicibacter sanguinis |
| A0A6A8V818 | Pseudoflavonifractor sp. BIOML-A6 |
| A0A6A8V830 | Pseudoflavonifractor sp. BIOML-A6 |
| A0A6B1UB15 | Bittarella massiliensis |
| A0A6B3TQF1 | Neobacillus thermocopriae |
| A0A6B3VUV5 | Bacillus aquiflavi |
| A0A6B3WAP4 | Clostridium botulinum |
| A0A6B3YFS9 | Clostridium botulinum |
| A0A6B3YNZ8 | Clostridium botulinum |
| A0A6B4A337 | Clostridium botulinum |
| A0A6B4GWU8 | Clostridium botulinum |
| A0A6B4HVR8 | Clostridium botulinum |
| A0A6B4IBA8 | Clostridium botulinum |
| A0A6B4JR13 | Clostridium botulinum |
| A0A6B4NDX9 | Clostridium botulinum |
| A0A6B4R0J7 | Clostridium botulinum |
| A0A6B4RTY4 | Clostridium botulinum |
| A0A6B4SA94 | Clostridium botulinum |
| A0A6B4VHG7 | Clostridium botulinum |
| A0A6B4YFX7 | Clostridium botulinum |
| A0A6B4ZZP1 | Clostridium botulinum |
| A0A6B8K598 | Bacillus sp. N3536 |
| A0A6B9YG48 | Virgibacillus sp. MSP4-1 |
| A0A6C0FZY1 | Paenibacillus lycopersici |
| A0A6D1SXL4 | Bacillus sp. BH32 |
| A0A6G1X5J6 | Salinibacillus xinjiangensis |
| A0A6G2CJC6 | Turicibacter sanguinis |
| A0A6G3LZ91 | Pseudoflavonifractor sp. 60 |
| A0A6G4Z9R8 | Clostridium perfringens |
| A0A6H0TKF9 | Bacillus thuringiensis serovar andalousiensis |
| A0A6H1NX95 | Bacillus megaterium |
| A0A6H1X3M4 | Romboutsia sp. CE17 |
| A0A6H3AAH2 | Bacillus anthracis |
| A0A6H9IE09 | Bacillus sp. AY1-10 |
| A0A6H9IPI7 | Bacillus sp. BPN334 |
| A0A6H9JK55 | Bacillus sp. AY2-1 |
| A0A6I0BHK4 | Bacillus sp. CH126_4D |
| A0A6I0F2A0 | Heliorestis acidaminivorans |
| A0A6I0FLR3 | Alkaliphilus pronyensis |
| A0A6I1FGY3 | Bacillus aerolatus |
| A0A6I2AHC9 | Bacillus thuringiensis |
| A0A6I2ETP7 | Bacillus thuringiensis |
| A0A6I2M920 | Bacillus idriensis |
| A0A6I2RNR0 | Flavonifractor plautii |
| A0A6I3Q644 | Ruthenibacterium lactatiformans |
| A0A6I3R4S9 | Pseudoflavonifractor sp. BIOML-A4 |
| A0A6I3RBZ7 | Pseudoflavonifractor sp. BIOML-A4 |
| A0A6I5ZLZ1 | Moorella glycerini |
| A0A6I5ZUP3 | Moorella glycerini |
| A0A6I6DHN4 | Candidatus Syntrophocurvum alkaliphilum |
| A0A6I6DMY8 | Candidatus Syntrophocurvum alkaliphilum |
| A0A6I6F0W7 | Clostridium bovifaecis |
| A0A6I6PVT3 | Bacillus marisflavi |
| A0A6I6UPJ5 | Rossellomorea vietnamensis |
| A0A6I6YBY6 | Bacillus paranthracis |
| A0A6I7FRP3 | Bacillus sp. NSP2.1 |
| A0A6I7GP76 | Flavonifractor plautii |
| A0A6I7XUL2 | Bacillus sp. SH7-1 |
| A0A6L3BP21 | Bacillus sp. TE8-1 |
| A0A6L3BXH4 | Bacillus sp. BB081 |
| A0A6L3V7K7 | Cytobacillus depressus |
| A0A6L3WQK2 | Bacillus cereus |
| A0A6L5B0U8 | Bacillus sp. ZZV12-4809 |
| A0A6L5LFR6 | Bacillus thuringiensis |
| A0A6L5PX38 | Bacillus sp. RIT694 |
| A0A6L6LRT4 | Ruthenibacterium lactatiformans |
| A0A6L8JRB9 | Virgibacillus halodenitrificans |
| A0A6L8P0Y2 | Bacillus anthracis |
| A0A6M0GZ06 | Clostridium senegalense |
| A0A6M0Q472 | Bacillus mesophilus |
| A0A6M0R927 | Clostridium niameyense |
| A0A6M0SVI1 | Clostridium botulinum |
| A0A6M0XFW4 | Clostridium botulinum |
| A0A6M0XZF1 | Clostridium sporogenes |
| A0A6M0YNS1 | Clostridium botulinum |
| A0A6M1VSI4 | Clostridium perfringens |
| A0A6M1WJW8 | Clostridium perfringens |
| A0A6M8DFM4 | Bacillus cereus |
| A0A6M8GS97 | Arthrobacter citreus |
| A0A6N2HHA1 | Bacillus sp. AR8-1 |
| A0A6N2V9I7 | uncultured Anaerotruncus sp |
| A0A6N3AVX5 | uncultured Clostridium sp |
| A0A6N3BM97 | Clostridium paraputrificum |
| A0A6N3CGG9 | uncultured Clostridium sp |
| A0A6N3D3J9 | Intestinibacter bartlettii |
| A0A6N3D3M1 | Flavonifractor plautii |
| A0A6N3GUU3 | Clostridium tertium |
| A0A6N7AYE7 | Firmicutes bacterium |
| A0A6N7B269 | Firmicutes bacterium |
| A0A6N7ISZ7 | Desulfofundulus thermobenzoicus |
| A0A6N7R0Z7 | Gracilibacillus thailandensis |
| A0A6N7TN75 | Faecalibacterium sp. BIOML-A1 |
| A0A6N7XNX5 | Tissierella pigra |
| A0A6N8B4R0 | Firmicutes bacterium |
| A0A6N8BDN2 | Firmicutes bacterium |
| A0A6N8BEI8 | Firmicutes bacterium |
| A0A6N8BNZ2 | Desulfovibrio sp |
| A0A6N8BU81 | Clostridiaceae bacterium |
| A0A6N8C219 | Clostridiaceae bacterium |
| A0A6N8CTT3 | Terrilactibacillus tamarindi |
| A0A6N8FIJ6 | Ornithinibacillus caprae |
| A0A6N8HS02 | Lentibacillus sp. JNUCC-1 |
| A0A6N9PSR5 | Neglecta sp. 59 |
| A0A6N9QHI8 | Clostridiales bacterium |
| A0A6P1AUE7 | bacterium LRH843 |
| A0A6P1HH38 | Pontibacillus sp. HMF3514 |
| A0A6P1MJ93 | Aminipila sp. CBA3637 |
| A0A6P1YC00 | Caloranaerobacter azorensis |
| A0A6S6XN32 | Ruminococcaceae bacterium BL-6 |
| A0A6S6YDF0 | Ruminococcaceae bacterium BL-4 |
| A0A7C1K7S8 | Firmicutes bacterium |
| A0A7C6CL76 | Firmicutes bacterium |
| A0A7C6DDK9 | Clostridiales bacterium |
| A0A7C6DDZ0 | Clostridiaceae bacterium |
| A0A7C6GDH1 | Firmicutes bacterium |
| A0A7C6GNF6 | Firmicutes bacterium |
| A0A7C6GQS9 | Clostridiales bacterium |
| A0A7C6H0R5 | Firmicutes bacterium |
| A0A7C6HBU7 | Firmicutes bacterium |
| A0A7C6I248 | bacterium |
| A0A7C6I7L3 | Clostridia bacterium |
| A0A7C6J2V5 | Firmicutes bacterium |
| A0A7C6JG03 | Halanaerobiaceae bacterium |
| A0A7C6JTG4 | Firmicutes bacterium |
| A0A7C6K588 | Tissierellia bacterium |
| A0A7C6KMN9 | Firmicutes bacterium |
| A0A7C6KMV2 | Firmicutes bacterium |
| A0A7C6KQF9 | Firmicutes bacterium |
| A0A7C6KQL7 | Peptococcaceae bacterium |
| A0A7C6KT62 | Peptococcaceae bacterium |
| A0A7C6KVP1 | Clostridia bacterium |
| A0A7C6LA05 | Firmicutes bacterium |
| A0A7C6LGA3 | Firmicutes bacterium |
| A0A7C6LMA9 | Syntrophomonadaceae bacterium |
| A0A7C6LXM1 | Clostridiales bacterium |
| A0A7C6MQM7 | Natronincola sp |
| A0A7C6MWU2 | Clostridiales bacterium |
| A0A7C6MWY4 | Thermoanaerobacterales bacterium |
| A0A7C6N070 | Clostridiales bacterium |
| A0A7C6NBF0 | Peptococcaceae bacterium |
| A0A7C6PA07 | Syntrophomonadaceae bacterium |
| A0A7C6PC58 | Syntrophomonadaceae bacterium |
| A0A7C6QL67 | Syntrophomonadaceae bacterium |
| A0A7C6QMM1 | Firmicutes bacterium |
| A0A7C6QZ05 | Syntrophomonadaceae bacterium |
| A0A7C6R2F0 | Desulfotomaculum sp |
| A0A7C6R779 | Clostridiales bacterium |
| A0A7C6RBJ1 | Firmicutes bacterium |
| A0A7C6S8L4 | Firmicutes bacterium |
| A0A7C6UCY2 | Firmicutes bacterium |
| A0A7C6UP72 | Clostridia bacterium |
| A0A7C6UQ56 | Firmicutes bacterium |
| A0A7C6UX56 | Clostridiaceae bacterium |
| A0A7C6V1S1 | Syntrophomonadaceae bacterium |
| A0A7C6V2J3 | Syntrophomonadaceae bacterium |
| A0A7C6VSC9 | Epulopiscium sp |
| A0A7C6VV10 | Firmicutes bacterium |
| A0A7C6WKY1 | Clostridiales bacterium |
| A0A7C6WXJ4 | Thermoanaerobacterales bacterium |
| A0A7C6X1Y6 | Firmicutes bacterium |
| A0A7C6X4B9 | Firmicutes bacterium |
| A0A7C6XXJ1 | Clostridiales bacterium |
| A0A7C6Y7E3 | Firmicutes bacterium |
| A0A7C6YKV7 | Thermoanaerobacterales bacterium |
| A0A7C6YXI0 | Firmicutes bacterium |
| A0A7C6ZDK9 | Firmicutes bacterium |
| A0A7C6ZFI6 | Firmicutes bacterium |
| A0A7C6ZH21 | Clostridia bacterium |
| A0A7C6ZIJ8 | Clostridia bacterium |
| A0A7C6ZKX3 | Syntrophaceticus sp |
| A0A7C6ZKZ4 | Syntrophaceticus sp |
| A0A7C6ZPG7 | Firmicutes bacterium |
| A0A7C7AH63 | Tissierellia bacterium |
| A0A7C7D3B7 | Desulfitobacterium dehalogenans |
| A0A7C7D8F6 | Desulfitobacterium dehalogenans |
| A0A7C7DDM2 | Firmicutes bacterium |
| A0A7C7E6Q8 | Clostridiales bacterium |
| A0A7C7E9Q5 | Thermoanaerobacterales bacterium |
| A0A7C7EEY8 | Clostridiales bacterium |
| A0A7C7ES76 | Tissierellia bacterium |
| A0A7C8HEL4 | Defluviitalea raffinosedens |
| A0A7C9H7Q3 | Firmicutes bacterium |
| A0A7C9LNW5 | Firmicutes bacterium |
| A0A7D3YTS7 | Bacillus cereus |
| A0A7D6W0W0 | Clostridium intestinale |
| A0A7G6DYX6 | Thermoanaerosceptrum fracticalcis |
| A0A7G7FZN6 | Metabacillus sp. KUDC1714 |
| A0A7G8U143 | Brevibacterium sp. PAMC23299 |
| A0A7G8UM29 | Paenibacillus sp. PAMC21692 |
| A0A7G8X521 | Sporosarcina sp. resist |
| A0A7G9B1C0 | Oscillibacter sp. NSJ-62 |
| A0A7G9WA91 | Alkalicella caledoniensis |
| A0A7H8S7I8 | Lentibacillus sp. CBA3610 |
| A0A7H8SBS3 | Lentibacillus sp. CBA3610 |
| A0A7I8CZX6 | Solibaculum mannosilyticum |
| A0A7M1PWT5 | Clostridium sp. 'deep sea' |
| A0A7M1SCY3 | Bacillus sp. HD4P25 |
| A0A7M2AUG6 | Brevibacillus sp. JNUCC-41 |
| A0A7M2B8L1 | Brevibacterium sp. JNUCC-42 |
| A0A7M2QY21 | Viridibacillus sp. JNUCC-6 |
| A0A7S7J0H7 | Clostridiales bacterium |
| A0A7S7L5L3 | Anaerobacillus isosaccharinicus |
| A0A7T0GK46 | Sarcina sp. JB2 |
| A0A7T2CM09 | Lysinibacillus sp. JNUCC-51 |
| A0A7T2CWN8 | Lysinibacillus sp. JNUCC-52 |
| A0A7T3MQN5 | Bacillus thuringiensis |
| A0A7T5JMM6 | Brevibacillus sp. FJAT-54423 |
| A0A7T6Z6I6 | Salicibibacter cibarius |
| A0A7T6ZDJ5 | Salicibibacter cibi |
| A0A7T8FS40 | Lysinibacillus sp. FJAT-51161 |
| A0A7T8LT30 | Bacillus sp. TK-2 |
| A0A7T9LNC4 | Weizmannia coagulans |
| A0A7T9M1K1 | Peribacillus psychrosaccharolyticus |
| A0A7T9WWL7 | Bacillus cereus |
| A0A7U1C0Y7 | Bacillus vini |
| A0A7U1GM15 | Bacillus oleronius |
| A0A7U5GXH5 | Sporosarcina sp. P37 |
| A0A7U6BS14 | Peribacillus butanolivorans |
| A0A7U6BYI3 | Bacillus anthracis |
| A0A7U6KG44 | Lachnospiraceae bacterium KM106-2 |
| A0A7U9C9P3 | Clostridium sporogenes (strain ATCC 7955 / DSM 767 / NBRC 16411 / NCIMB 8053 / NCTC 8594 / PA 3679) |
| A0A7U9IA45 | Clostridium sp. (strain ATCC 29733 / VPI C48-50) |
| A0A7V3T2L0 | Clostridia bacterium |
| A0A7V6GCS0 | Clostridia bacterium |
| A0A7V6HMG2 | Bacilli bacterium |
| A0A7V6JHG5 | Clostridiaceae bacterium |
| A0A7V6M949 | bacterium |
| A0A7V6MTU2 | Thermoanaerobacterales bacterium |
| A0A7V6MY74 | Thermoanaerobacterales bacterium |
| A0A7V6PWL0 | Clostridiaceae bacterium |
| A0A7V6QRQ9 | Bacillales bacterium |
| A0A7V6RKU2 | Syntrophomonadaceae bacterium |
| A0A7V6RN39 | Syntrophomonadaceae bacterium |
| A0A7V6TLZ4 | Tissierellia bacterium |
| A0A7V6TV71 | Clostridiaceae bacterium |
| A0A7V6UD60 | Thermoanaerobacterales bacterium |
| A0A7V6UY66 | Clostridiaceae bacterium |
| A0A7V6X306 | Clostridia bacterium |
| A0A7V6Y6I5 | Thermoanaerobacterales bacterium |
| A0A7V6YDU2 | Clostridia bacterium |
| A0A7V6ZW88 | Clostridia bacterium |
| A0A7V7BGU3 | Clostridia bacterium |
| A0A7V7BLQ0 | Bacillus sp. |
| A0A7V7HHU0 | Bacillus sp. AR2-1 |
| A0A7V7HIR9 | Bacillus sp. SH5-2 |
| A0A7V7IBB4 | Bacillus sp. BB56-3 |
| A0A7V7L1U8 | Bacillus sp. BF2-3 |
| A0A7V7LCJ6 | Bacillus sp. BB51/4 |
| A0A7V7V767 | Bacillus luti |
| A0A7V7YXD2 | Bacillus sp. B1-WWTP-T-0.5-Post-4 |
| A0A7V8ICA6 | Clostridium sp. NCR |
| A0A7W0HLA7 | Desulfosalsimonas propionicica |
| A0A7W3SPF6 | Fontibacillus solani |
| A0A7W4L921 | Bacillus sp. APMAM |
| A0A7W5B4Z0 | Paenibacillus phyllosphaerae |
| A0A7W7X7L1 | Bacillus toyonensis |
| A0A7X0HT98 | Bacillus benzoevorans |
| A0A7X0RQH6 | Cohnella nanjingensis |
| A0A7X0S9B5 | Clostridium gasigenes |
| A0A7X0VL37 | Clostridium algidicarnis |
| A0A7X1IC48 | Bittarella massiliensis |
| A0A7X2AYS0 | Bacillus thuringiensis |
| A0A7X2DN79 | Bacillus thuringiensis |
| A0A7X2EBL1 | Bacillus thuringiensis |
| A0A7X2IWM3 | Bacillus lacus |
| A0A7X2JV26 | Bacillus thuringiensis |
| A0A7X2MZ49 | Inconstantimicrobium porci |
| A0A7X2Z2I4 | Paenibacillus woosongensis |
| A0A7X3DJR4 | Oscillibacter sp |
| A0A7X3IEC6 | Paenibacillus sp. HJL G12 |
| A0A7X5PCB6 | Clostridium sporogenes |
| A0A7X6SBF8 | Tissierellia bacterium |
| A0A7X6UIS6 | Syntrophomonadaceae bacterium |
| A0A7X6UIZ4 | Syntrophomonadaceae bacterium |
| A0A7X6VD94 | Clostridiaceae bacterium |
| A0A7X6VI85 | Clostridiaceae bacterium |
| A0A7X6VSZ7 | Syntrophomonadaceae bacterium |
| A0A7X6WLS9 | Syntrophomonadaceae bacterium |
| A0A7X6XUD8 | Thermoanaerobacterales bacterium |
| A0A7X6Y3N0 | Clostridiaceae bacterium |
| A0A7X6Y8B8 | Clostridia bacterium |
| A0A7X7AP46 | Clostridia bacterium |
| A0A7X7CH54 | Clostridiaceae bacterium |
| A0A7X7EJ03 | Clostridiaceae bacterium |
| A0A7X7GZ90 | Bacilli bacterium |
| A0A7X7MUM9 | Clostridia bacterium |
| A0A7X7MVJ8 | Clostridia bacterium |
| A0A7X7NZ92 | Syntrophomonadaceae bacterium |
| A0A7X7Q1I2 | Bacilli bacterium |
| A0A7X7R440 | Syntrophomonadaceae bacterium |
| A0A7X7R4F8 | Syntrophomonadaceae bacterium |
| A0A7X7RDS6 | Clostridia bacterium |
| A0A7X7S9Z9 | Syntrophomonadaceae bacterium |
| A0A7X7SXE4 | Clostridiaceae bacterium |
| A0A7X7TKW1 | Syntrophomonadaceae bacterium |
| A0A7X7VXL3 | Clostridiaceae bacterium |
| A0A7X7W023 | Clostridiaceae bacterium |
| A0A7X7X2I2 | Clostridiaceae bacterium |
| A0A7X7XYV0 | Clostridium sp. |
| A0A7X7Y1U9 | Tissierellia bacterium |
| A0A7X7YMR1 | Syntrophomonadaceae bacterium |
| A0A7X8ATQ3 | Clostridium sp. |
| A0A7X8AXV7 | Bacilli bacterium |
| A0A7X8DGT4 | Peptococcaceae bacterium |
| A0A7X8DRN7 | Halanaerobiaceae bacterium |
| A0A7X8E5I6 | Tissierellia bacterium |
| A0A7X8EB61 | Clostridia bacterium |
| A0A7X8EQQ8 | Epulopiscium sp |
| A0A7X8G314 | Tissierellia bacterium |
| A0A7X8HVB8 | Epulopiscium sp |
| A0A7X8ILD3 | Syntrophomonadaceae bacterium |
| A0A7X8IYI9 | Tissierellia bacterium |
| A0A7X8J0G8 | Clostridiaceae bacterium |
| A0A7X8JGE8 | Clostridiaceae bacterium |
| A0A7X8JPL1 | Epulopiscium sp |
| A0A7X8JV28 | Clostridiaceae bacterium |
| A0A7X8JVM6 | Clostridiaceae bacterium |
| A0A7X8K0M5 | Clostridia bacterium |
| A0A7X8KU57 | Peptococcaceae bacterium |
| A0A7X8KVQ6 | Peptococcaceae bacterium |
| A0A7X8KXH3 | Tissierellia bacterium |
| A0A7X8LBC6 | Epulopiscium sp |
| A0A7X8LE64 | Tissierellia bacterium |
| A0A7X8LKM9 | Syntrophomonadaceae bacterium |
| A0A7X8LLI0 | Syntrophomonadaceae bacterium |
| A0A7X8LZ60 | Clostridiaceae bacterium |
| A0A7X8M5S1 | Clostridiaceae bacterium |
| A0A7X8ME38 | Clostridium sp. |
| A0A7X8MKV5 | Syntrophomonadaceae bacterium |
| A0A7X8MKW5 | Syntrophomonadaceae bacterium |
| A0A7X8MTC7 | Clostridia bacterium |
| A0A7X8QKK6 | Clostridiaceae bacterium |
| A0A7X8QZ39 | Clostridiaceae bacterium |
| A0A7X8RI82 | Bacillus sp. RO1 |
| A0A7X8U432 | Syntrophomonadaceae bacterium |
| A0A7X8U9N5 | Syntrophomonadaceae bacterium |
| A0A7X8UAT8 | Syntrophomonadaceae bacterium |
| A0A7X8UAU5 | Syntrophomonadaceae bacterium |
| A0A7X8V2N3 | Clostridia bacterium |
| A0A7X8V4D6 | Clostridia bacterium |
| A0A7X8VE48 | Thermoanaerobacteraceae bacterium |
| A0A7X8X4U8 | Clostridia bacterium |
| A0A7X8YPE3 | Syntrophomonadaceae bacterium |
| A0A7X9A2W8 | Clostridia bacterium |
| A0A7X9A3N7 | Clostridia bacterium |
| A0A7X9AK19 | Clostridiaceae bacterium |
| A0A7X9BDX9 | Peptococcaceae bacterium |
| A0A7X9BN40 | Syntrophomonadaceae bacterium |
| A0A7X9BY17 | Desulfitobacterium sp |
| A0A7X9BYB6 | Desulfitobacterium sp |
| A0A7X9CMI0 | Tissierellia bacterium |
| A0A7X9GIJ8 | Syntrophomonadaceae bacterium |
| A0A7X9GX79 | Tissierellia bacterium |
| A0A7X9H752 | Clostridium sp. |
| A0A7X9HV19 | Clostridiaceae bacterium |
| A0A7X9M2Y9 | Bacillus sp. DNRA2 |
| A0A7X9MUW7 | Psychrobacillus sp. BL-248-WT-3 |
| A0A7X9XEY1 | Clostridium sp. SM-530-WT-3G |
| A0A7Y0HRM8 | Clostridium sp. P21 |
| A0A7Y0L2Y7 | Sulfobacillus sp. DSM 109850 |
| A0A7Y0QFI2 | Clostridioides difficile |
| A0A7Y0R3C4 | Clostridioides difficile |
| A0A7Y3V7F3 | Clostridium cochlearium |
| A0A7Y8S572 | Bacillus sp. |
| A0A7Y9BFJ1 | Bacillus sp. EB106-08-02-XG196 |
| A0A7Z0PMB4 | Bacillus sp. Gen3 |
| A0A7Z1FW67 | Bacillus pseudomycoides |
| A0A7Z1G0F8 | Bacillus pseudomycoides |
| A0A7Z1H6N7 | Bacillus pseudomycoides |
| A0A7Z8GW09 | Bacillus sp. 007/AIA-02/001 |
| A0A7Z8RK23 | Bacillus sp. AY18-3 |
| A0A7Z8RWA2 | Bacillus sp. AR13-1 |
| A0A7Z8S410 | Bacillus sp. BF9-10 |
| A0A800N994 | Cytobacillus firmus |
| A0A806I8Q0 | Bacillus thuringiensis HD-789 |
| A0A806LMP8 | Lysinibacillus varians |
| A0A806Q4X9 | Clostridium botulinum CDC_297 |
| A0A810Q5T9 | Oscillibacter sp. MM59 |
| A0A822Q2Z0 | Paeniclostridium sordellii |
| A0A822ULY4 | Clostridium perfringens |
| A0A826HM10 | Bacillus thuringiensis Bt407 |
| A0A828S1L8 | Turicibacter sp. HGF1 |
| A0A828XKZ7 | Bacillus cereus BAG4X12-1 |
| A0A828ZDY2 | Lysinibacillus fusiformis ZB2 |
| A0A829ZSL2 | Thermanaeromonas sp. C210 |
| A0A829ZUL2 | Thermanaeromonas sp. C210 |
| A0A833HN03 | Alkaliphilus serpentinus |
| A0A837GI25 | Bacillaceae bacterium MTCC 10057 |
| A0A837KMQ6 | Brevibacillus formosus |
| A0A837YUD3 | Bacillus badius |
| A0A838X3K7 | Brevibacillus halotolerans |
| A0A839TQS9 | Paenibacillus rhizosphaerae |
| A0A840KLU9 | Sporosarcina luteola |
| A0A840PPP3 | Ureibacillus thermosphaericus |
| A0A840QSY3 | Texcoconibacillus texcoconensis |
| A0A841KV83 | Anaerosolibacter carboniphilus |
| A0A841Q0X3 | Geomicrobium halophilum |
| A0A841Q651 | Salirhabdus euzebyi |
| A0A844DH03 | Faecalibacterium prausnitzii |
| A0A844DYE9 | Faecalibacterium prausnitzii |
| A0A844E9W1 | Faecalibacterium sp. BIOML-A3 |
| A0A844FHR5 | Anaerosalibacter bizertensis |
| A0A844K0X8 | Pseudoflavonifractor sp. BIOML-A18 |
| A0A844K2L4 | Pseudoflavonifractor sp. BIOML-A18 |
| A0A844LWF0 | Virgibacillus dakarensis |
| A0A845QQR3 | Anaerotruncus colihominis |
| A0A845QT30 | Senegalia massiliensis |
| A0A845R4V3 | Colidextribacter sp. OB.20 |
| A0A845RA57 | Colidextribacter sp. OB.20 |
| A0A845RL93 | Anaerotruncus colihominis |
| A0A845RRR7 | Dehalobacter sp. 4CP |
| A0A845RT85 | Dehalobacter sp. 4CP |
| A0A846HS47 | Clostridium botulinum |
| A0A846J4Y7 | Clostridium botulinum |
| A0A846JZ08 | Clostridium botulinum |
| A0A846TKE1 | Mesobacillus selenatarsenatis |
| A0A847AWZ6 | Tissierellia bacterium |
| A0A847CA00 | Oscillospiraceae bacterium |
| A0A847KCU1 | Peptococcaceae bacterium |
| A0A847NAY7 | Gracilibacteraceae bacterium |
| A0A847NCY6 | Gracilibacteraceae bacterium |
| A0A847NMB1 | Tissierellia bacterium |
| A0A847QJ83 | Veillonellaceae bacterium |
| A0A847QK74 | Peptococcaceae bacterium |
| A0A847QQQ4 | Peptococcaceae bacterium |
| A0A847QRM5 | Epulopiscium sp |
| A0A847WA91 | Papillibacter sp |
| A0A847WEB3 | Epulopiscium sp |
| A0A847XCR4 | Tissierellia bacterium |
| A0A848BLW0 | Paraclostridium bifermentans |
| A0A850EN10 | Paenibacillus sp. JW14 |
| A0A852TDM5 | Neobacillus niacini |
| A0A852UDW1 | Sporosarcina sp. JAI121 |
| A0A853WAF5 | Moorella thermoacetica |
| A0A853XD93 | Bacillus sp. L27 |
| A0A853XNP5 | Bacillus pacificus |
| A0A853Y7D0 | Bacillus mobilis |
| A0A854AZS9 | Bacillus toyonensis |
| A0A854DGV6 | Bacillus thuringiensis |
| A0A854KQ33 | Bacillus thuringiensis serovar shandongiensis |
| A0A854U9Y6 | Peribacillus simplex |
| A0A855AQQ2 | Bacillus thuringiensis |
| A0A855AUN2 | Bacillus cereus |
| A0A855BI38 | Bacillus cereus |
| A0A855E4Z1 | Bacillus thuringiensis |
| A0A855KY33 | Bacillus sp. AKBS9 |
| A0A857DDP5 | Dehalobacter restrictus |
| A0A857DMW9 | Dehalobacter restrictus |
| A0A858BUP0 | Aminipila butyrica |
| A0A858BWL1 | Aminipila butyrica |
| A0A8A3NRN9 | thermophilic bacterium 3443-3Ac |
| A0A8A5Z6E9 | Cohnella sp. LGH |
| A0A8A7KNN4 | Halanaerobiaceae bacterium NS-1 |
| A0A8A9G9B2 | Bacillus cytotoxicus |
| A0A8B2VMS5 | Bacillus sp. dmp5 |
| A0A8B4BZD4 | Bacillus coagulans DSM 1 = ATCC 7050 |
| A0A8B5XTP7 | Peribacillus simplex |
| A0A8B6J9B3 | Clostridioides difficile |
| A0PYH5 | Clostridium novyi (strain NT) |
| A0R959 | Bacillus thuringiensis (strain Al Hakam) |
| A1HS32 | Thermosinus carboxydivorans Nor1 |
| A3DCJ3 | Acetivibrio thermocellus (strain ATCC 27405 / DSM 1237 / JCM 9322 / NBRC 103400 / NCIMB 10682 / NRRL B-4536 / VPI 7372) |
| A4J8C7 | Desulfotomaculum reducens (strain MI-1) |
| A5D4E2 | Pelotomaculum thermopropionicum (strain DSM 13744 / JCM 10971 / SI) |
| A5D4H5 | Pelotomaculum thermopropionicum (strain DSM 13744 / JCM 10971 / SI) |
| A5I764 | Clostridium botulinum (strain Hall / ATCC 3502 / NCTC 13319 / Type A) |
| A6CL50 | Bacillus sp. SG-1 |
| A6P157 | Pseudoflavonifractor capillosus ATCC 29799 |
| A6P165 | Pseudoflavonifractor capillosus ATCC 29799 |
| A6TSS7 | Alkaliphilus metalliredigens (strain QYMF) |
| A7GIS6 | Clostridium botulinum (strain Langeland / NCTC 10281 / Type F) |
| A7GKN8 | Bacillus cytotoxicus (strain DSM 22905 / CIP 110041 / 391-98 / NVH 391-98) |
| A7VP38 | [Clostridium] leptum DSM 753 |
| A8MF94 | Alkaliphilus oremlandii (strain OhILAs) |
| A8SGA0 | Faecalibacterium prausnitzii M21/2 |
| A9KQQ0 | Lachnoclostridium phytofermentans (strain ATCC 700394 / DSM 18823 / ISDg) |
| A9VRL8 | Bacillus mycoides (strain KBAB4) |
| B0A768 | Intestinibacter bartlettii DSM 16795 |
| B0PGQ2 | Anaerotruncus colihominis DSM 17241 |
| B1B7L8 | Clostridium botulinum C str. Eklund |
| B1BUY9 | Clostridium perfringens E str. JGS1987 |
| B1HZY4 | Lysinibacillus sphaericus (strain C3-41) |
| B1I0Y4 | Desulforudis audaxviator (strain MP104C) |
| B1IFH8 | Clostridium botulinum (strain Okra / Type B1) |
| B1L220 | Clostridium botulinum (strain Loch Maree / Type A3) |
| B1V147 | Clostridium perfringens D str. JGS1721 |
| B2A234 | Natranaerobius thermophilus (strain ATCC BAA-1301 / DSM 18059 / JW/NM-WN-LF) |
| B2TR81 | Clostridium botulinum (strain Eklund 17B / Type B) |
| B7H7E6 | Bacillus cereus (strain B4264) |
| B7HT42 | Bacillus cereus (strain AH187) |
| B7IV22 | Bacillus cereus (strain G9842) |
| B7JN48 | Bacillus cereus (strain AH820) |
| B8D024 | Halothermothrix orenii (strain H 168 / OCM 544 / DSM 9562) |
| B8FWH0 | Desulfitobacterium hafniense (strain DSM 10664 / DCB-2) |
| B8FYY0 | Desulfitobacterium hafniense (strain DSM 10664 / DCB-2) |
| B8I518 | Ruminiclostridium cellulolyticum (strain ATCC 35319 / DSM 5812 / JCM 6584 / H10) |
| B9J2I6 | Bacillus cereus (strain Q1) |
| C0EC80 | [Clostridium] methylpentosum DSM 5476 |
| C0GEK8 | Dethiobacter alkaliphilus AHT 1 |
| C0GKQ5 | Dethiobacter alkaliphilus AHT 1 |
| C0Z7Y0 | Brevibacillus brevis (strain 47 / JCM 6285 / NBRC 100599) |
| C1FM06 | Clostridium botulinum (strain Kyoto / Type A2) |
| C2MFH9 | Bacillus cereus m1293 |
| C2NCC9 | Bacillus cereus BGSC 6E1 |
| C2NTK7 | Bacillus cereus 172560W |
| C2P9Q4 | Bacillus wiedmannii |
| C2Q4Q4 | Bacillus mycoides |
| C2R2U8 | Bacillus cereus m1550 |
| C2RYC7 | Bacillus cereus BDRD-ST26 |
| C2SET4 | Bacillus cereus BDRD-ST196 |
| C2SVM5 | Bacillus cereus BDRD-Cer4 |
| C2U8N8 | Bacillus cereus Rock1-15 |
| C2UQ90 | Bacillus cereus Rock3-28 |
| C2V6L5 | Bacillus cereus Rock3-29 |
| C2VND4 | Bacillus cereus Rock3-42 |
| C2W3H0 | Bacillus cereus Rock3-44 |
| C2WH64 | Bacillus cereus Rock4-2 |
| C2X6J6 | Bacillus cereus F65185 |
| C2XNT4 | Bacillus mycoides |
| C2YLE7 | Bacillus cereus AH1271 |
| C2Z2I2 | Bacillus cereus AH1272 |
| C3AGV8 | Bacillus pseudomycoides |
| C3BF79 | Bacillus pseudomycoides DSM 12442 |
| C3DEH4 | Bacillus thuringiensis serovar sotto str. T04001 |
| C3DYB8 | Bacillus thuringiensis serovar pakistani str. T13001 |
| C3FXV5 | Bacillus thuringiensis serovar andalousiensis BGSC 4AW1 |
| C3GDL8 | Bacillus thuringiensis serovar pondicheriensis BGSC 4BA1 |
| C3GVU6 | Bacillus thuringiensis serovar huazhongensis BGSC 4BD1 |
| C6BUC2 | Desulfovibrio salexigens (strain ATCC 14822 / DSM 2638 / NCIMB 8403 / VKM B-1763) |
| C6CZL4 | Paenibacillus sp. (strain JDR-2) |
| C6PNR2 | Clostridium carboxidivorans P7 |
| C7H4L3 | Faecalibacterium prausnitzii (strain DSM 17677 / JCM 31915 / A2-165) |
| D3EJ60 | Geobacillus sp. (strain Y412MC10) |
| D3FTK9 | Alkalihalobacillus pseudofirmus (strain ATCC BAA-2126 / JCM 17055 / OF4) |
| D4K392 | Faecalibacterium prausnitzii L2-6 |
| D4K5Q4 | Faecalibacterium prausnitzii SL3/3 |
| D4LCC1 | Ruminococcus champanellensis (strain DSM 18848 / JCM 17042 / KCTC 15320 / 18P13) |
| D5Q7I4 | Clostridioides difficile NAP08 |
| D5XCB8 | Thermincola potens (strain JR) |
| D7CMV3 | Syntrophothermus lipocalidus (strain DSM 12680 / TGB-C1) |
| D7CMW6 | Syntrophothermus lipocalidus (strain DSM 12680 / TGB-C1) |
| D7CPI5 | Syntrophothermus lipocalidus (strain DSM 12680 / TGB-C1) |
| D8GZN9 | Bacillus cereus var. anthracis (strain CI) |
| D9QSU7 | Acetohalobium arabaticum (strain ATCC 49924 / DSM 5501 / Z-7288) |
| D9S3F2 | Thermosediminibacter oceani (strain ATCC BAA-1034 / DSM 16646 / JW/IW-1228P) |
| D9SU30 | Clostridium cellulovorans (strain ATCC 35296 / DSM 3052 / OCM 3 / 743B) |
| E1JTH6 | Desulfovibrio fructosivorans JJ |
| E2ZIP0 | Faecalibacterium cf. prausnitzii KLE1255 |
| E3GYH6 | Methanothermus fervidus (strain ATCC 43054 / DSM 2088 / JCM 10308 / V24 S) |
| E5WH22 | Bacillus sp. 2_A_57_CT2 |
| E6SGM7 | Thermaerobacter marianensis (strain ATCC 700841 / DSM 12885 / JCM 10246 / 7p75a) |
| E6U242 | Bacillus cellulosilyticus (strain ATCC 21833 / DSM 2522 / FERM P-1141 / JCM 9156 / N-4) |
| E6U7S8 | Ethanoligenens harbinense (strain DSM 18485 / JCM 12961 / CGMCC 1.5033 / YUAN-3) |
| E6UIA5 | Ruminococcus albus (strain ATCC 27210 / DSM 20455 / JCM 14654 / NCDO 2250 / 7) |
| E9SC08 | Ruminococcus albus 8 |
| F0T282 | Syntrophobotulus glycolicus (strain DSM 8271 / FlGlyR) |
| F1TD32 | Ruminiclostridium papyrosolvens DSM 2782 |
| F2F5B4 | Solibacillus silvestris (strain StLB046) |
| F2JMP3 | Cellulosilyticum lentocellum (strain ATCC 49066 / DSM 5427 / NCIMB 11756 / RHM5) |
| F3M7Z8 | Paenibacillus sp. HGF5 |
| F3ZYE5 | Mahella australiensis (strain DSM 15567 / CIP 107919 / 50-1 BON) |
| F4LUV1 | Tepidanaerobacter acetatoxydans (strain DSM 21804 / JCM 16047 / Re1) |
| F4XDD8 | Ruminococcaceae bacterium D16 |
| F5L908 | Caldalkalibacillus thermarum (strain TA2.A1) |
| F6B7M9 | Desulfotomaculum nigrificans (strain DSM 14880 / VKM B-2319 / CO-1-SRB) |
| F6DV32 | Desulfotomaculum ruminis (strain ATCC 23193 / DSM 2154 / NCIMB 8452 / DL) |
| F7YZF7 | Bacillus coagulans (strain 2-6) |
| F9DRH8 | Sporosarcina newyorkensis 2681 |
| G2FZ51 | Desulfosporosinus sp. OT |
| G2IFS5 | Candidatus Arthromitus sp. SFB-rat-Yit |
| G2TI38 | Bacillus coagulans 36D1 |
| G4HDB3 | Paenibacillus lactis 154 |
| G4KNY6 | Oscillibacter valericigenes (strain DSM 18026 / NBRC 101213 / Sjm18-20) |
| G7VTU2 | Paenibacillus terrae (strain HPL-003) |
| G7WGC8 | Desulfosporosinus orientis (strain ATCC 19365 / DSM 765 / NCIMB 8382 / VKM B-1628 / Singapore I) |
| G8LX97 | Hungateiclostridium clariflavum (strain DSM 19732 / NBRC 101661 / EBR45) |
| G8LXQ4 | Hungateiclostridium clariflavum (strain DSM 19732 / NBRC 101661 / EBR45) |
| G8U0K6 | Sulfobacillus acidophilus (strain ATCC 700253 / DSM 10332 / NAL) |
| G9QEC9 | Bacillus sp. 7_6_55CFAA_CT2 |
| G9RY24 | Subdoligranulum sp. 4_3_54A2FAA |
| G9RY65 | Subdoligranulum sp. 4_3_54A2FAA |
| G9XMB5 | Desulfitobacterium hafniense DP7 |
| G9XTI7 | Desulfitobacterium hafniense DP7 |
| G9YNB0 | Flavonifractor plautii ATCC 29863 |
| G9YTS8 | Flavonifractor plautii ATCC 29863 |
| H0UHL3 | Brevibacillus laterosporus GI-9 |
| H1CJV2 | Lachnospiraceae bacterium 7_1_58FAA |
| H1CM65 | Lachnospiraceae bacterium 7_1_58FAA |
| H2JGD2 | Clostridium sp. BNL1100 |
| H5Y0H2 | Desulfosporosinus youngiae DSM 17734 |
| H5Y4C0 | Desulfosporosinus youngiae DSM 17734 |
| I3EBS4 | Bacillus methanolicus (strain MGA3 / ATCC 53907) |
| I4A9C6 | Desulfitobacterium dehalogenans (strain ATCC 51507 / DSM 9161 / JW/IU-DC1) |
| I4AEC7 | Desulfitobacterium dehalogenans (strain ATCC 51507 / DSM 9161 / JW/IU-DC1) |
| I4DCA6 | Desulfosporosinus acidiphilus (strain DSM 22704 / JCM 16185 / SJ4) |
| I7K7N2 | Caloramator australicus RC3 |
| I8UKI9 | Fictibacillus macauensis ZFHKF-1 |
| J0MVU4 | Clostridium sp. MSTE9 |
| J2HF29 | Brevibacillus sp. CF112 |
| J3A0M7 | Brevibacillus sp. BC25 |
| J3UQG8 | Bacillus thuringiensis HD-771 |
| J7J1X7 | Desulfosporosinus meridiei (strain ATCC BAA-275 / DSM 13257 / KCTC 12902 / NCIMB 13706 / S10) |
| J7TF58 | Clostridium sporogenes (strain ATCC 15579) |
| J7VVX4 | Bacillus cereus VD142 |
| J7W203 | Bacillus cereus VD022 |
| J8AN70 | Bacillus cereus BAG5X1-1 |
| J8B291 | Bacillus cereus BAG6X1-2 |
| J8CYF2 | Bacillus cereus HuA4-10 |
| J8EX71 | Bacillus cereus MC67 |
| J8GEI1 | Bacillus cereus MSX-D12 |
| J8HFT4 | Bacillus cereus VD014 |
| J8HJP5 | Bacillus cereus VD048 |
| J8HJY3 | Bacillus mycoides |
| J8HNV0 | Bacillus cereus VD045 |
| J8J8Y7 | Bacillus cereus VD107 |
| J8JXA8 | Bacillus cereus VD115 |
| J8KEJ8 | Bacillus cereus VD154 |
| J8L079 | Bacillus cereus VD166 |
| J8WRZ7 | Bacillus cereus BAG6O-2 |
| J9A3N1 | Bacillus wiedmannii |
| J9BNE1 | Bacillus cereus HuA2-1 |
| K0AYL8 | Gottschalkia acidurici (strain ATCC 7906 / DSM 604 / BCRC 14475 / CIP 104303 / KCTC 5404 / NCIMB 10678 / 9a) |
| K0FYJ3 | Bacillus thuringiensis MC28 |
| K0J7W6 | Amphibacillus xylanus (strain ATCC 51415 / DSM 6626 / JCM 7361 / LMG 17667 / NBRC 15112 / Ep01) |
| K1KWR9 | Solibacillus isronensis B3W22 |
| K1T604 | human gut metagenome |
| K4L163 | Dehalobacter sp. CF |
| K4L8A9 | Dehalobacter sp. CF |
| K4LJE9 | Thermacetogenium phaeum (strain ATCC BAA-254 / DSM 26808 / PB) |
| K4LJG8 | Thermacetogenium phaeum (strain ATCC BAA-254 / DSM 26808 / PB) |
| K4LS31 | Thermacetogenium phaeum (strain ATCC BAA-254 / DSM 26808 / PB) |
| K6CDL7 | Bacillus bataviensis LMG 21833 |
| K6DFM4 | Bacillus azotoformans LMG 9581 |
| K6PNB1 | Thermaerobacter subterraneus DSM 13965 |
| K8EHB8 | Desulfotomaculum hydrothermale Lam5 = DSM 18033 |
| L0F8H2 | Desulfitobacterium dichloroeliminans (strain LMG P-21439 / DCA1) |
| L0FCV5 | Desulfitobacterium dichloroeliminans (strain LMG P-21439 / DCA1) |
| L0K8D4 | Halobacteroides halobius (strain ATCC 35273 / DSM 5150 / MD-1) |
| L1QMY1 | Clostridium celatum DSM 1785 |
| L7VSI4 | Thermoclostridium stercorarium (strain ATCC 35414 / DSM 8532 / NCIMB 11754) |
| M1Q2M6 | uncultured organism |
| M1QFP2 | Bacillus thuringiensis serovar thuringiensis str. IS5056 |
| M1ZFQ2 | [Clostridium] ultunense Esp |
| M1ZWC4 | Clostridium botulinum CFSAN001627 |
| M7NH17 | Bhargavaea cecembensis DSE10 |
| M8ECB6 | Brevibacillus borstelensis AK1 |
| N1LY38 | Bacillus sp. GeD10 |
| N9WJ53 | Clostridium thermobutyricum |
| Q0AYS9 | Syntrophomonas wolfei subsp. wolfei (strain DSM 2245B / Goettingen) |
| Q0B0A3 | Syntrophomonas wolfei subsp. wolfei (strain DSM 2245B / Goettingen) |
| Q0SSR9 | Clostridium perfringens (strain SM101 / Type A) |
| Q180E2 | Clostridioides difficile (strain 630) |
| Q24MU3 | Desulfitobacterium hafniense (strain Y51) |
| Q24X43 | Desulfitobacterium hafniense (strain Y51) |
| Q2BDL5 | Bacillus sp. NRRL B-14911 |
| Q2RHM2 | Moorella thermoacetica (strain ATCC 39073 / JCM 9320) |
| Q2RIX9 | Moorella thermoacetica (strain ATCC 39073 / JCM 9320) |
| Q3EXK5 | Bacillus thuringiensis serovar israelensis ATCC 35646 |
| Q63GL9 | Bacillus cereus (strain ZK / E33L) |
| Q67P03 | Symbiobacterium thermophilum (strain T / IAM 14863) |
| Q6HP40 | Bacillus thuringiensis subsp. konkukian (strain 97-27) |
| Q73E97 | Bacillus cereus (strain ATCC 10987 / NRS 248) |
| Q81IJ0 | Bacillus cereus (strain ATCC 14579 / DSM 31 / CCUG 7414 / JCM 2152 / NBRC 15305 / NCIMB 9373 / NCTC 2599 / NRRL B-3711) |
| Q891C1 | Clostridium tetani (strain Massachusetts / E88) |
| Q8EMK7 | Oceanobacillus iheyensis (strain DSM 14371 / CIP 107618 / JCM 11309 / KCTC 3954 / HTE831) |
| Q8XK53 | Clostridium perfringens (strain 13 / Type A) |
| R1ATL3 | Caldisalinibacter kiritimatiensis |
| R1ATL9 | Caldisalinibacter kiritimatiensis |
| R4KF34 | Desulfallas gibsoniae DSM 7213 |
| R5AR75 | Firmicutes bacterium CAG:103 |
| R5D6S9 | Firmicutes bacterium CAG:555 |
| R5FGW9 | Faecalibacterium sp. CAG:1138 |
| R5H8Q9 | Firmicutes bacterium CAG:114 |
| R5IEV0 | Firmicutes bacterium CAG:124 |
| R5MXU2 | Eubacterium sp. CAG:180 |
| R5Q2B7 | Ruminococcus sp. CAG:724 |
| R5S576 | Firmicutes bacterium CAG:129 |
| R5VJ62 | Ruminococcus sp. CAG:254 |
| R5X722 | Clostridium bartlettii CAG:1329 |
| R5YSI8 | Ruminococcus sp. CAG:488 |
| R6BCN3 | Clostridium sp. CAG:169 |
| R6CKL4 | Clostridium sp. CAG:242 |
| R6DPJ1 | Firmicutes bacterium CAG:238 |
| R6DXU7 | Ruminococcus sp. CAG:563 |
| R6EN63 | Firmicutes bacterium CAG:145 |
| R6FYV5 | Clostridium sp. CAG:221 |
| R6IUK7 | Ruminococcus sp. CAG:177 |
| R6J393 | Firmicutes bacterium CAG:240 |
| R6KVE6 | Clostridium sp. CAG:265 |
| R6LRV5 | Firmicutes bacterium CAG:170 |
| R6NFJ6 | Clostridium sp. CAG:413 |
| R6P1I3 | Clostridium leptum CAG:27 |
| R6PYN2 | Faecalibacterium sp. CAG:82 |
| R6TCL0 | Ruminococcus sp. CAG:57 |
| R6U446 | Firmicutes bacterium CAG:272 |
| R6UPR3 | Oscillibacter sp. CAG:155 |
| R6VWU8 | Ruminococcus sp. CAG:382 |
| R6XR55 | Clostridium sp. CAG:349 |
| R7A017 | Ruminococcus sp. CAG:379 |
| R7BS74 | Firmicutes bacterium CAG:475 |
| R7FV06 | Eubacterium sp. CAG:841 |
| R7H5S4 | Ruminococcus sp. CAG:403 |
| R7K9N9 | Acidaminococcus sp. CAG:917 |
| R7L2L0 | Ruminococcus sp. CAG:353 |
| R7RPZ7 | Thermobrachium celere DSM 8682 |
| R7RQ10 | Thermobrachium celere DSM 8682 |
| R7ZIJ4 | Lysinibacillus sphaericus OT4b.31 |
| R8CKT0 | Bacillus cereus HuA3-9 |
| R8E529 | Bacillus cereus VD133 |
| R8GZ74 | Bacillus cereus VD196 |
| R8H0E9 | Bacillus cereus VD021 |
| R8I707 | Bacillus cereus BAG1O-1 |
| R8NGJ2 | Bacillus cereus HuB13-1 |
| R8NLF3 | Bacillus cereus (strain VD146) |
| R8PRB9 | Bacillus cereus VD136 |
| R8PTX8 | Bacillus cereus VDM053 |
| R8QZW5 | Bacillus cereus VD118 |
| R8SS86 | Bacillus cereus HuB4-4 |
| R8UIT8 | Bacillus cereus VD184 |
| R9CGG2 | Clostridium sartagoforme AAU1 |
| R9LI20 | Anaerotruncus sp. G3(2012) |
| R9LII4 | Anaerotruncus sp. G3(2012) |
| R9M946 | Oscillibacter sp. 1-3 |
| S0FWC8 | Ruminiclostridium cellobioparum subsp. termitidis CT1112 |
| S2YHJ1 | Paenisporosarcina sp. HGH0030 |
| S6EHR8 | Clostridium chauvoei JF4335 |
| T0JDL2 | Dehalobacter sp. UNSWDHB |
| T0PB17 | Clostridium sp. BL8 |
| T2RE34 | Paeniclostridium sordellii (strain ATCC 9714 / DSM 2141 / JCM 3814 / LMG 15708 / NCIMB 10717 / 211) |
| T2RMA2 | Dehalobacter sp. UNSWDHB |
| T3D8U9 | Clostridioides difficile CD160 |
| T4VKR6 | Paraclostridium bifermentans ATCC 638 |
| T4VVP0 | Paraclostridium bifermentans ATCC 19299 |
| U2CV98 | Clostridiales bacterium oral taxon 876 str. F0540 |
| U2DE89 | Clostridium sp. ATCC BAA-442 |
| U2EBP6 | Haloplasma contractile SSD-17B |
| U2N011 | Clostridium intestinale URNW |
| U2SIV8 | Oscillibacter sp. KLE 1745 |
| U4QZW2 | Ruminiclostridium papyrosolvens C7 |
| U5LGV4 | Bacillus infantis NRRL B-14911 |
| U6SKK9 | Bacillus marmarensis DSM 21297 |
| V2Y0S3 | Firmicutes bacterium ASF500 |
| V2YJ22 | Firmicutes bacterium ASF500 |
| V5M3G1 | Bacillus thuringiensis YBT-1518 |
| V6M837 | Brevibacillus panacihumi W25 |
| V6TC90 | Bacillus sp. 17376 |
| V9H1S1 | Clostridium sp. 7_2_43FAA |
| W0EBV3 | Desulfitobacterium metallireducens DSM 15288 |
| W0ECK7 | Desulfitobacterium metallireducens DSM 15288 |
| W0U3U4 | Ruminococcus bicirculans |
| W1SEP7 | Bacillus vireti LMG 21834 |
| W4C6U1 | Paenibacillus sp. FSL R7-269 |
| W4DW27 | Paenibacillus sp. FSL R7-277 |
| W4EZQ7 | Viridibacillus arenosi FSL R5-213 |
| W4Q9Q5 | Bacillus wakoensis JCM 9140 |
| W4QQM3 | Bacillus akibai (strain ATCC 43226 / DSM 21942 / JCM 9157 / 1139) |
| W4RL53 | Bacillus boroniphilus JCM 21738 |
| W4V9Q1 | Hungateiclostridium straminisolvens JCM 21531 |
| W7KY04 | Bacillus firmus DS1 |
| W7SEJ5 | Lysinibacillus sphaericus CBAM5 |
| W7YG53 | Paenibacillus pini JCM 16418 |
| W8Y4X0 | Bacillus thuringiensis DB27 |
| W9BER1 | Oceanobacillus picturae |
