# Supplementary material for: Dual‐wield NTPases: A novel protein family mined from AlphaFold DB
Source: Protein Sci. 2024 Mar 19;33(4):e4934. doi: 10.1002/pro.4934 (PMC10949312; doi:10.1002/pro.4934)
Supplement: Supplementary file 3 — Table S2. List of ESM metagenomic Atlas entries structurally related to dwNTPase. Note that the target database was culled by sequence similarity of 30% identity and predicted Local Distance Difference Test threshold. [file PRO-33-e4934-s003.docx]

Supplementary Data Table 2: List of ESM metagenomic Atlas entries structurally related to dwNTPase. Note that the target database was culled by sequence similarity of 30% identity and pLDDT threshold.

| MGYP000005464415 |
| --- |
| MGYP000011566305 |
| MGYP000011743424 |
| MGYP000013930452 |
| MGYP000021985546 |
| MGYP000022775823 |
| MGYP000031243067 |
| MGYP000032816584 |
| MGYP000035039818 |
| MGYP000039754873 |
| MGYP000046075001 |
| MGYP000046921890 |
| MGYP000050773439 |
| MGYP000056714802 |
| MGYP000058475240 |
| MGYP000063702171 |
| MGYP000072106316 |
| MGYP000073817642 |
| MGYP000073976865 |
| MGYP000074926707 |
| MGYP000087961665 |
| MGYP000090814326 |
| MGYP000095660081 |
| MGYP000097934424 |
| MGYP000100781166 |
| MGYP000110243900 |
| MGYP000121404939 |
| MGYP000132674256 |
| MGYP000133036970 |
| MGYP000137384458 |
| MGYP000137884487 |
| MGYP000138095260 |
| MGYP000144053071 |
| MGYP000149276925 |
| MGYP000152158533 |
| MGYP000155467865 |
| MGYP000158031104 |
| MGYP000158701616 |
| MGYP000161370762 |
| MGYP000172069787 |
| MGYP000179168143 |
| MGYP000182400474 |
| MGYP000187260450 |
| MGYP000196473938 |
| MGYP000203777768 |
| MGYP000206671460 |
| MGYP000209962063 |
| MGYP000213213017 |
| MGYP000214913979 |
| MGYP000223653340 |
| MGYP000226615218 |
| MGYP000229661469 |
| MGYP000233862775 |
| MGYP000260203210 |
| MGYP000267655121 |
| MGYP000276180941 |
| MGYP000281675394 |
| MGYP000283494147 |
| MGYP000289560546 |
| MGYP000297351230 |
| MGYP000307241419 |
| MGYP000316400542 |
| MGYP000317455607 |
| MGYP000329090296 |
| MGYP000329155775 |
| MGYP000332792456 |
| MGYP000338656182 |
| MGYP000338766891 |
| MGYP000338885734 |
| MGYP000339528302 |
| MGYP000341965729 |
| MGYP000344960023 |
| MGYP000349730980 |
| MGYP000356359273 |
| MGYP000357322122 |
| MGYP000362356478 |
| MGYP000365138801 |
| MGYP000366121951 |
| MGYP000369246366 |
| MGYP000369248057 |
| MGYP000374059993 |
| MGYP000376728391 |
| MGYP000377349853 |
| MGYP000380150569 |
| MGYP000380918257 |
| MGYP000387215517 |
| MGYP000389137078 |
| MGYP000392093694 |
| MGYP000392322862 |
| MGYP000397624302 |
| MGYP000412013239 |
| MGYP000414862069 |
| MGYP000415449340 |
| MGYP000415610050 |
| MGYP000418125265 |
| MGYP000424919781 |
| MGYP000425215761 |
| MGYP000427578989 |
| MGYP000432980634 |
| MGYP000434790480 |
| MGYP000440675606 |
| MGYP000449267722 |
| MGYP000453218129 |
| MGYP000468120591 |
| MGYP000479063216 |
| MGYP000481223319 |
| MGYP000495801975 |
| MGYP000501602264 |
| MGYP000516580646 |
| MGYP000518541956 |
| MGYP000522568599 |
| MGYP000540603521 |
| MGYP000544980939 |
| MGYP000554953503 |
| MGYP000560810235 |
| MGYP000563868109 |
| MGYP000565819564 |
| MGYP000570892856 |
| MGYP000573565277 |
| MGYP000580686002 |
| MGYP000583596164 |
| MGYP000598313075 |
| MGYP000609899509 |
| MGYP000610054608 |
| MGYP000619824976 |
| MGYP000621160421 |
| MGYP000624467883 |
| MGYP000624854459 |
| MGYP000626659218 |
| MGYP000628622447 |
| MGYP000633643098 |
| MGYP000635340560 |
| MGYP000637753403 |
| MGYP000640418712 |
| MGYP000651216849 |
| MGYP000656668235 |
| MGYP000665329988 |
| MGYP000668644108 |
| MGYP000671723777 |
| MGYP000672861390 |
| MGYP000674781713 |
| MGYP000680700556 |
| MGYP000680727538 |
| MGYP000687400265 |
| MGYP000688418485 |
| MGYP000692424004 |
| MGYP000694260436 |
| MGYP000712421253 |
| MGYP000712598401 |
| MGYP000715366489 |
| MGYP000716100718 |
| MGYP000716110458 |
| MGYP000719069569 |
| MGYP000719259666 |
| MGYP000720987161 |
| MGYP000722397558 |
| MGYP000737789640 |
| MGYP000745950594 |
| MGYP000760084081 |
| MGYP000766970156 |
| MGYP000773444244 |
| MGYP000776980989 |
| MGYP000779413157 |
| MGYP000783606972 |
| MGYP000794234970 |
| MGYP000794583975 |
| MGYP000796842517 |
| MGYP000800695632 |
| MGYP000818155992 |
| MGYP000819680346 |
| MGYP000829006276 |
| MGYP000833698882 |
| MGYP000837620560 |
| MGYP000845675048 |
| MGYP000846291321 |
| MGYP000846738100 |
| MGYP000847433322 |
| MGYP000850522706 |
| MGYP000854750270 |
| MGYP000855463610 |
| MGYP000856935708 |
| MGYP000859444552 |
| MGYP000860510931 |
| MGYP000862261671 |
| MGYP000862978595 |
| MGYP000863910226 |
| MGYP000864615565 |
| MGYP000865798123 |
| MGYP000866159967 |
| MGYP000870042540 |
| MGYP000872154735 |
| MGYP000873111992 |
| MGYP000873114169 |
| MGYP000873298085 |
| MGYP000873570358 |
| MGYP000874498255 |
| MGYP000875228748 |
| MGYP000876619059 |
| MGYP000881585069 |
| MGYP000882995862 |
| MGYP000883449302 |
| MGYP000884151462 |
| MGYP000884157304 |
| MGYP000886298914 |
| MGYP000886527711 |
| MGYP000887942385 |
| MGYP000889347843 |
| MGYP000891638238 |
| MGYP000893577897 |
| MGYP000893585008 |
| MGYP000896138925 |
| MGYP000896152022 |
| MGYP000902764005 |
| MGYP000905320016 |
| MGYP000905590446 |
| MGYP000906464546 |
| MGYP000911693974 |
| MGYP000912395745 |
| MGYP000913366715 |
| MGYP000917325413 |
| MGYP000918987734 |
| MGYP000920861653 |
| MGYP000927488903 |
| MGYP000929340741 |
| MGYP000929694189 |
| MGYP000932862644 |
| MGYP000934286412 |
| MGYP000937807594 |
| MGYP000938080560 |
| MGYP000939256813 |
| MGYP000943814674 |
| MGYP000944892989 |
| MGYP000946285437 |
| MGYP000948302140 |
| MGYP000948387078 |
| MGYP000948665050 |
| MGYP000948773478 |
| MGYP000949039413 |
| MGYP000950522601 |
| MGYP000950781180 |
| MGYP000952638058 |
| MGYP000953940834 |
| MGYP000954761325 |
| MGYP000956436473 |
| MGYP000965532308 |
| MGYP000966778367 |
| MGYP000967381621 |
| MGYP000967452070 |
| MGYP000969341437 |
| MGYP000971973798 |
| MGYP000981829549 |
| MGYP000982030885 |
| MGYP000982564017 |
| MGYP000983360511 |
| MGYP000983698559 |
| MGYP000983968897 |
| MGYP000984007444 |
| MGYP000984069175 |
| MGYP000986005084 |
| MGYP000986772698 |
| MGYP000987235152 |
| MGYP000987950999 |
| MGYP000989346284 |
| MGYP000991037961 |
| MGYP000993126788 |
| MGYP000996428994 |
| MGYP000998089494 |
| MGYP000999504741 |
| MGYP001000915644 |
| MGYP001001350963 |
| MGYP001002397009 |
| MGYP001003736678 |
| MGYP001004190013 |
| MGYP001004907671 |
| MGYP001008415023 |
| MGYP001009845231 |
| MGYP001010016555 |
| MGYP001010081099 |
| MGYP001010801421 |
| MGYP001012929272 |
| MGYP001015136735 |
| MGYP001018576274 |
| MGYP001019263009 |
| MGYP001019466318 |
| MGYP001022181482 |
| MGYP001022569621 |
| MGYP001023499417 |
| MGYP001024206109 |
| MGYP001031223130 |
| MGYP001032263427 |
| MGYP001033019173 |
| MGYP001036146819 |
| MGYP001037691505 |
| MGYP001041136564 |
| MGYP001044408008 |
| MGYP001044653672 |
| MGYP001046357902 |
| MGYP001051366889 |
| MGYP001051616043 |
| MGYP001052389888 |
| MGYP001053934307 |
| MGYP001054709820 |
| MGYP001055124250 |
| MGYP001057389601 |
| MGYP001057713172 |
| MGYP001057939588 |
| MGYP001058076732 |
| MGYP001066949444 |
| MGYP001067596079 |
| MGYP001067597097 |
| MGYP001070558268 |
| MGYP001071062081 |
| MGYP001072290625 |
| MGYP001072290727 |
| MGYP001072684865 |
| MGYP001074592748 |
| MGYP001074594893 |
| MGYP001075631168 |
| MGYP001076246188 |
| MGYP001076286658 |
| MGYP001077760211 |
| MGYP001078469938 |
| MGYP001081539167 |
| MGYP001083094903 |
| MGYP001086549199 |
| MGYP001087267426 |
| MGYP001089574896 |
| MGYP001095254367 |
| MGYP001097077469 |
| MGYP001099651039 |
| MGYP001101482462 |
| MGYP001102279279 |
| MGYP001104087068 |
| MGYP001105578980 |
| MGYP001111995788 |
| MGYP001118306118 |
| MGYP001119547012 |
| MGYP001120777949 |
| MGYP001122123002 |
| MGYP001124793146 |
| MGYP001130624379 |
| MGYP001132418681 |
| MGYP001136708707 |
| MGYP001139646309 |
| MGYP001140236529 |
| MGYP001140486808 |
| MGYP001141881270 |
| MGYP001144037523 |
| MGYP001147184671 |
| MGYP001147701674 |
| MGYP001148728985 |
| MGYP001151314202 |
| MGYP001153095883 |
| MGYP001153238483 |
| MGYP001153393980 |
| MGYP001153741911 |
| MGYP001158822045 |
| MGYP001163045142 |
| MGYP001167325490 |
| MGYP001170022268 |
| MGYP001173886188 |
| MGYP001178305136 |
| MGYP001190237233 |
| MGYP001193054289 |
| MGYP001198073675 |
| MGYP001199854867 |
| MGYP001203888545 |
| MGYP001205242401 |
| MGYP001208954122 |
| MGYP001212037890 |
| MGYP001216256983 |
| MGYP001226913156 |
| MGYP001229757033 |
| MGYP001230977503 |
| MGYP001236415831 |
| MGYP001238894987 |
| MGYP001240458690 |
| MGYP001241439385 |
| MGYP001241830453 |
| MGYP001244424814 |
| MGYP001245476879 |
| MGYP001246862665 |
| MGYP001251410889 |
| MGYP001256856623 |
| MGYP001258079375 |
| MGYP001260779301 |
| MGYP001266339757 |
| MGYP001270284586 |
| MGYP001274359445 |
| MGYP001282449832 |
| MGYP001293271363 |
| MGYP001298300523 |
| MGYP001298735028 |
| MGYP001306662269 |
| MGYP001308373670 |
| MGYP001310676587 |
| MGYP001316291143 |
| MGYP001317270614 |
| MGYP001317651948 |
| MGYP001321326270 |
| MGYP001324221372 |
| MGYP001325391279 |
| MGYP001328123680 |
| MGYP001335462562 |
| MGYP001343381432 |
| MGYP001343531413 |
| MGYP001347097104 |
| MGYP001349776869 |
| MGYP001350155789 |
| MGYP001370477403 |
| MGYP001375895633 |
| MGYP001375895852 |
| MGYP001377036890 |
| MGYP001377380736 |
| MGYP001386727829 |
| MGYP001389434110 |
| MGYP001390933540 |
| MGYP001394707573 |
| MGYP001397346626 |
| MGYP001400061178 |
| MGYP001407994366 |
| MGYP001408430282 |
| MGYP001410709774 |
| MGYP001411239173 |
| MGYP001416624695 |
| MGYP001430084181 |
| MGYP001431026079 |
| MGYP001431417008 |
| MGYP001432564324 |
| MGYP001437982201 |
| MGYP001446108616 |
| MGYP001464086068 |
| MGYP001465057784 |
| MGYP001470679893 |
| MGYP001472048744 |
| MGYP001480157240 |
| MGYP001481512249 |
| MGYP001482463118 |
| MGYP001484212884 |
| MGYP001484214231 |
| MGYP001486922552 |
| MGYP001487875866 |
| MGYP001516528669 |
| MGYP001522023819 |
| MGYP001535160546 |
| MGYP001537081954 |
| MGYP001538263980 |
| MGYP001598895366 |
| MGYP001601149468 |
| MGYP001620285363 |
| MGYP001624020629 |
| MGYP001625272499 |
| MGYP001630033458 |
| MGYP001630426298 |
| MGYP001630889881 |
| MGYP001632728344 |
| MGYP001638034195 |
| MGYP001639344602 |
| MGYP001657894984 |
| MGYP001770710926 |
| MGYP001771164780 |
| MGYP001771283061 |
| MGYP001774661584 |
| MGYP001775303000 |
| MGYP001776077675 |
| MGYP001777284512 |
| MGYP001777798491 |
| MGYP001777999770 |
| MGYP001778362933 |
| MGYP001780621392 |
| MGYP001781026291 |
| MGYP001783202305 |
| MGYP001783919854 |
| MGYP001829439330 |
| MGYP001851345070 |
| MGYP001852003032 |
| MGYP001869686281 |
| MGYP001944123563 |
| MGYP001944327984 |
| MGYP001952770736 |
| MGYP001953138799 |
| MGYP001953984248 |
| MGYP001954818258 |
| MGYP002268305163 |
| MGYP002348050470 |
| MGYP002388813658 |
| MGYP002398377235 |
| MGYP002407614603 |
| MGYP002409113681 |
| MGYP002410704882 |
| MGYP002505411068 |
| MGYP002508460163 |
| MGYP002508579337 |
| MGYP002509702026 |
| MGYP002509779980 |
| MGYP002510528239 |
| MGYP002512426959 |
| MGYP002512774708 |
| MGYP002513981819 |
| MGYP002514523331 |
| MGYP002514735286 |
| MGYP002515637646 |
| MGYP002516001785 |
| MGYP002516138547 |
| MGYP002516672976 |
| MGYP002516965467 |
| MGYP002517707201 |
| MGYP002518695557 |
| MGYP002518877164 |
| MGYP002518946026 |
| MGYP002519335693 |
| MGYP002519536267 |
| MGYP002519891592 |
| MGYP002520059971 |
| MGYP002520388537 |
| MGYP002520843664 |
| MGYP002521152857 |
| MGYP002521245368 |
| MGYP002521741868 |
| MGYP002521823067 |
| MGYP002522324169 |
| MGYP002523679548 |
| MGYP002526212556 |
| MGYP002531393319 |
| MGYP002535785097 |
| MGYP002536951133 |
| MGYP002538392298 |
| MGYP002540006505 |
| MGYP002542236210 |
| MGYP002544833878 |
| MGYP002545730500 |
| MGYP002546544705 |
| MGYP002547882387 |
| MGYP002548274122 |
| MGYP002548845381 |
| MGYP002550710106 |
| MGYP002551048834 |
| MGYP002552576849 |
| MGYP002553774068 |
| MGYP002554244177 |
| MGYP002557624238 |
| MGYP002563089458 |
| MGYP002563477401 |
| MGYP002564862066 |
| MGYP002565572074 |
| MGYP002565576012 |
| MGYP002569519302 |
| MGYP002569928787 |
| MGYP002570950553 |
| MGYP002572209789 |
| MGYP002580689582 |
| MGYP002584680938 |
| MGYP002584950757 |
| MGYP002604693242 |
| MGYP002625390995 |
| MGYP002626635166 |
| MGYP002647315051 |
| MGYP002647372959 |
| MGYP002648415025 |
| MGYP002655976423 |
| MGYP002672574807 |
| MGYP002672701855 |
| MGYP002675175697 |
| MGYP002679840418 |
| MGYP002705934524 |
| MGYP002710575024 |
| MGYP002728503105 |
| MGYP002731537162 |
| MGYP002732731958 |
| MGYP002733154163 |
| MGYP002733396199 |
| MGYP002735076069 |
| MGYP002748286501 |
| MGYP002751119144 |
| MGYP002753153100 |
| MGYP002797240956 |
| MGYP002798388229 |
| MGYP002802551986 |
| MGYP002857010239 |
| MGYP002867796410 |
| MGYP002903955578 |
| MGYP002960176274 |
| MGYP002972649115 |
| MGYP003013740277 |
| MGYP003101485480 |
| MGYP003104526251 |
| MGYP003162439181 |
| MGYP003181990754 |
| MGYP003184855784 |
| MGYP003185590153 |
| MGYP003195452491 |
| MGYP003209727232 |
| MGYP003210669313 |
| MGYP003220877510 |
| MGYP003266687897 |
| MGYP003280304731 |
| MGYP003283676434 |
| MGYP003288713578 |
| MGYP003288879220 |
| MGYP003289010650 |
| MGYP003289121590 |
| MGYP003289460875 |
| MGYP003289554861 |
| MGYP003289654488 |
| MGYP003289736391 |
| MGYP003290012312 |
| MGYP003290061622 |
| MGYP003290476675 |
| MGYP003290532250 |
| MGYP003290763530 |
| MGYP003290847745 |
| MGYP003290873295 |
| MGYP003290982398 |
| MGYP003291326079 |
| MGYP003291517417 |
| MGYP003291711009 |
| MGYP003291766561 |
| MGYP003292095451 |
| MGYP003292202049 |
| MGYP003292347188 |
| MGYP003293279942 |
| MGYP003293463256 |
| MGYP003293661037 |
| MGYP003294162239 |
| MGYP003294245754 |
| MGYP003294696003 |
| MGYP003295022881 |
| MGYP003295543339 |
| MGYP003295677572 |
| MGYP003295683491 |
| MGYP003296276390 |
| MGYP003296445675 |
| MGYP003297612339 |
| MGYP003297636011 |
| MGYP003298888900 |
| MGYP003299105919 |
| MGYP003299134100 |
| MGYP003299421817 |
| MGYP003299624148 |
| MGYP003299798750 |
| MGYP003300215213 |
| MGYP003301305664 |
| MGYP003301463800 |
| MGYP003302090907 |
| MGYP003302257712 |
| MGYP003302551956 |
| MGYP003302844486 |
| MGYP003303284055 |
| MGYP003303453267 |
| MGYP003303602865 |
| MGYP003303613744 |
| MGYP003303857016 |
| MGYP003303883274 |
| MGYP003304453194 |
| MGYP003304735657 |
| MGYP003305165249 |
| MGYP003305203406 |
| MGYP003305708281 |
| MGYP003306062031 |
| MGYP003307021237 |
| MGYP003307705220 |
| MGYP003307919367 |
| MGYP003308042158 |
| MGYP003308684829 |
| MGYP003308840078 |
| MGYP003308867545 |
| MGYP003308877326 |
| MGYP003309211794 |
| MGYP003309457844 |
| MGYP003309930354 |
| MGYP003310390468 |
| MGYP003310719773 |
| MGYP003311209964 |
| MGYP003311456034 |
| MGYP003311669213 |
| MGYP003315797261 |
| MGYP003315959051 |
| MGYP003316642960 |
| MGYP003316655320 |
| MGYP003317707224 |
| MGYP003318230597 |
| MGYP003318922623 |
| MGYP003319073510 |
| MGYP003319556249 |
| MGYP003319980098 |
| MGYP003324755819 |
| MGYP003329257096 |
| MGYP003339773965 |
| MGYP003345614305 |
| MGYP003362406859 |
| MGYP003365329244 |
| MGYP003369291436 |
| MGYP003369572669 |
| MGYP003369774084 |
| MGYP003369841141 |
| MGYP003371456443 |
| MGYP003373848938 |
| MGYP003375948820 |
| MGYP003376599254 |
| MGYP003377696461 |
| MGYP003378316808 |
| MGYP003379681987 |
| MGYP003385241863 |
| MGYP003385333612 |
| MGYP003393809899 |
| MGYP003400006688 |
| MGYP003421748675 |
| MGYP003427310168 |
| MGYP003430666403 |
| MGYP003430775767 |
| MGYP003435514729 |
| MGYP003447008246 |
| MGYP003466133340 |
| MGYP003467745854 |
| MGYP003474320691 |
| MGYP003482910043 |
| MGYP003484242520 |
| MGYP003508593118 |
| MGYP003509481182 |
| MGYP003510406608 |
| MGYP003515515405 |
| MGYP003518594222 |
| MGYP003520657654 |
| MGYP003522064156 |
| MGYP003526856239 |
| MGYP003528103500 |
| MGYP003530985645 |
| MGYP003531931634 |
| MGYP003533196885 |
| MGYP003533852953 |
| MGYP003542505439 |
| MGYP003547168537 |
| MGYP003550500512 |
| MGYP003552847930 |
| MGYP003558080982 |
| MGYP003558878903 |
| MGYP003564231365 |
| MGYP003571335074 |
| MGYP003585492775 |
| MGYP003592361735 |
| MGYP003623391267 |
| MGYP003623576751 |
| MGYP003623868684 |
| MGYP003764291581 |
| MGYP003783283803 |
| MGYP003819602827 |
| MGYP003900866323 |
| MGYP003901033641 |
